# Genomic plasticity of pathogenic *Escherichia coli* mediates d-serine tolerance via multiple adaptive mechanisms

**DOI:** 10.1073/pnas.2004977117

**Published:** 2020-08-26

**Authors:** Nicky O’Boyle, James P. R. Connolly, Nicholas P. Tucker, Andrew J. Roe

**Affiliations:** ^a^Institute of Infection, Immunity and Inflammation, University of Glasgow, G12 8TA Glasgow, United Kingdom;; ^b^Newcastle University Biosciences Institute, Newcastle University, NE2 4HH Newcastle-upon-Tyne, United Kingdom;; ^c^Strathclyde Institute of Pharmacy and Biomedical Sciences, University of Strathclyde, G4 0RE Glasgow, United Kingdom

**Keywords:** d-amino acids, EHEC, adaptive evolution, metabolism, virulence

## Abstract

The molecular environment of the host can have profound effects on the behavior of resident bacterial species. We recently established how the sensing and response of enterohemorrhagic *Escherichia coli* (EHEC) to d-serine (d-Ser) resulted in down-regulation of type 3 secretion system-dependent colonization, thereby avoiding unfavorable environments abundant in this toxic metabolite. However, this model ignores a key determinant of the success of bacterial pathogens, adaptive evolution. In this study, we have explored the adaptation of EHEC to d-Ser and its consequences for pathogenesis. We rapidly isolated multiple, independent, EHEC mutants whose growth was no longer compromised in the presence of d-Ser. Through a combination of whole-genome sequencing and transcriptomics, we showed that tolerance could be attributed to disruption of one of two d-Ser transporters and/or activation of a previously nonfunctional d-Ser deaminase. While the implication of cytoplasmic transport in d-Ser toxicity was unsurprising, disruption of a single transporter, CycA, was sufficient to completely overcome the repression of type 3 secretion system activity normally associated with exposure to d-Ser. Despite the fact that this reveals a mechanism by which evolution could drive a pathogen to colonize new niches, interrogation of sequenced *E. coli* O157:H7 genomes showed a high level of CycA conservation, highlighting a strong selective pressure for functionality. Collectively, these data show that CycA is a critically important conduit for d-Ser uptake that is central to the niche restriction of EHEC.

## Significance

Pathogens ensure infection of favored sites in the body by responding to chemical signals. One chemical abundant in urine, the amino acid d-Ser, is toxic to EHEC and reduces expression of the machinery used for host cell attachment, making the bladder an unfavorable environment. We observed that under d-Ser stress, EHEC acquires genetic changes that lead to blocking d-Ser uptake into the cell or activating a silent enzyme for degrading d-Ser. This prevents growth inhibition and, critically, inhibits the repression of attachment machinery normally caused by d-Ser. These findings highlight the importance of pathogen evolution in determining how host molecules regulate colonization. These interactions underpin a process known as niche restriction that is important for pathogen success within the host.

Pathogenic bacteria sense and respond to a plethora of signals as they traverse through the host. The integration of these signals into appropriate changes in behavior is an important determinant of a pathogen’s success. The intestine is a particularly complex and dynamic environment, containing signals from the host, the microbiome, and large molecular perturbations from diet. Competition within this environment extends beyond simply contesting for food, as metabolites can influence gene expression and even evolutionary adaptation of the bacteria within the gut.

Free d-amino acids represent an often-overlooked aspect of the human diet. Dietary consumption of d-amino acids has been estimated at 100 mg/d (1). These compounds, unlike their l-enantiomers, require specialized catabolic pathways in order to be used as energy sources (2–7), and as such represent an important part of nutritional competition and metabolic signaling to enteric species. The peptidoglycan sacculus of gram-negative bacteria is strengthened by cross-links containing d-alanine and d-glutamate (8). It has been demonstrated that some bacteria possess the ability to produce a large number of additional d-amino acids by virtue of a broad-spectrum racemase and these compounds play a role in regulating stationary-phase cell-wall structure (9). The levels of d-amino acids vary at different sites within the host, with d-serine (d-Ser), for example, being found at ∼1 μM in the gut (10) and ∼1 mM in the urine (11), where it is the most abundant d-amino acid (12). d-Ser is also found at localized high concentrations in certain brain tissues, where it functions as a neurotransmitter by binding to *N*-methyl d-aspartate receptors (13).

Extraintestinal pathogenic *Escherichia coli* (ExPEC), a group that is primarily composed of uropathogenic *E. coli* (UPEC) and neonatal meningitis-associated *E. coli* (NMEC), exhibit the ability to disseminate from the intestine and establish infection at extraintestinal sites (14). Conversely, the attaching/effacing (A/E) pathogens, so-called due to their formation of pedestal-like lesions on the surface of infected cells, are normally restricted to the intestine (15). Colonization by this group, which includes enterohemorrhagic *E. coli* (EHEC), is dependent upon the locus of enterocyte effacement (LEE)-encoded type 3 secretion system (16). Catabolism of d-Ser in *E. coli* is facilitated by the *dsdCXA* locus that comprises a transcriptional activator, inner-membrane transporter, and deaminase/dehydratase (3). While up to 85% of pyelonephritis and urosepsis isolates have been found to possess an intact *dsdCXA* locus (11), only 5.7% of isolates possessing the LEE-encoded type 3 secretion system carried an intact locus (10). In the case of the prototype EHEC isolate EDL933, a sucrose utilization operon encoded by *cscBKAR* has been acquired by recombination, resulting in the loss of the transcriptional activator-encoding gene *dsdC* and an inactivating truncation of the transporter-encoding gene *dsdX* (17). Without DsdC the system is nonfunctional, resulting in d-Ser toxicity. The extremely rare concurrent carriage of the LEE and *dsd* systems indicates a strong pressure for A/E pathogens to avoid environments with high levels of d-Ser. A number of inner-membrane transporters have been implicated in specific uptake of d-Ser, including DsdX (18), which is encoded in the *dsdCXA* locus, CycA (19), and SstT (20).

We recently described how EHEC can sense d-Ser (21) and respond by reducing expression of its primary colonization apparatus, the LEE-encoded type 3 secretion system (10). This allows for restriction of EHEC to a more favorable niche within the gut where d-Ser concentrations are low (10), compared with environments such as the bladder, where d-Ser levels are higher (11). In addition to virulence repression by d-Ser, an SOS-like response is activated, leading to a higher rate of genomic variability (22), likely reflecting the bacterium attempting to overcome the stress induced by d-Ser exposure. Together with the observation that LEE-positive enteric *E. coli* have evolved to lose the ability to catabolize d-Ser (10), these findings corroborate the role of d-Ser in niche restriction of LEE-positive *E. coli*.

Here we expand on these studies by investigating if EHEC can adapt to overcome this evolutionary bottleneck. We describe the genetic and phenotypic consequences of repeated in vitro exposure of EHEC to d-Ser. Tolerance to d-Ser convergently evolved by distinct mechanisms and resulted in an ability to overcome not only the growth-inhibitory effects of d-Ser but also the majority of transcriptional alterations associated with d-Ser, including type 3 secretion system-dependent colonization. We discuss these mechanistic insights and the implications that they have for the pathogenic lifestyle of EHEC.

## Results

### d-Ser Exposure Can Induce the Evolution of Tolerant EHEC Isolates.

**Fig. 1.**
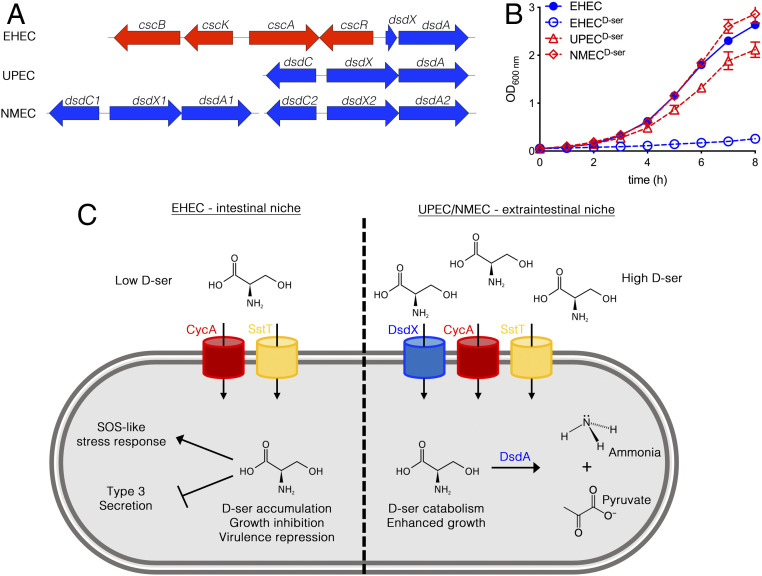
EHEC is unable to tolerate d-Ser due to an insertion that truncates the d-Ser catabolism locus. (*A*) Schematic depicting the d-Ser catabolism locus in EHEC, UPEC, and NMEC. (*B*) Growth curves of EHEC, UPEC, and NMEC in M9 + glucose (Glc) ± 1 mM d-Ser. Error bars indicate SEM from a minimum of three experiments. (*C*) Schematic illustrating the contrasting effects of d-Ser on *dsd*− and *dsd*+ strains of *E. coli*. While d-Ser catabolism promotes the growth of UPEC and NMEC, growth of EHEC is severely restricted by d-Ser. In the case of EHEC, d-Ser is transported into the cytosol by CycA and SstT, where it accumulates and induces an SOS-like response and transcriptional repression of type 3 secretion.

**Fig. 2.**
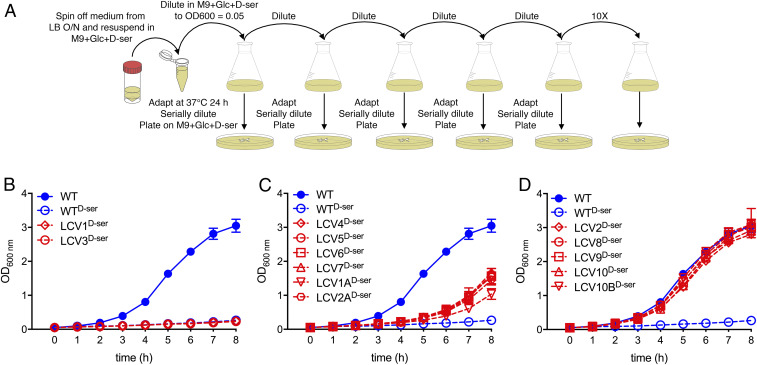
d-Ser–tolerant EHEC mutants evolve and can be selected for by repeated culture in the presence of d-Ser. (*A*) Experimental workflow of repeated batch culture for the enrichment of d-Ser–tolerant mutants. (*B*–*D*) Growth curves (M9 + glucose + 1 mM d-Ser) of apparent LCV clones obtained after the indicated number of repeat cultures in d-Ser–containing medium. Error bars indicate SEM from a minimum of three experiments.

The prototype UPEC and NMEC strains CFT073 and CE10 were chosen for inclusion in this study as, like 85% of UPEC (11) and 97.5% of NMEC (23), they possess complete, responsive, and functional *dsd* loci (Fig. 1*A*). The NMEC CE10 isolate is interesting as it carries two copies of the locus. EHEC TUV93-0 (a Shiga toxin-negative derivative of EDL933), on the other hand, is typical of the majority of diarrheagenic LEE-pathogenicity island-carrying *E. coli* in that it possesses a genomic rearrangement upstream of *dsdA* leading to loss of *dsdCX* and hence loss of function (10, 23). EHEC cannot grow on d-Ser as a sole carbon source, and its growth in glucose-containing minimal medium is strongly inhibited by d-Ser (Fig. 1 *B* and *C*). The specific growth rate (SGR) for EHEC was found to decrease from 0.63 h^−1^, in agreement with previous reports for M9 + glucose (24), to 0.19 h^−1^ (*P* = 0.003) upon inclusion of d-Ser, whereas no significant decrease was observed with UPEC or NMEC (*SI Appendix*, Fig. S1). This indicated that d-Ser exerted a sublethal growth-inhibitory effect on EHEC and alluded to the possibility that tolerant mutants could arise by repeated exposure as the bacteria attempt to overcome the inhibition of growth. To test this, we repeatedly batch-cultured EHEC in the presence of d-Ser for a period of 10 d as outlined in Fig. 2*A*. When subculturing on each day, a sample was serially diluted and spot-plated on solid M9 medium containing glucose and d-Ser (i.e., the same selective conditions as the flask culture). Importantly, this allowed for discrimination of putatively tolerant clones by virtue of their ability to form large colonies. The resulting clones were termed large-colony variants (LCVs) and numbers were assigned from the passage number at which they were isolated (for example, LCV3 was isolated from the third 24-h batch culture). LCV1A and 2A were isolated from a repeat experiment with two successive batch cultures, while LCV10B represented a second colony selected for analysis from the 10-d culture.

In order to validate the selection process, pure cultures of each isolate were tested for growth rate in d-Ser–containing medium. Two early clones (LCV1 and LCV3) appeared to be false positives as no tolerance to d-Ser was observed (Fig. 2*B* and *SI Appendix*, Fig. S2). Six clones (LCV4 to 7, 1A, and 2A) displayed intermediate levels of tolerance (SGR significantly higher than wild-type [WT] d-Ser but lower than WT, 0.31 to 0.38 h^−1^; Fig. 2*C* and *SI Appendix*, Fig. S2). The remaining five clones (LCV2, 8 to 10, and 10B) displayed complete tolerance to d-Ser with growth rates similar to that of WT EHEC cultured in the absence of d-Ser (Fig. 2*C* and *SI Appendix*, Fig. S2). As such, d-Ser–tolerant mutants can be isolated and positively selected for by repeated culture in the presence of toxic d-Ser. Strikingly, the observation of two distinct levels of tolerance alluded to at least two independent mechanisms of tolerance.

### The Evolution of Tolerance Alters the Transcriptional Response to d-Ser.

**Fig. 3.**
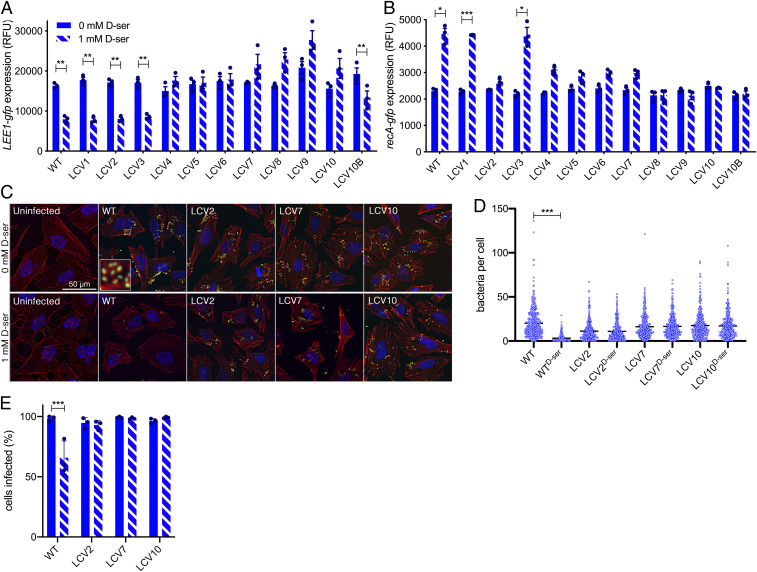
Tolerance to d-Ser has variable consequences for d-Ser–associated behaviors including type 3 secretion-dependent colonization. (*A*) *LEE1p* activity in the WT and the indicated mutants after 4 h of culture in MEM-HEPES ± 1 mM d-Ser. (*B*) *PrecA* activity in the WT and the indicated mutants after a 3-h culture in M9 + Glc followed by 2 h ± 1 mM d-Ser. *P < 0.05, **P < 0.01, and ***P < 0.001 by paired two-tailed Student’s *t* test. (*C*–*E*) Colonization of HeLa cells as determined by counting the numbers of EHEC attached per cell after 2 h 45 m of infection. (*C*) Deconvoluted wide-field microscopy images illustrating DNA (blue), filamentous actin (red), and EHEC (green) with bacteria being associated with regions of condensed actin, highlighted by a high-magnification image (*Inset*) for the WT. (*D*) Individual counts of bacteria per HeLa cell. A minimum of 331 cells were counted across three experiments. Black lines indicate the mean. Kruskal–Wallis with Dunn’s post hoc test yielded ***P < 0.001. (*E*) Percentage of HeLa infected with at least one EHEC cell obtained from cell counts in *D*. Ordinary one-way ANOVA of experimental means yielded ***P < 0.001.

Before characterizing the molecular basis of d-Ser tolerance, it was crucial to establish whether this tolerance phenotype had any implications for the distinct transcriptional responses that we have previously associated with exposure of EHEC to d-Ser, namely repression of type 3 secretion-mediated colonization (10) and activation of an SOS-like response via RecA induction (10, 22). We used transcriptional reporters of the LEE master regulator (Fig. 3*A*) and *recA* (Fig. 3*B*). As LCV1 and LCV3 did not exhibit tolerance, one would expect them to behave as the WT with respect to both *LEE1* repression and *recA* induction. This was indeed the case, further supporting the notion that these are false-positive isolates for d-Ser tolerance. The majority of d-Ser–tolerant isolates showed no repression of *LEE1* in response to d-Ser (LCV4 to 10) and no significant activation of *recA* transcription. This group consists of both partially tolerant and completely tolerant isolates (Fig. 2 *C* and *D*). Two isolates exhibited recovery from *recA* activation but maintained WT levels of type 3 repression (LCV2 and LCV10B; Fig. 3 *A* and *B*).

Next, a single representative from these three groupings (no *recA* induction, complete tolerance: LCV2; no *recA* induction or type 3 repression with partial tolerance: LCV7; no *recA* induction or type 3 repression with complete tolerance: LCV10) was tested for infection of host epithelial cells in the presence or absence of d-Ser (Fig. 3 *C*–*E*). Adherent bacteria formed characteristic A/E lesions with regions of pronounced actin condensation being visible adjacent to the majority of bacterial cells (Fig. 3*C*). Only WT EHEC showed a significant reduction in adhesion levels in terms of the numbers of bacteria attached per cell (Fig. 3*D*) and the percentage of cells infected (Fig. 3*E*). Surprisingly, LCV2 exhibited reduced *LEE1* promoter activity in the presence of d-Ser while displaying higher levels of colonization (Fig. 3*A*); however, we will later discuss further a mechanism by which LCV2 and LCV10B may gain an advantage in the presence of d-Ser. Taken together, these findings illustrate that tolerance starkly affects the transcriptional response to d-Ser and results in overcoming the inhibitory effects of d-Ser on colonization, a factor that could have important implications for niche specificity of such mutants.

### The Molecular Basis of d-Ser Tolerance.

Having observed variable levels of tolerance to d-Ser and distinct patterns of recovery from the transcriptional shift normally associated with d-Ser, we predicted that three distinct mechanisms of tolerance were at play. First, the genomes of each isolate were sequenced to 30-fold coverage and aligned to a resequenced genome of the EHEC parental strain from this study. Single-nucleotide polymorphisms (SNPs) were called where present in 90% of reads. Of the nonsynonymous SNPs present in tolerant isolates, only those in *cycA* appeared to be strongly selected for, being present in both experimental replicates (i.e., LCV4 to 10 [premature stop at position K^280^] and LCV1A [I^257^K]; Table 1), appearing early (batch cultures 4 and 1, respectively), and being maintained in the 10-d experiment until the final day of sampling. CycA is a previously characterized d-Ser/d-cycloserine/d-alanine/glycine/l-alanine transporter (18, 19). While the *cycA* mutation alone could putatively be responsible for the tolerance phenotype of the majority of the tolerant mutants (LCV4 to 10 and 1A), two questions remained: Why were some *cycA* mutants more tolerant than others, and what was the mechanism of tolerance for LCV2 and LCV10B that appear to encode WT *cycA*?

**Table 1.** Mutations detected in d-serine–adapted EHEC isolates

Gene/region mutated	Mutation	Alteration in amino acid sequence^†^	Annotation	Isolate(s) possessing mutation
*cycA*	MNP	I^257^K	Transporter	1A
*tamB*	SNP	S^358^R	Membrane assembly	1A
*ygfL*	SNP	I^384^V	Enzyme	3
*cycA*	SNP	K^280^*	Transporter	4 to 10
*z1097*	INS	NNK^411^NNNK*I	Hypothetical	9
*z2148*	SNP	R^52^G	Phage tail	10 and 10B
*z1364*	SNP	None	Phage portal	1 and 2
*z1364*	SNP	None	Phage portal	2
*yqhD*	SNP	None	Enzyme	3
*z2148*	SNP	None	Phage tail	10B
*z2148*	SNP	None	Phage tail	10B

INS, insertion; MNP, multiple-nucleotide polymorphism.

^†^ Stop codon (*).

**Fig. 4.**
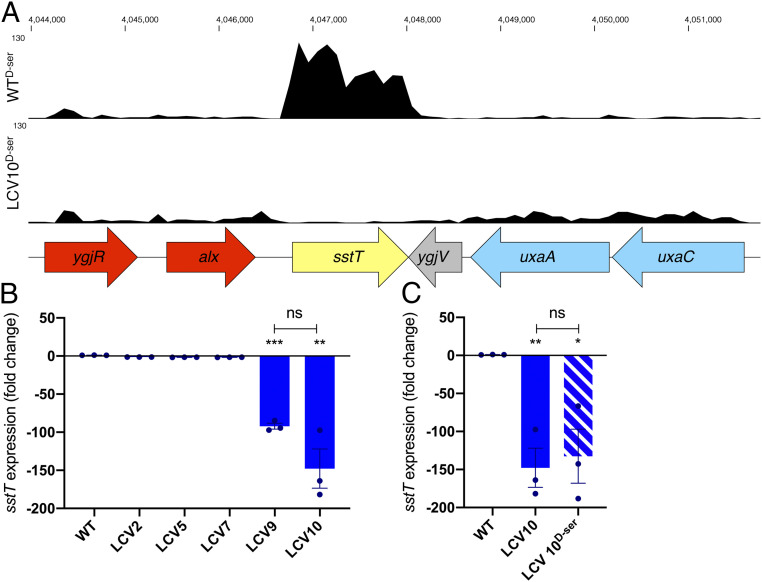
Constitutive knockdown in the serine transporter SstT occurs in isolates that possess a premature stop codon in *cycA* and display complete d-Ser tolerance. (*A*) Representative RNA-sequencing (RNA-seq) tracks of WT and LCV10 cultured for 5 h in MEM-HEPES + 1 mM d-Ser together with a schematic illustrating the gene organization within this locus. (*B* and *C*) Quantitative real-time PCR of *sstT* transcription following 5 h of growth in M9 + Glc ± 1 mM d-Ser. Triplicate experimental values for each isolate were compared with the WT with *P < 0.05, **P < 0.01, and ***P < 0.001 by unpaired two-tailed Student’s *t* test. ns, not significant.

To address the first question, we examined the transcriptome of a representative from the completely tolerant group that possessed a mutation in *cycA*—LCV10—and compared it with the WT. We hypothesized that a transcriptional alteration in this strain might explain its enhanced tolerance over the partially tolerant group (LCV4 to 7 and 1A). Wild-type EHEC was found to significantly differentially express 369 genes in response to d-Ser (136 up and 233 down; *SI Appendix*, Table S1), consistent with our previous reports (10). The majority (247/369) of these differentially expressed genes (DEGs) were oppositely regulated when comparing the transcriptome of WT^d-Ser^ and LCV10^d-Ser^ (*SI Appendix*, Table S1), highlighting the fact that tolerance can overcome many of the transcriptional effects exerted by d-Ser. A further 361 genes (new DEGs; *SI Appendix*, Table S2) were differentially expressed in LCV10^d-Ser^ versus WT^d-Ser^ that were not associated with the response to d-Ser (i.e., WT versus WT^d-Ser^), indicating that the transcriptional alterations in LCV10 extended beyond simply overcoming the normal effects of d-Ser. The most significant of these was *sstT* (also known as *yjgU*) with 87.68-fold lower expression (false discovery rate adjusted *P* = 1.86 × 10^−107^) in LCV10 than the WT (Fig. 4*A*). Reduced transcription of *sstT* was confirmed by quantitative real-time PCR (LCV10, −147.74-fold; Fig. 4*B*) and was also observed in a second completely tolerant isolate (LCV9, −92.15-fold), while two partially tolerant isolates (LCV5 and LCV7) and one tolerant isolate that lacked a mutation in *cycA* (LCV2) showed less than a twofold decrease in transcription. Decreased expression of *sstT* was found to be constitutive with no significant difference being observed with or without d-Ser (Fig. 4*C*). SstT functions as a sodium/serine and sodium/threonine symporter (20, 25), and hence transcriptional knockdown had a strong likelihood of facilitating tolerance synergistically with disruption of *cycA*.

### Disruption of d-Ser Transport as a Means of Conferring Tolerance.

**Fig. 5.**
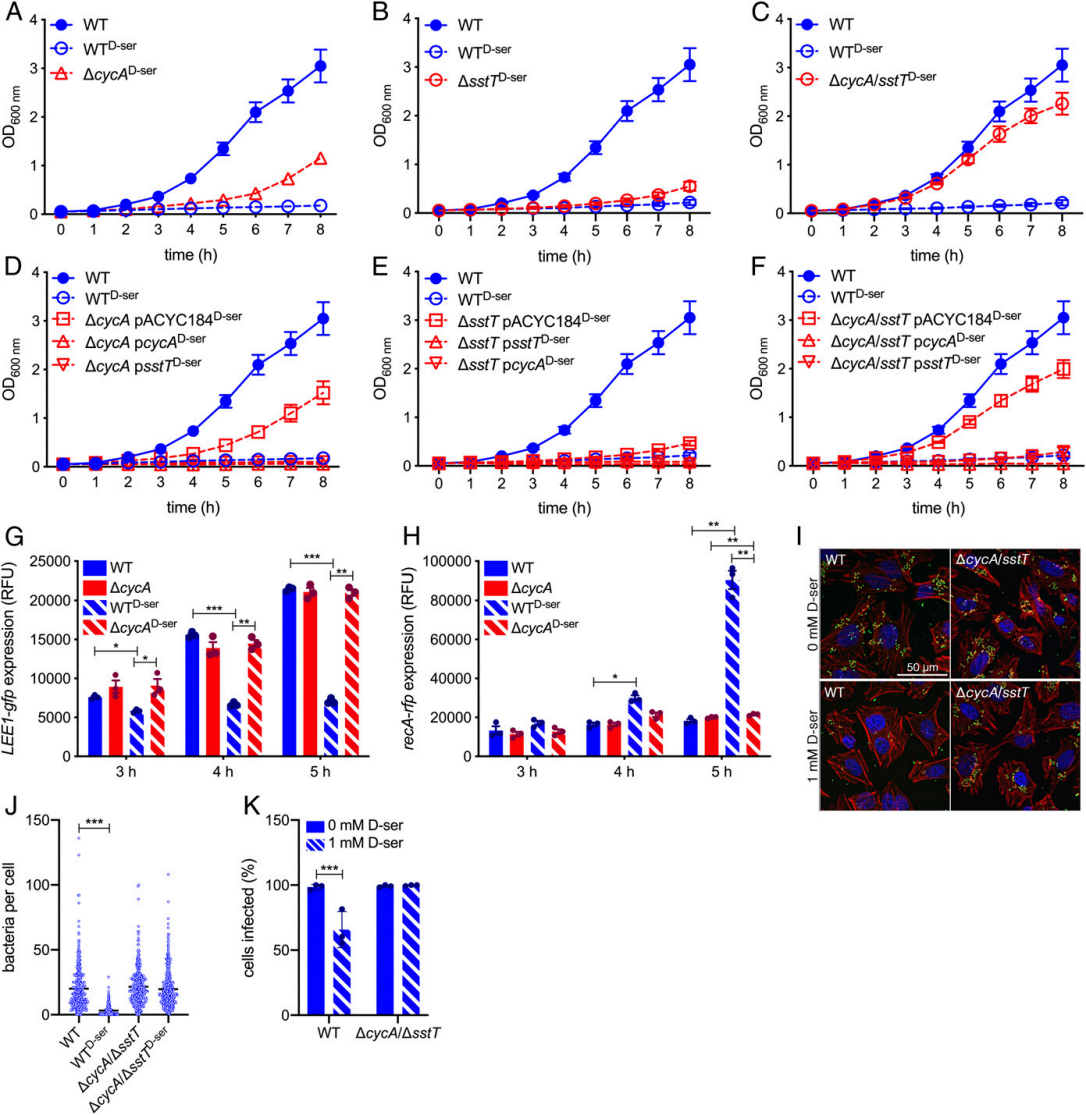
Deletion of serine transporters CycA and SstT reproduces phenotypes of tolerant isolates. (*A*–*F*) Growth curves of the WT, *cycA*, *sstT*, and *cycA*/*sstT* double mutant, and corresponding complementation strains in M9 + Glc ± 1 mM d-Ser. Error bars indicate SEM with a minimum of three experiments being performed. (*G* and *H*) Dual *LEE1*-*gfp*/*recA*-*rfp* promoter fusion reporter analysis of the WT and *cycA* mutants cultured in MEM-HEPES at the indicated time points. Three experiments were performed, with error bars depicting SEM. *P < 0.05, **P < 0.01, and ***P < 0.001 by paired two-tailed Student’s *t* test. (*I*–*K*) Colonization of HeLa cells as determined by counting the numbers of EHEC attached per cell after 2 h 45 m of infection. (*I*) Deconvoluted wide-field microscopy images illustrating DNA (blue), filamentous actin (red), and EHEC (green) with bacteria being associated with regions of condensed actin. (*J*) Individual counts of bacteria per HeLa cell. A minimum of 350 cells were counted across three experiments. Black lines indicate the mean. Kruskal–Wallis with Dunn’s post hoc test yielded ***P < 0.001. (*K*) Percentage of HeLa infected with at least one EHEC cell. Ordinary one-way ANOVA of experimental means yielded ***P < 0.001.

We hypothesized that the mutations acquired in CycA (K^280^Stop, I^257^K) and the marked reduction in *sstT* transcription led to lack of function or expression at a level that would not allow for functionality, thereby facilitating the observed phenotypes in LCV4 to 10 and 1A. To confirm these mechanisms, we constructed isogenic mutants lacking either CycA, SstT, or both transporters and assayed for tolerance to d-Ser. Deletion of *cycA* led to enhanced growth in the presence of d-Ser (Fig. 5*A*), with SGR increasing from 0.15 to 0.35 h^−1^ (*SI Appendix*, Fig. S3). While deletion of *sstT* caused only a modest increase in tolerance (Fig. 5*B*), the increase in SGR over WT^d-Ser^ was not significant (*P* = 0.122; *SI Appendix*, Fig. S3). Combined disruption of *cycA* and *sstT* resulted in complete tolerance to d-Ser (Fig. 5*C*) with a SGR of 0.61 h^−1^, statistically equivalent to that of WT^d-Ser^ (*SI Appendix*, Fig. S3). Therefore, disruption of *cycA* alone recapitulates the phenotypes of LCV4 to 7 and 1A, while secondary disruption of *sstT* in this genetic background recapitulates the phenotype of LCV8 to 10. Sensitivity to d-Ser was restored in all three deletion mutants by overexpression of either transporter on the multicopy plasmid pACYC184 (Fig. 5 *D*–*F*). Interestingly, overexpression of CycA led to a hypersensitive phenotype with d-Ser inducing a killing effect, as revealed by negative SGR values being observed in all three mutant backgrounds (Fig. 5 *D*–*F* and *SI Appendix*, Fig. S3).

We next tested the Δ*cycA* and Δ*sstT* mutants for transcriptional response to d-Ser using a dual *LEE1*-*gfp* and *recA*-*rfp* reporter system. Similar to LCV4 to 10 and 1A, mutants lacking *cycA* no longer showed repression of *LEE1* (Fig. 5*G*) or activation of *recA* (Fig. 5*H*) after 4 h of growth. A 4.97-fold higher level of *recA* expression was seen with WT^d-Ser^ versus WT (*P* = 0.003) at 5 h, while d-Ser caused only a 1.07-fold induction of *recA* (*P* = 0.006) in the Δ*cycA* background. Transport of d-Ser via CycA appeared to be the predominant factor in determining transcriptional response as deletion of *sstT* provided no relief from d-Ser–induced type 3 repression (*SI Appendix*, Fig. S4*A*) or *recA* activation (*SI Appendix*, Fig. S4*B*). As seen with the corresponding LCV isolate (LCV10; Fig. 3 *C*–*E*), recovery from repression of *LEE1* (Fig. 5*G*) by disruption of d-Ser transport correlated with increased efficiency of colonization in a HeLa cell infection model (Fig. 5 *I*–*K*). Unlike WT EHEC, d-Ser no longer caused a reduction in visible A/E lesions (Fig. 5*I*), bacteria per cell (Fig. 5*J*), or percentage of cells infected (Fig. 5*K*). The data obtained from these isogenic transporter deletion mutants corroborate the results obtained with LCV4 to 10 and 1A and further support the idea that disruption of CycA and SstT can result in tolerance to d-Ser with implications for stress response and virulence of EHEC.

### A Transport-Independent Mechanism of Tolerance?

The data presented thus far suggest that disruption to serine transport systems can contribute to d-Ser tolerance; however, two tolerant isolates remained for which no transport-related SNPs were identified (LCV2 and LCV10B; Table 1). To investigate the underlying tolerance mechanism, we employed transcriptome profiling. Comparing WT^d-Ser^ and LCV2^d-Ser^ revealed 660 DEGs, 258 of which comprised genes differentially expressed upon WT treatment with d-Ser (*SI Appendix*, Table S3). Of these, 233 were oppositely regulated, illustrating that, similar to LCV10, LCV2 had overcome the majority of transcriptional alterations exerted on WT EHEC by d-Ser. Comparing WT^d-Ser^ and LCV2^d-Ser^ also revealed 401 new DEGs not included in the WT/WT^d-Ser^ comparison, indicating that the transcriptional alterations in LCV2 extended beyond simply overcoming the effects of d-Ser. Among the most significant of these alterations were decreased expression of *cscR*/*z3626* (−147.74-fold, *P* = 6.50 × 10^−39^) and up-regulation of *cscA*/*z3625* (58.29-fold, *P* = 2.66 × 10^−122^), *cscK*/*z3624* (155.74-fold, *P* = 2.89 × 10^−89^), *cscB*/*z3623* (28.24-fold, *P* = 2.58 × 10^−67^), and *dsdA* (33.05-fold, *P* = 1.34 × 10^−108^).

**Fig. 6.**
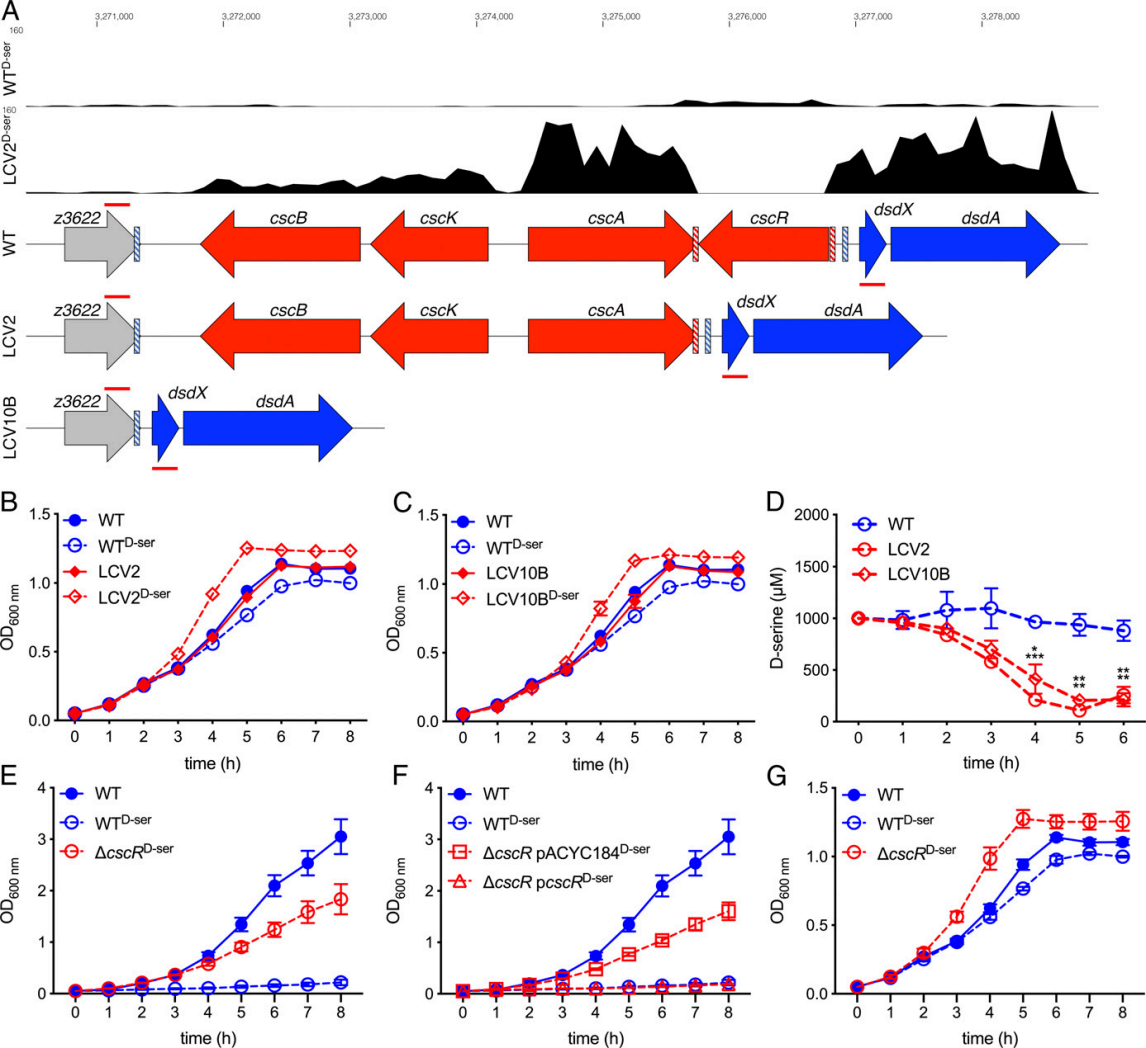
Genomic rearrangements upstream of the inactive EHEC *dsd* locus result in activation of d-Ser catabolism, thereby allowing tolerance via a distinct mechanism. (*A*) Representative RNA-seq tracks of the WT and LCV2 cultured in MEM-HEPES d-Ser for 5 h. Gene tracks indicate WT, LCV2, and LCV10B organization. Red/blue dashed boxes indicate direct repeats identified flanking the deletion in LCV2/10B, respectively. Annealing sites for primers used to screen for deletions in this region are indicated by red lines above and below each gene track. (*B* and *C*) Growth curves of LCV2 and LCV10B cultured in MEM-HEPES ± 1 mM d-Ser. Error bars indicate SEM with a minimum of three experiments being performed. (*D*) Concentration of d-Ser in cell-free supernatant from MEM-HEPES cultures as determined by d-Ser assay. The experiment was performed in triplicate. Error bars indicate SEM. *P < 0.05, **P < 0.01, and ***P < 0.001 by unpaired two-tailed Student’s *t* test. (*E* and *F*) Growth curves of the WT, Δ*cscR* (*E*), and *cscR* complementation strains (*F*) cultured in M9 + Glc ± 1 mM d-Ser. (*G*) Growth curves of the WT and Δ*cscR* cultured in MEM-HEPES ± 1 mM d-Ser. Error bars indicate SEM with a minimum of three experiments being performed (*E*–*G*).

The first of these genes (*cscR*) was particularly interesting as no reads were obtained for LCV2^d-Ser^ (Fig. 6*A*), accounting for the apparent large degree of differential expression. We hypothesized that a large genomic rearrangement may have occurred within this region that was not detected by our SNP-calling pipeline. Manual assessment of genome sequencing from this region in LCV2 and LCV10B showed a striking lack of coverage, suggesting that genomic reorganization had led to a loss of genomic content (*SI Appendix*, Fig. S5*A*). Primers flanking the putative deletion site of LCV10B (annealing sites indicated by red lines in Fig. 6*A*) were used to screen all LCVs for similar deletions. Only LCV2 and LCV10B were found to have truncations in this region (*SI Appendix*, Fig. S5*B*). Sanger sequencing of the PCR amplicons from LCV2 and LCV10B confirmed the precise coordinates of the deletions. LCV2 possessed a 1,120-bp deletion spanning from 149 bp before the 3′ end of *cscA* to 8 bp downstream of the 5′ end of *cscR* (Fig. 6*A*) with a 2-bp direct repeat of CG flanking the deletion site (red dashed boxes in Fig. 6*A*). LCV10B possessed a 5,637-bp deletion spanning from 32 bp before the 3′ end of *z3622* to 122 bp downstream of the 5′ end of *cscR* (Fig. 6*A*) with an 8-bp direct repeat of CCGGATAA flanking the deletion site (blue dashed boxes in Fig. 6*A*). CscR represses the transcription of the sucrose utilization locus encoded by *cscBKA* (17). As the deletions in LCV2 and LCV10B resulted in loss of the 3′ end of *cscA* and the entire *csc* locus, respectively, these isolates lost their ability to grow on sucrose as a sole carbon source (*SI Appendix*, Fig. S5*C*).

As mentioned previously, loss of transcription of *cscR* in LCV2 coincided with increased transcription of not only *cscBKA* via derepression but also increased transcription of the d-Ser deaminase-encoding gene *dsdA* (Fig. 6*A* and *SI Appendix*, Table S3). This indicated that LCV2 and LCV10B may have gained an ability to catabolize d-Ser. Both LCV2^d-Ser^ and LCV10B^d-Ser^ exhibited enhanced growth (SGR, 0.53 and 0.53 h^−1^) compared with culture in minimal essential media-4-(2-hydroxyethyl)-1-piperazineethanesulfonic acid (MEM-HEPES) without d-Ser (0.40 and 0.41 h^−1^), whereas WT EHEC (0.41 h^−1^) displayed reduced growth upon inclusion of d-Ser (0.37 h^−1^; Fig. 6 *B* and *C* and *SI Appendix*, Fig. S6*A*). Both LCV2^d-Ser^ and LCV10B^d-Ser^ also reached a higher maximal OD_600__nm_ than WT^d-Ser^, while no difference was observed when d-Ser was absent (Fig. 6 *B* and *C* and *SI Appendix*, Fig. S6*B*), indicating that d-Ser contributed to increased biomass accumulation in LCV2 and 10B. Quantitation of d-Ser concentrations in this medium confirmed that enhanced growth of these strains correlated with the consumption of d-Ser with significantly lower levels of d-Ser being present in cell-free supernatants from LCV2 and LCV10B at 4, 5, and 6 h compared with WT EHEC (Fig. 6*D*). An isogenic *cscR* deletion mutant recapitulated the tolerance phenotype of LCV2 and LCV10B in M9 medium (Fig. 6*E*) and this phenotype was successfully complemented by reintroduction of *cscR* in *trans* (Fig. 6*F* and *SI Appendix*, Fig. S7). The Δ*cscR* mutant also displayed enhanced growth rate and maximal OD_600__nm_ in MEM-HEPES + d-Ser in support of catabolism and assimilation into biomass (Fig. 6*G*).

### Reactivation of *dsdA* via Tolerance-Mediated Adaptive Mutation.

There were two plausible explanations for the activation of the previously nonfunctional d-Ser deaminase gene *dsdA* by upstream genomic deletions. One possibility was that deletion of the transcriptional repressor CscR led to direct derepression of *dsdA* transcription via its own promoter, *dsdAp*. The second explanation was that the alteration in genomic context of *dsdA* could lead to control via read-through from alternative promoters such as *cscAp* in the case of LCV2 or *z3622p* in the case of LCV10B.

To explore the first possibility, we examined the effect of heterologous CscR expression in UPEC. UPEC lacks the *csc* sucrose utilization locus and, as previously mentioned, possesses a functional *dsd* system for catabolism of d-Ser. The promoter element of *dsdA* is located 115 bp upstream of the start codon; 114 bp of this sequence are conserved between EHEC and UPEC and, as such, it could be expected that any putative CscR binding in this region would also be conserved. Expression of CscR in UPEC did not result in a decrease in tolerance to d-Ser in M9 minimal medium (*SI Appendix*, Fig. S8*A*), as carriage of empty pACYC184 and p*cscR* resulted in similar rates of growth. Qualitative real-time PCR demonstrated that transcription of UPEC *dsdCXA* occurred at a low steady-state level but increased markedly within 5 min postaddition of d-Ser and remained at a steady state for at least 20 min (*SI Appendix*, Fig. S8*B*). Real-time PCR at 0, 10, and 20 min and quantitative real-time PCR at 0 and 20 min revealed that expression of CscR did not reduce the steady-state level of *dsdCXA* transcription and did not affect the ability of *dsdCXA* to respond to d-Ser (*SI Appendix*, Fig. S8 *B*–*E*). It therefore appeared that CscR was not a direct repressor of d-Ser catabolism.

**Fig. 7.**
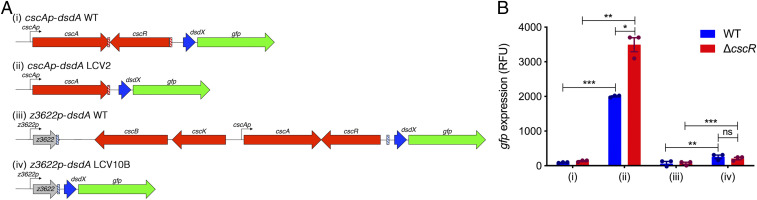
Deletions upstream of *dsd* enhance *dsdA* transcription due to control via alternative promoters. (*A*) Schematic representation of the genetic fusions prepared from the WT, LCV2, and LCV10B to examine the effect of altered genomic context on expression of *dsdA*. (*B*) Reporter assay carried out using *gfp* fusion constructs (*i*–*iv*, *A*). Gene regions were amplified from the indicated strains, cloned upstream of *gfp*, and transformed into the WT and Δ*cscR*. Fluorescence was recorded after a 4-h culture in MEM-HEPES with GFP production serving as a proxy for *dsdA* transcription. Triplicate experiments were performed. Error bars indicate SEM. *P < 0.05, **P < 0.01, and ***P < 0.001 by paired two-tailed Student’s *t* test.

The activation of *dsdA* by a new upstream regulatory element seemed more plausible given the data presented in Fig. 6*A*. The transcript read density for *dsdA* appears to correlate well with that of *cscA*, supporting the assertion that *dsdA* transcription in this strain may be driven by *cscAp*. The deletion in LCV2 was particularly serendipitous as it not only allowed for control via increasing the proximity of *cscAp* to *dsdA* but also via derepression of *cscAp* due to loss of CscR. To further test how the genomic organization of these regions affected *dsdA* expression, the relevant chromosomal regions (from *cscAp* or *z3622p* to the sixth codon of *dsdA*) of WT, LCV2, or LCV10B were fused to *gfp* (Fig. 7*A*) in a reporter plasmid such that green fluorescent protein (GFP) production could be used as a proxy for DsdA expression. As expected, the fragments that comprised *gfp* under the control of the canonical DsdA control elements (Fig. 7, fragments i and iii) produced low levels of fluorescence (63 to 134 relative fluorescence units; RFUs) and behaved similarly. Fragments ii and iv that were amplified from LCV2 and 10B and comprised *gfp* that was predicted to be under the control of *cscAp* and *z3622p*, respectively, produced fluorescence significantly higher (2,001 and 257 RFUs) than that of the WT amplified fragments, reflecting the constitutive activation of DsdA in these genetic contexts. Of particular interest was the fact that GFP production from fragment ii was significantly higher in the Δ*cscR* mutant than the WT whereas no significant difference between WT and mutant was observed with fragment iv. This illustrates that DsdA expression in LCV2 is further facilitated by derepression of *cscAp* due to lack of CscR, whereas expression via *z3622p* in LCV10B is not affected by CscR. This also explains why complementation of the Δ*cscR* mutant tolerance phenotype (Fig. 6*F*) is possible despite the fact that the change in chromosomal context is the principal driver of DsdA activity (i.e., overexpression of *cscR* in *trans* is capable of repressing *dsdA* in the Δ*cscR* mutant through interaction with chromosomal *cscAp*).

## Discussion

There is a growing appreciation for the role that the metabolic state of the gut plays in determining the behavior of both pathogenic and commensal bacteria. In particular, diet plays a vital role in determining health and disease outcomes, not only through manipulation of the nutritional balance of the host but also through modulation of bacterial species composition and gene expression (26, 27). It has been recently demonstrated that the use of trehalose as a food additive has led to the evolution of new lineages of *Clostridium difficile* with enhanced virulence and metabolic capacity (28), highlighting the importance of understanding how bacterial adaptation to dietary components can influence disease outcomes.

Many studies have pointed toward important roles for distinct dietary constituents in manipulating virulence and the progression of disease by A/E pathogens. Dietary fiber has been directly implicated in affecting disease severity in EHEC (29), while supplementation of diet with phytonutrients (plant extracts) (30) and pectin (31) reduce the severity of infection by *Citrobacter rodentium*—a natural pathogen of mice that employs a LEE-encoded type 3 secretion system similar to EHEC for intestinal colonization. Fucose has also been shown to repress expression of the LEE in EHEC via the fucose two-component sensor FusKR (32); however, the main source of free fucose is thought to be cleavage of intestinal mucins by commensal bacteria, while the contribution of diet to free fucose levels in the gut has been poorly studied (33). Similarly, biotin was shown to repress the LEE via the biotin sensor BirA which represses transcription of the global transcriptional regulator Fur, preventing it from activating the LEE (34). Accordingly, mice fed a high-biotin diet showed lower levels of intestinal colonization.

Of particular relevance to this study, catabolism of l-serine was recently shown to confer a competitive advantage to adherent invasive *E. coli* (AIEC) under chemically induced murine colitis and also to *C. rodentium* that can naturally induce colitis in mice (35). Pathogenic AIEC were better able to outcompete the nonpathogenic strain MG1655 when germfree mice were provided with a diet containing l-serine compared with a serine-deficient diet. Although l-serine does not influence expression of type 3 secretion genes in EHEC (10), this study raises the possibility that catabolism of both d- and l-serine may provide a competitive advantage to pathogenic *E. coli* during infection. Adaptation to d-Ser in this study resulted in two clones (LCV2 and LCV10B) gaining the ability to catabolize d-Ser, thereby not only alleviating toxicity as observed by overcoming growth inhibition and SOS induction but also expanding the available carbon and nitrogen source repertoire of the organism. The resulting enhanced growth rate of these isolates in d-Ser (Fig. 6 *B* and *C*) contributes to the higher numbers of colonizing bacteria observed with LCV2^d-Ser^ compared with WT^d-Ser^ (Fig. 3*D*) despite LEE repression being retained (Fig. 3*A*). In an in vivo context, this could potentially increase metabolic fitness in otherwise unfavorable environments.

The identification of genetically distinct mutants after 10 successive batch cultures is not surprising and is likely reflective of the fact that there are multiple routes to tolerance. Laboratory-based evolution experiments have been reported to result in convergent growth phenotypes via distinct modifications in gene expression states (36). Enteric species often partake in diverse adaptive strategies with genotypic and phenotypic heterogeneity allowing for enhanced versatility in the face of stress and competition (37, 38). In the complex and dynamic environment of the gut, mutations may be advantageous or deleterious depending on specific conditions. As such, heterogeneity represents a savvy route to establishment or maintenance of a successful niche. It is possible that there are further diverse mechanisms by which d-Ser tolerance can arise, each with varying implications for the expression of virulence and metabolism genes. Deep sequencing of a long-term adapted batch culture would yield interesting insight into potential heterogeneity of adaptation within a given population.

While the complete tolerance of LCV8 to 10 can be explained by reduced expression of SstT in conjunction with expression of a mutated CycA, the reason for this reduced rate of transcription is unclear. There appears to be an accumulation of antisense reads in the 5′ untranslated region of *sstT*, so it is tempting to speculate that control via a regulatory RNA may be at play. Both *sstT* (39) and *cycA* (40) have been shown to be regulated by the small RNA GcvB; however, no change in *gcvB* transcription was observed, nor were mutations in the GcvB binding site of *sstT* in LCV8 to 10 detected (Table 1). It should be noted that WT EHEC responds to d-Ser by reducing transcription of *cycA* (−2.38-fold, *P* = 4.09 × 10^−4^). As such, EHEC may respond to d-Ser by decreasing the transcription of d-Ser transporters; however, the precise regulatory framework through which constitutive knockdown of *sstT* is achieved remains elusive.

The mechanism by which d-Ser exerts its toxic growth-inhibitory effect on catabolically inactive *E. coli* has been the subject of some debate. Using a mutant of *E. coli* K-12 unable to produce d-Ser deaminase, it was shown that addition of l-serine and pantothenate could rescue the tolerance phenotype, thereby implicating a block in l-serine biosynthesis or pantothenate synthesis in d-Ser–induced toxicity (41). This may also explain why d-Ser does not induce growth arrest in more complex media such as MEM-HEPES (Fig. 5*B*). d-amino acids can be misincorporated into the peptidoglycan of gram-negative bacteria in place of the canonical cell-wall constituent d-amino acids (9, 42). We have previously speculated that the mechanism of d-Ser toxicity may involve a disruption to the peptidoglycan-remodeling machinery that is essential for growth, due to a modified composition of the muropeptide chains (10). The results of this study indicate that regardless of the mechanism of toxicity, disruption of d-Ser transport into the cytosol is sufficient to abrogate the inhibition of growth. Analysis of 115 *E. coli* O157:H7 genomes (National Center for Biotechnology Information BLASTp) reveals strict conservation (>99%) at the amino acid level for CycA and SstT, indicating strong selective pressure. CycA and SstT possess multiple amino acid substrates including alanine, threonine, and glycine. Disruption of transport of these molecules may compromise fitness in certain environments by reducing the efficacy of transport and catabolism of specific carbon and nitrogen sources. The deletions observed upstream of *dsdA* in this study result in a similar evolutionary tradeoff with such mutants losing the ability to metabolize sucrose. It will be important to analyze the effect of such alterations in the context of an in vivo infection.

Laboratory-based evolution studies have yielded valuable insight into how *E. coli* adapts to stress (43–45) and can acquire the ability to derive nutrition from noncanonical carbon sources (46, 47). In this study, we have described how multiple genetic mechanisms converge in surmounting d-Ser–induced stress in EHEC with each mechanism having implications for important pleiotropic behaviors including virulence, stress response, and catabolic capacity. We previously showed that the sensing of d-Ser and concordant repression of type 3 secretion-dependent colonization restrict EHEC to the gut (10). The present study aimed to investigate how EHEC might overcome this evolutionary bottleneck. The growth inhibition associated with d-Ser is not linked to type 3 repression as shown by our previous work. The data presented here indicate that through genomic plasticity, EHEC can overcome both the repression of colonization and growth-inhibitory effects of d-Ser. Hence, these adaptations could expand the niche specificity of EHEC, enabling infection in extraintestinal sites such as the bladder, thereby challenging the dogma that EHEC are strictly intestinal pathogens (10, 15). Caution should be exercised with this assertion as such mutations have yet to appear in the database. This could indicate that environmental substrate sampling via CycA acts as a critical regulator of niche restriction and loss of functionality could lead to a compromise in pathogen success. However, there have been recent reports of EHEC/UPEC hybrid strains (48, 49) with the ability to cause both intestinal and extraintestinal disease, suggesting that adaptation to alternative niches may already be occurring. Understanding the in vivo ecological implications of the adaptive mechanisms described here will form the basis of our future work.

## Materials and Methods

A complete list of bacterial strains, plasmids, and oligonucleotides used in this study can be found in *SI Appendix*, Tables S4–S6. A detailed description of all methodology is included in *SI Appendix*, *Materials and Methods*. This includes isolation of d-Ser–tolerant mutants, engineering of strains and plasmids, HeLa cell infections, DNA and RNA extraction, library preparation and sequencing, quantitative real-time PCR, d-Ser assay, and all data analysis tools.

## Supplementary Material

Supplementary File

## Data Availability

Raw sequence data reported in this paper have been deposited in the European Nucleotide Archive under accession nos. ERS4281497 to ERS4281509 (genome sequencing) and ERS4281510 to ERS4281521 (RNA sequencing). All remaining data are presented in the paper and *SI Appendix*.

## References

[1] Marcone G. L., Rosini E., Crespi E., Pollegioni L., D-amino acids in foods. Appl. Microbiol. Biotechnol. 104, 555–574 (2020). 31832715 10.1007/s00253-019-10264-9

[2] Metzler D. E., Snell E. E., Deamination of serine. II. D-serine dehydrase, a vitamin B6 enzyme from *Escherichia coli*. J. Biol. Chem. 198, 363–373 (1952). 12999751

[3] Nørregaard-Madsen M., McFall E., Valentin-Hansen P., Organization and transcriptional regulation of the *Escherichia coli* K-12 D-serine tolerance locus. J. Bacteriol. 177, 6456–6461 (1995). 7592420 10.1128/jb.177.22.6456-6461.1995 PMC177495

[4] Tanigawa M.., D-amino acid dehydrogenase from *Helicobacter pylori* NCTC 11637. Amino Acids 38, 247–255 (2010). 19212808 10.1007/s00726-009-0240-0

[5] Chang Y.-F., Adams E., D-lysine catabolic pathway in *Pseudomonas putida*: Interrelations with L-lysine catabolism. J. Bacteriol. 117, 753–764 (1974). 4359655 10.1128/jb.117.2.753-764.1974 PMC285570

[6] Pioli D., Venables W. A., Franklin F. C. H., D-alanine dehydrogenase. Its role in the utilisation of alanine isomers as growth substrates by *Pseudomonas aeruginosa* PA01. Arch. Microbiol. 110, 287–293 (1976). 13755 10.1007/BF00690240

[7] Kubota T., Kobayashi T., Nunoura T., Maruyama F., Deguchi S., Enantioselective utilization of D-amino acids by deep-sea microorganisms. Front. Microbiol. 7, 511 (2016). 27148200 10.3389/fmicb.2016.00511 PMC4836201

[8] Höltje J. V., Growth of the stress-bearing and shape-maintaining murein sacculus of *Escherichia coli*. Microbiol. Mol. Biol. Rev. 62, 181–203 (1998). 9529891 10.1128/mmbr.62.1.181-203.1998 PMC98910

[9] Lam H.., D-amino acids govern stationary phase cell wall remodeling in bacteria. Science 325, 1552–1555 (2009). 19762646 10.1126/science.1178123 PMC2759711

[10] Connolly J. P. R.., The host metabolite D-serine contributes to bacterial niche specificity through gene selection. ISME J. 9, 1039–1051 (2015). 25526369 10.1038/ismej.2014.242 PMC4366372

[11] Anfora A. T., Haugen B. J., Roesch P., Redford P., Welch R. A., Roles of serine accumulation and catabolism in the colonization of the murine urinary tract by *Escherichia coli* CFT073. Infect. Immun. 75, 5298–5304 (2007). 17785472 10.1128/IAI.00652-07 PMC2168303

[12] Anfora A. T., Halladin D. K., Haugen B. J., Welch R. A., Uropathogenic *Escherichia coli* CFT073 is adapted to acetatogenic growth but does not require acetate during murine urinary tract infection. Infect. Immun. 76, 5760–5767 (2008). 18838520 10.1128/IAI.00618-08 PMC2583553

[13] Kleckner N. W., Dingledine R., Requirement for glycine in activation of NMDA-receptors expressed in *Xenopus* oocytes. Science 241, 835–837 (1988). 2841759 10.1126/science.2841759

[14] Kaper J. B., Nataro J. P., Mobley H. L. T., Pathogenic *Escherichia coli*. Nat. Rev. Microbiol. 2, 123–140 (2004). 15040260 10.1038/nrmicro818

[15] Wong A. R. C.., Enteropathogenic and enterohaemorrhagic *Escherichia coli*: Even more subversive elements. Mol. Microbiol. 80, 1420–1438 (2011). 21488979 10.1111/j.1365-2958.2011.07661.x

[16] McDaniel T. K., Jarvis K. G., Donnenberg M. S., Kaper J. B., A genetic locus of enterocyte effacement conserved among diverse enterobacterial pathogens. Proc. Natl. Acad. Sci. 92, 1664–1668 (1995). 7878036 10.1073/pnas.92.5.1664 PMC42580

[17] Jahreis K.., Adaptation of sucrose metabolism in the *Escherichia coli* wild-type strain EC3132. J. Bacteriol. 184, 5307–5316 (2002). 12218016 10.1128/JB.184.19.5307-5316.2002 PMC135337

[18] Anfora A. T., Welch R. A., DsdX is the second D-serine transporter in uropathogenic *Escherichia coli* clinical isolate CFT073. J. Bacteriol. 188, 6622–6628 (2006). 16952954 10.1128/JB.00634-06 PMC1595467

[19] Cosloy S. D., D-serine transport system in *Escherichia coli* K-12. J. Bacteriol. 114, 679–684 (1973). 4574696 10.1128/jb.114.2.679-684.1973 PMC251826

[20] Ogawa W., Kim Y.-M., Mizushima T., Tsuchiya T., Cloning and expression of the gene for the Na^+^-coupled serine transporter from *Escherichia coli* and characteristics of the transporter. J. Bacteriol. 180, 6749–6752 (1998). 9852024 10.1128/jb.180.24.6749-6752.1998 PMC107783

[21] Connolly J. P. R.., A highly conserved bacterial D-serine uptake system links host metabolism and virulence. PLoS Pathog. 12, e1005359 (2016). 26727373 10.1371/journal.ppat.1005359 PMC4699771

[22] Connolly J. P. R., Roe A. J., Intracellular D-serine accumulation promotes genetic diversity via modulated induction of RecA in enterohemorrhagic *Escherichia coli*. J. Bacteriol. 198, 3318–3328 (2016). 27698085 10.1128/JB.00548-16 PMC5116935

[23] Moritz R. L., Welch R. A., The *Escherichia coli* argW-dsdCXA genetic island is highly variable, and *E. coli* K1 strains commonly possess two copies of dsdCXA. J. Clin. Microbiol. 44, 4038–4048 (2006). 17088369 10.1128/JCM.01172-06 PMC1698345

[24] Smirnova G. V., Oktyabrsky O. N., Relationship between *Escherichia coli* growth rate and bacterial susceptibility to ciprofloxacin. FEMS Microbiol. Lett. 365 (2018). 10.1093/femsle/fnx254 29228224

[25] Kim Y.-M.., Purification, reconstitution, and characterization of Na(+)/serine symporter, SstT, of *Escherichia coli*. J. Biochem. 132, 71–76 (2002). 12097162 10.1093/oxfordjournals.jbchem.a003201

[26] David L. A.., Diet rapidly and reproducibly alters the human gut microbiome. Nature 505, 559–563 (2014). 24336217 10.1038/nature12820 PMC3957428

[27] Kau A. L., Ahern P. P., Griffin N. W., Goodman A. L., Gordon J. I., Human nutrition, the gut microbiome and the immune system. Nature 474, 327–336 (2011). 21677749 10.1038/nature10213 PMC3298082

[28] Collins J.., Dietary trehalose enhances virulence of epidemic *Clostridium difficile*. Nature 553, 291–294 (2018). 29310122 10.1038/nature25178 PMC5984069

[29] Zumbrun S. D.., Dietary choice affects Shiga toxin-producing *Escherichia coli* (STEC) O157:H7 colonization and disease. Proc. Natl. Acad. Sci. 110, E2126–E2133 (2013). 23690602 10.1073/pnas.1222014110 PMC3677460

[30] Wlodarska M., Willing B. P., Bravo D. M., Finlay B. B., Phytonutrient diet supplementation promotes beneficial *Clostridia* species and intestinal mucus secretion resulting in protection against enteric infection. Sci. Rep. 5, 9253 (2015). 25787310 10.1038/srep09253 PMC4365398

[31] Jimenez A. G., Ellermann M., Abbott W., Sperandio V., Diet-derived galacturonic acid regulates virulence and intestinal colonization in enterohaemorrhagic *Escherichia coli* and *Citrobacter rodentium*. Nat. Microbiol. 5, 368–378 (2020). 31873206 10.1038/s41564-019-0641-0 PMC6992478

[32] Pacheco A. R.., Fucose sensing regulates bacterial intestinal colonization. Nature 492, 113–117 (2012). 23160491 10.1038/nature11623 PMC3518558

[33] Becker D. J., Lowe J. B., Fucose: Biosynthesis and biological function in mammals. Glycobiology 13, 41R–53R (2003). 10.1093/glycob/cwg054 12651883

[34] Yang B., Feng L., Wang F., Wang L., Enterohemorrhagic *Escherichia coli* senses low biotin status in the large intestine for colonization and infection. Nat. Commun. 6, 6592 (2015). 25791315 10.1038/ncomms7592 PMC4382993

[35] Kitamoto S.., Dietary L-serine confers a competitive fitness advantage to Enterobacteriaceae in the inflamed gut. Nat. Microbiol. 5, 116–125 (2020). 31686025 10.1038/s41564-019-0591-6 PMC6925351

[36] Fong S. S., Joyce A. R., Palsson B. Ø., Parallel adaptive evolution cultures of *Escherichia coli* lead to convergent growth phenotypes with different gene expression states. Genome Res. 15, 1365–1372 (2005). 16204189 10.1101/gr.3832305 PMC1240078

[37] Magdanova L. A., Golyasnaya N. V., Heterogeneity as an adaptive trait of microbial populations. Microbiology 82, 1–10 (2013). 10.7868/s0026365613010072 23718043

[38] Martins B. M. C., Locke J. C. W., Microbial individuality: How single-cell heterogeneity enables population level strategies. Curr. Opin. Microbiol. 24, 104–112 (2015). 25662921 10.1016/j.mib.2015.01.003

[39] Pulvermacher S. C., Stauffer L. T., Stauffer G. V., The small RNA GcvB regulates sstT mRNA expression in *Escherichia coli*. J. Bacteriol. 191, 238–248 (2009). 18952787 10.1128/JB.00915-08 PMC2612445

[40] Pulvermacher S. C., Stauffer L. T., Stauffer G. V., Role of the sRNA GcvB in regulation of *cycA* in *Escherichia coli*. Microbiology 155, 106–114 (2009). 19118351 10.1099/mic.0.023598-0

[41] Cosloy S. D., McFall E., Metabolism of D-serine in *Escherichia coli* K-12: Mechanism of growth inhibition. J. Bacteriol. 114, 685–694 (1973). 4574697 10.1128/jb.114.2.685-694.1973 PMC251827

[42] Cava F., de Pedro M. A., Lam H., Davis B. M., Waldor M. K., Distinct pathways for modification of the bacterial cell wall by non-canonical D-amino acids. EMBO J. 30, 3442–3453 (2011). 21792174 10.1038/emboj.2011.246 PMC3160665

[43] Goodarzi H.., Regulatory and metabolic rewiring during laboratory evolution of ethanol tolerance in *E. coli*. Mol. Syst. Biol. 6, 378 (2010). 20531407 10.1038/msb.2010.33 PMC2913397

[44] Rudolph B., Gebendorfer K. M., Buchner J., Winter J., Evolution of *Escherichia coli* for growth at high temperatures. J. Biol. Chem. 285, 19029–19034 (2010). 20406805 10.1074/jbc.M110.103374 PMC2885180

[45] Winkler J. D., Garcia C., Olson M., Callaway E., Kao K. C., Evolved osmotolerant *Escherichia coli* mutants frequently exhibit defective N-acetylglucosamine catabolism and point mutations in cell shape-regulating protein MreB. Appl. Environ. Microbiol. 80, 3729–3740 (2014). 24727267 10.1128/AEM.00499-14 PMC4054140

[46] Lee D.-H., Palsson B. Ø., Adaptive evolution of *Escherichia coli* K-12 MG1655 during growth on a nonnative carbon source, L-1,2-propanediol. Appl. Environ. Microbiol. 76, 4158–4168 (2010). 20435762 10.1128/AEM.00373-10 PMC2897412

[47] Gleizer S.., Conversion of *Escherichia coli* to generate all biomass carbon from CO_2_. Cell 179, 1255–1263.e12 (2019). 31778652 10.1016/j.cell.2019.11.009 PMC6904909

[48] Bielaszewska M.., Heteropathogenic virulence and phylogeny reveal phased pathogenic metamorphosis in *Escherichia coli* O2:H6. EMBO Mol. Med. 6, 347–357 (2014). 24413188 10.1002/emmm.201303133 PMC3958309

[49] Gati N. S., Middendorf-Bauchart B., Bletz S., Dobrindt U., Mellmann A., Origin and evolution of hybrid Shiga toxin-producing and uropathogenic *Escherichia coli* strains of sequence type 141. J. Clin. Microbiol. 58, e01309-19 (2019). 31619530 10.1128/JCM.01309-19 PMC6935910

