# Supplementary material for: Genomic plasticity of pathogenic Escherichia coli mediates d-serine tolerance via multiple adaptive mechanisms
Source: Proc Natl Acad Sci U S A. 2020 Aug 26;117(36):22484–93. doi: 10.1073/pnas.2004977117 (PMC7486766; doi:10.1073/pnas.2004977117)
Supplement: Supplementary File [file pnas.2004977117.sapp.pdf]

Supplementary Information Appendix

Genomic plasticity of pathogenic *Escherichia coli* mediates D-serine tolerance via multiple adaptive mechanisms

Nicky O'Boyle[a], James P.R. Connolly[ab], Nicholas R. Tucker[c] and Andrew J. Roe[a*]

This PDF includes

Materials and Methods

Supplementary Figures S1-S8

Supplementary Tables S1-S6

Supplementary References

Materials and Methods

Strains, plasmids and culture conditions

Bacterial strains, plasmids and oligonucleotides used in this study are listed in SI Appendix, Tables S4, S5 and S6 respectively. Bacteria were routinely cultured in LB with antibiotics where appropriate, overnight at 37°C before diluting 100-fold (approximate $OD_{600\ nm}$ = 0.05) into the appropriate medium for experiments/growth analysis. Chloramphenicol, kanamycin and ampicillin were used at 20, 40 and 100 µg ml^{-1} respectively. All preparations of M9 minimal medium (Sigma Aldrich; cat #M7278) were supplemented 0.4% (w/v) glucose unless otherwise stated. For RNA-seq and experiments requiring type 3 secretion system activity, MEM-HEPES (Sigma Aldrich; cat #M7278) was employed to induce expression. All growth media, antibiotics and chemicals were purchased from Sigma Aldrich unless otherwise stated.

Isolation of D-ser tolerant mutants

This procedure is summarized in the schematic Fig. 2A. For isolation of D-ser tolerant mutants, overnight LB culture of EHEC was centrifuged at 10,000 × *g* for two minutes and the resulting pellet washed three times with phosphate buffered saline (PBS) before resuspending in M9 + 1 mM D-ser, to remove any residual LB. A ten-milliliter volume of M9 + 1 mM D-ser was inoculated to an $OD_{600\ nm}$ of 0.05 and the bacteria were incubated at 37°C with shaking for 24 h. The $OD_{600\ nm}$ was recorded and an appropriate volume was added to a second ten-milliliter volume of M9 + 1 mM D-ser. This was incubated at 37°C with shaking for 24 h and the process repeated each day until the tenth batch culture had been reached. On each day, a sample of culture was serially diluted, and spot plated on solid M9 + 1 mM D-ser. After 24 h growth at 37°C,

a representative of the largest colony type (i.e. large colony variant: LCV) observed was streaked on LB for purity before stocking for subsequent phenotypic analysis and genome sequencing.

Cloning of dual fluorescence reporter plasmids

A promoterless *gfp*/*rfp* reporter plasmid (pDUAL) was prepared by amplifying promoterless *rfp* from pJ241and cloning into a PaeI/SalI digested fragment of pAJR70. The resulting plasmid allows for in-frame cloning of promoters to *gfp* using BamHI/KpnI and in-frame cloning of promoters to *rfp* using PagI/PaeI. A functional dual reporter plasmid (p*LEE1*-*gfp*+*recA*-*rfp*) was also constructed by simultaneously cloning a PagI/PaeI digested *recAp* fragment and a PaeI/SalI digested *rfp* fragment into a PagI/SalI digested p*LEE1*-*gfp*.

***In vitro* fluorescence based reporters of gene expression**

The plasmids p*LEE1*-*gfp* (1), p*recA*-*gfp* (2) and the dual reporter p*LEE1*-*gfp*+*recA*-*rfp* (SI Appendix, Table S5) were used to report the transcriptional activity of the LEE1 operon and the SOS mediator *recA* in the presence and absence of D-ser. The first gene of the LEE1 operon encodes the master regulator Ler, which converges on all five operons that comprise the LEE (3), therefore activity of *LEE1p* is an ideal reporter for type 3 secretion system activity. Similarly, RecA mediates the first step in the activation of the SOS response by proteolytically cleaving the transcriptional repressor LexA, thereby facilitating a key program of transcription that is normally associated with DNA repair (4). As such, *recAp* activity is an excellent indicator of SOS response induction. The plasmids were transformed into the appropriate strains by

electroporation. Overnight LB cultures were diluted in the appropriate medium and cultured at 37°C to the desired $OD_{600\ nm}$ (mid-late exponential phase). Optical density was recorded before transferring culture to black microtiter plates and recording fluorescence (GFP: 485 nm excitation, 520 nm emission; RFP: 544 nm excitation, 620 nm emission) in a FLUOstar Optima plate reader (BMG Labtech, UK). Fluorescence data was background corrected by subtracting fluorescence intensity of a strain carrying empty pAJR70 at each timepoint, before normalizing data by expressing background corrected fluorescence intensity relative to $OD_{600\ nm}$. Mean data from three experiments are presented with two-tailed Student's *t*-test being used to test for significant differences.

Colonization of HeLa epithelial cells

Overnight LB cultures of EHEC strains carrying p*rpsM-gfp* (for constitutive GFP expression; chloramphenicol selection was maintained in all growth media) were diluted in MEM-HEPES -/+ 1 mM D-ser and cultured to mid-exponential phase ($OD_{600\ nm}$ 0.5 - 0.7). Bacterial suspensions were diluted to $OD_{600\ nm}$ = 0.1, before serially diluting and spot plating on LB plates to confirm appropriate concentration. HeLa were routinely cultured in MEM-HEPES with 10% FCS. For infection experiments, 4×10^4 HeLa were seeded on 13 mm glass coverslips pre-coated with collagen (Millipore; cat# 08-115) as per the manufacturer's instructions. After 24 h and immediately prior to infection, the HeLa cells were washed with PBS and medium was replaced with MEM-HEPES -/+ 1 mM D-ser. Bacteria were added to each coverslip at a multiplicity of infection of 50 (20 µl of 0.1 $OD_{600\ nm}$ suspension) and infected for 2 h 45 min. The tissues were washed six times with PBS before fixing with 4% (w/v) paraformaldehyde.

The tissues were washed once and permeabilized with 0.1% (v/v) Triton X-100 in PBS for 5 min. After two additional washes, each coverslip was stained with 0.2 U Phalloidin-AlexaFluor 555 (Thermofisher Scientific). Tissues were washed three times with PBS before mounting on glass slides in 3 µl Vectashield with DAPI (Vector Laboratories) and sealing with nail polish. Widefield microscopy images were taken with a Zeiss Axioimager M1 and Zen Pro software. Type 3 secretion-mediated colonization was clearly evident by co-localization of condensed actin with adherent bacteria. Adherent bacteria per cell was enumerated from eight widefield images per strain in each experiment with the experiment being repeated in triplicate. As such, at least 331 host cells were counted for each strain. Data are presented as individual cell counts with mean bacteria per cell and percentage of cells infected with at least one bacterium. Two-tailed Student's *t*-test was performed on the experimental means to assess the significance of differences between strains.

Whole genome sequencing of D-ser tolerant mutants

Bead stocks were prepared from pure culture LB agar streaks of each isolate before sending to MicrobesNG (Birmingham, United Kingdom) for DNA extraction and sequencing as follows: Three beads were washed with extraction buffer containing lysozyme and RNase A and incubated for 25 min at 37°C. Proteinase K and RNaseA were added and incubated for 5 min at 65°C. Genomic DNA was purified using an equal volume of solid phase reversible immobilization beads and resuspended in elution buffer. DNA was quantified in triplicates with the Quant-iT dsDNA high sensitivity assay in an Eppendorf AF2200 plate reader. Genomic DNA libraries were prepared using Nextera XT Library Prep Kit (Illumina; San Diego, USA) following the

manufacturer's protocol with the following modifications: two nanograms of DNA instead of one were used as input, and PCR elongation time was increased to 1 min from 30 seconds. DNA quantification and library preparation were carried out on a Hamilton Microlab STAR automated liquid handling system. Pooled libraries were quantified using the Kapa Biosystems Library Quantification Kit for Illumina on a Roche light cycler 96 qPCR machine. Libraries were sequenced on the Illumina HiSeq using a 250bp paired end protocol. Reads were adapter trimmed using Trimmomatic 0.30 with a sliding window quality cutoff of Q15 (5). De novo assembly was performed on samples using SPAdes version 3.7 (6), and contigs were annotated using Prokka 1.11 (7). Genome sequencing data was submitted to the European Nucleotide Archive (ENA) under the accession numbers ERS4281497 to ERS4281509.

Single nucleotide polymorphism (SNP) assignment

Reads from each isolate were mapped to a TUV93-0 EHEC WT genome re-sequenced as part of this study using Snippy (8). SNPs present in 90% of reads, with a minimum quality score of 60 and minimum site coverage of 10 reads were considered. Mutations were also cross-checked against the reference EDL933 genome (NCBI accession number: NC_002655.2) before manually viewing in Artemis (9) and preparing the final list (Table 1).

RNA extraction

Bacteria were cultured in MEM-HEPES to late exponential phase (OD600 nm = 0.8). Cells were harvested by centrifugation before resuspension in two volumes of RNAprotect Bacteria Reagent (Qiagen). After 5 min incubation at room temperature,

cells were harvested, and lysis and RNA extractions were carried out using PureLink RNA Mini Kit (Thermofisher Scientific) according to manufacturer's instructions. Contaminating DNA was removed by TurboDNase (Thermofisher Scientific) treatment, followed by extraction in acid phenol chloroform (Thermofisher Scientific) for qRT-PCR or MEGAclear (Thermofisher Scientific) clean-up for RNA-seq.

Transcriptome profiling by RNA-seq

RNA quality was assessed by Agilent Bioanalyzer 2100. Samples were subjected to ribosomal depletion using MICROBExpress (Thermofisher Scientific) according to the manufacturer's instructions. Library preparation and sequencing was carried out at the University of Glasgow Polyomics facility. Sequencing libraries were prepared with the TrueSeq Stranded mRNA Library Prep kit (Illumina) according to manufacturer's instructions. Sequencing was carried out on the Illumina NextSeq 500 platform with at least 10 million 75 bp single end reads being obtained. Reads were quality assessed (minimum Phred threshold of 20) with FastQC (Babraham Bioinformatics) before importing into CLC Genomics Workbench (Qiagen) and mapping to the EDL933 reference genome (NCBI accession number: NC_002655.2) using default CLC mapping parameters. Differential expression was assessed using the empirical analysis of differential expression (EdgeR) with genes displaying absolute fold changes of ≥ 1.5; ≤ -1.5 and having a false-discovery rate corrected *p*-value of ≤ 0.05 being considered. Pairwise comparison of WT and $WT^{D\text{-}ser}$ was conducted to identify D-ser induced changes in gene expression. Pairwise comparison of $WT^{D\text{-}ser}$ and $LCV10^{D\text{-}ser}$ or $LCV2^{D\text{-}ser}$ was used to identify the extent of recovery from D-ser-associated changes in gene expression and cryptic underlying mechanisms of

tolerance. Track maps depicting read density were constructed from samples having similar total read counts and were normalized to the same peak read density before exporting from CLC Genomics Workbench. Raw data has been uploaded to the ENA under accession numbers ERS4281510 to ERS4281521.

Synthesis of cDNA and qRT-PCR

Ten nanograms of DNA-free total RNA, extracted as described above, was used as a template to prepare 10 µl cDNA using LunaScript RT SuperMix Kit (New England Biolabs) according to manufacturer's instructions. Luna qPCR Master mix (New England Biolabs) was employed for PCR according to manufacturer's recommendations (Initial denaturation 95°C, 1 min; denaturation 95°C, 15 sec; extension 60°C, 30 sec; 40 cycles). Technical duplicate 20 µl reactions were carried out with 1 µl volumes of cDNA as template and triplicate biological samples of cDNA being analyzed. Absolute fold change was calculated as $2^{-\Delta\Delta ct}$ with *gapA* amplification being employed as a housekeeping control. Differential expression was recorded as an absolute fold change of ≥ 1.5; ≤ -1.5 and $p \leq 0.05$ as determined by two-tailed Student's *t*-test.

Construction of isogenic EHEC mutant strains

Mutants lacking *cycA*, *sstT* and *cscR* were prepared as previously described (10). Briefly, EHEC WT was transformed with pKD46, cultured at 37°C in super optimal broth supplemented with 100 µg ml^{-1} ampicillin and 100 mM L-arabinose to an OD600 nm of 0.4, before washing three times with ice-cold distilled water and resuspending in ice-cold distilled water. A linear deletion fragment was prepared by amplifying the

chloramphenicol resistance cassette from pKD3 with oligonucleotides bearing 50 bp 5'-end flanking regions bearing homology to the 50 bp regions immediately upstream and downstream of the chosen gene (SI Appendix, Table S6). One microgram of phenol-chloroform extracted PCR product was electroporated at 2,500 V into each aliquot of washed lambda red competent cells using an Eppendorf Eporator. Insertional mutants cultivated under chloramphenicol selection were verified by PCR using check primers (SI Appendix, Table S6). Resistance cassettes were removed by expression of FLP-recombinase under transient temperature shift to 42°C after transformation of insertional mutants with pCP20. Excision of the resistance cassette was confirmed by PCR.

Fluorimetric D-ser assay

Concentrations of D-ser were determined using DL-serine assay kit (Abcam) according to manufacturer's instructions. At the indicated time-points, cell suspensions were centrifuged at 12,000 × g, one minute before passing supernatant samples through a 0.22 µm filter and storing at -20°C for later analysis. Standards of varying concentrations of D-ser were prepared in MEM-HEPES medium. One microliter of each standard or sample was mixed with 29 µl assay buffer on a black microtiter plate. Twenty microliters of reaction mixture containing one microliter developer, 0.5 µl probe solution, 0.5 µl D-ser enzyme mix and 18 µl assay buffer was added. Reactions were briefly mixed before incubation at 37°C for one hour in a humidity chamber protected from light. Fluorescence was recorded in a Varioskan Lux plate reader (Thermofisher Scientific) at 535 nm excitation, 587 nm emission. Sample concentrations were recorded derived from the standard curve. Triplicate experiments were performed.

Specific growth rate (SGR) determination

Exponential growth was observed between two and five hours for all of the media tested. SGR (μ) was calculated as $\mu = (\Delta \ln OD_{600\ nm})/\Delta t$ where $\Delta \ln OD_{600\ nm}$ is the change in natural logarithm of $OD_{600\ nm}$ and Δt is the change in time (hours) between two and five hours. $OD_{600\ nm}$ was recorded in an Eppendorf D30 Biophotometer. At least three replicate growth curves were constructed for determination of SGR of any given strain.

Growth on minimal medium with sucrose as a sole carbon source

Solid MOPS minimal medium was prepared by supplementing cooled 1.5 % (w/v) agar to a final concentration of 1× MOPS Buffer (Teknova), 1.32 mM K_2HPO_4, 100 ng ml^{-1} thiamine and 0.4% (w/v) sucrose. Indicated strains were streaked on dry agar plates and incubated at 37°C, 48 h before imaging using a Biorad ChemiDoc MP Imaging System.

Analysis of *dsdCXA* transcription by RT-PCR during CscR expression in UPEC

UPEC WT and UPEC WT (p*cscR*) were cultured in M9 and M9, 20 μg ml^{-1} chloramphenicol respectively for 3 h at 37°C. A sample was taken and stabilized in RNAprotect Bacteria Reagent (Qiagen). D-ser was added to a concentration of one millimolar and further samples were taken and stabilized at the indicated timepoints. RNA was extracted and cDNA synthesized as for qRT-PCR above. One microliter of cDNA was used in 12.5 μl GoTaq (Promega) reactions with 95°C, 2 min initial denaturation, followed by 95°C; 20 sec denaturation, 55°C; 20 sec annealing 72°C; 20

sec extension for 28 cycles being employed. Samples were electrophoresed at 100V on a 2% (w/v) agarose gel.

Bioinformatic Analysis

Promoter sequences were extracted from RegulonDB for all genes possessed by *E. coli* K-12 (11). For *csc* genes, promoter positions were predicted using SoftBerry BProm (12).

Statistical analysis

Statistical significance was assessed by two-tailed Student's t-test or ordinary one-way ANOVA with post multiple comparisons test as indicated. Tests were carried out using GraphPad Prism 8.

Supplementary Figures

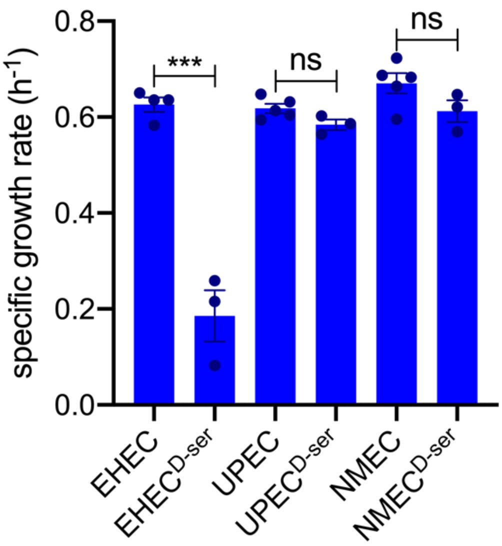

Fig. S1. Specific growth rates of *E. coli* pathotypes with and without D-ser. Specific growth rates were calculated as outlined in methods from at least three independent growth curves. Bars represent SEM. Statistical significance was tested using unpaired two-tailed Student's *t*-test.

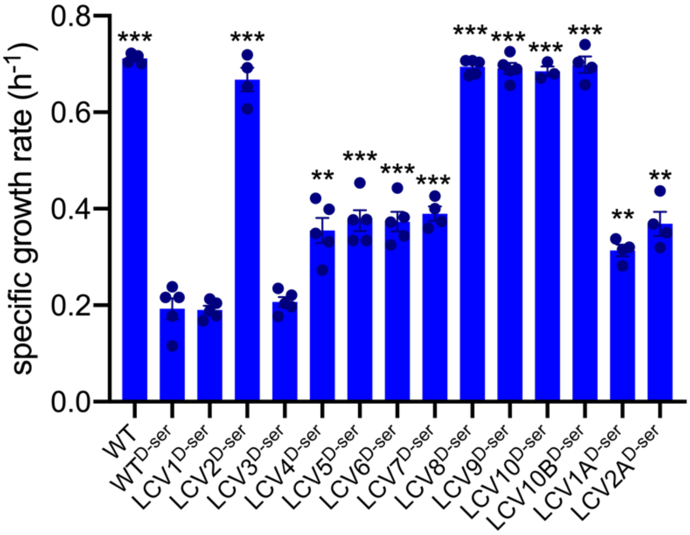

Fig. S2. Specific growth rates of isolates obtained from D-ser adaptation. Specific growth rates were calculated as outlined in methods from at least three independent growth curves. Bars represent SEM. Statistical significance was tested by comparing each sample to the $WT^{D\text{-}ser}$ using unpaired two-tailed Student's *t*-test.

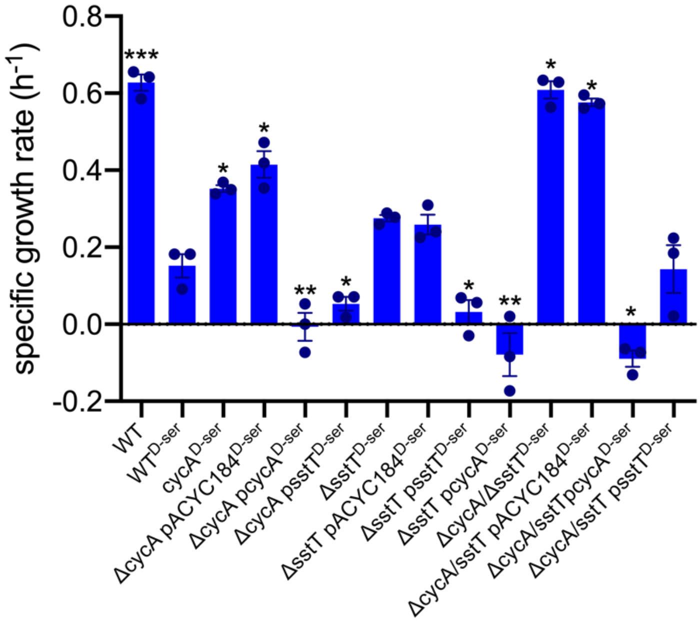

Fig. S3. Specific growth rates of isogenic D-ser transporter deletion mutants and complemented strains. Specific growth rates were calculated as outlined in methods from at least three independent growth curves. Bars represent SEM. Statistical significance was tested by comparing each sample to the WT$^{\text{D-ser}}$ using paired two-tailed Student's *t*-test.

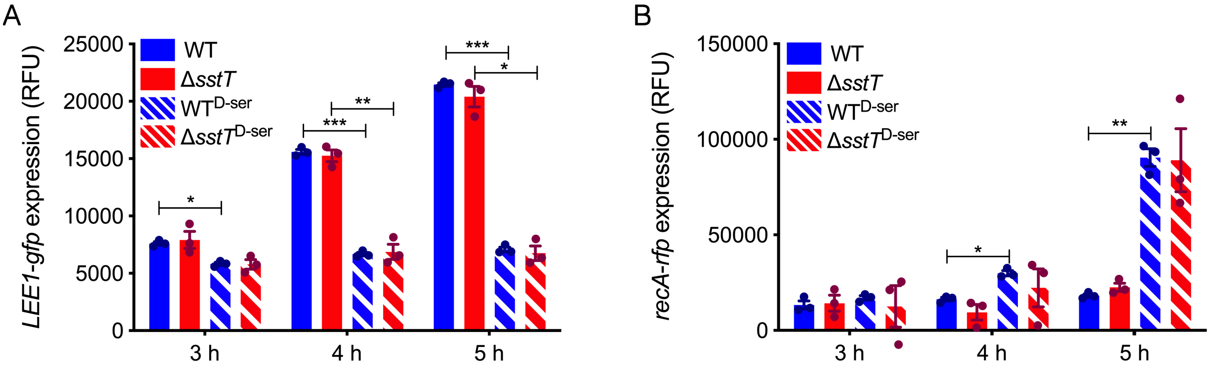

Fig. S4. Deletion of *sstT* does not affect repression of type 3 secretion or activation of *recA* in response to D-ser. (A and B) Dual *LEE1-gfp* (A)/*recA-rfp* (B) promoter fusion reporter analysis of WT and *sstT* mutants cultured in MEM-HEPES at the indicated timepoints. Three experiments were performed, with error bars depicting SEM. *, **, *** indicate $p < 0.05$, 0.01 and 0.001 respectively by paired two-tailed Student's *t*-test.

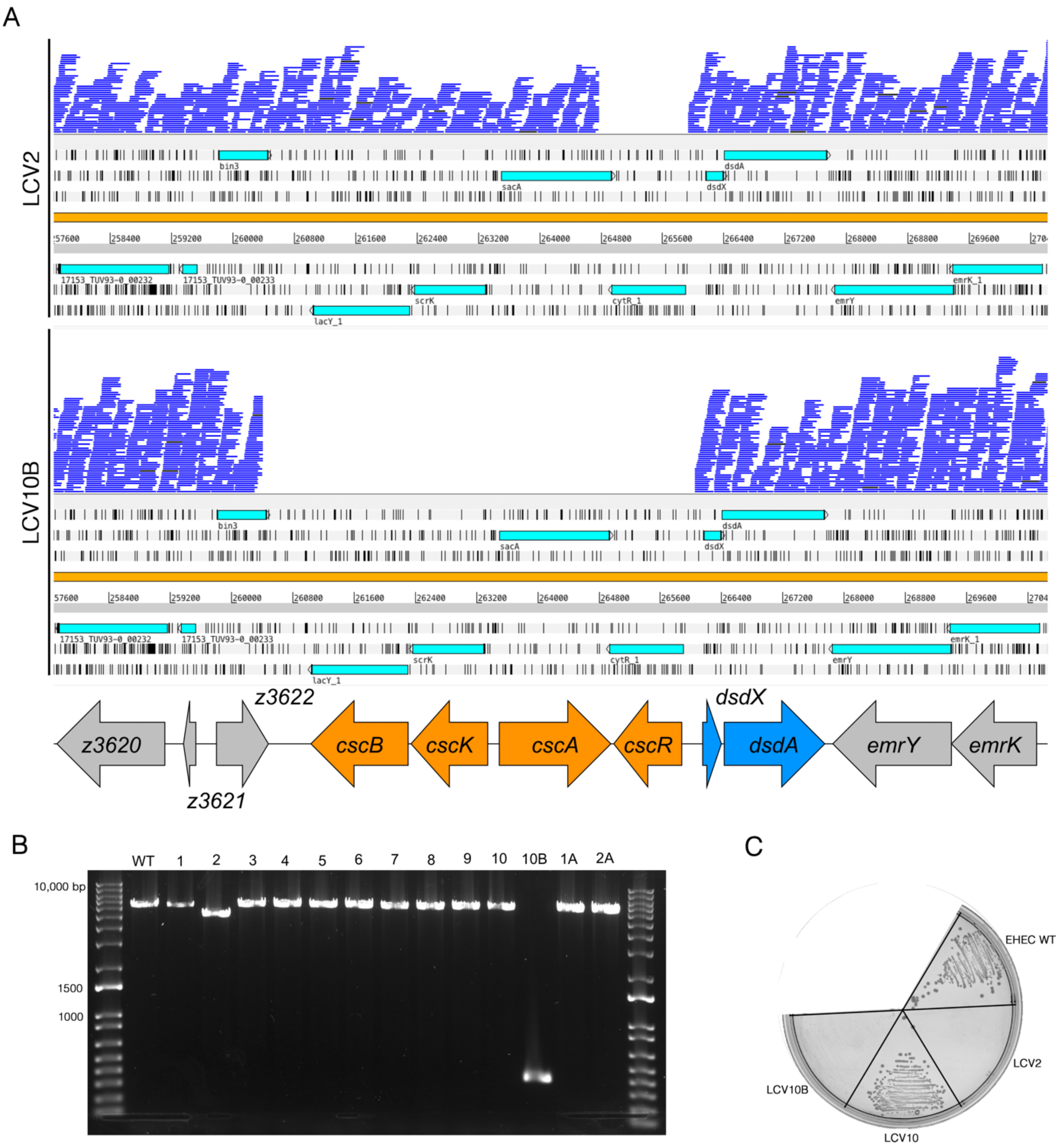

Fig. S5. Identification of *cscR* and *cscBKAR* deletions in LCV2 and LCV10B. (A) Mapping of Illumina NextSeq read data to the EHEC genome in Artemis Genome Browser revealing large regions with no read mapping indicative of the loss of these genomic elements in LCV2 and LCV10B. (B) PCR confirming the truncation of the region upstream of *dsdA* using primers highlighted in Fig. 6A. (C) Culture of the indicated strains on MOPS minimal medium with 0.4% (w/v) sucrose as a sole carbon source.

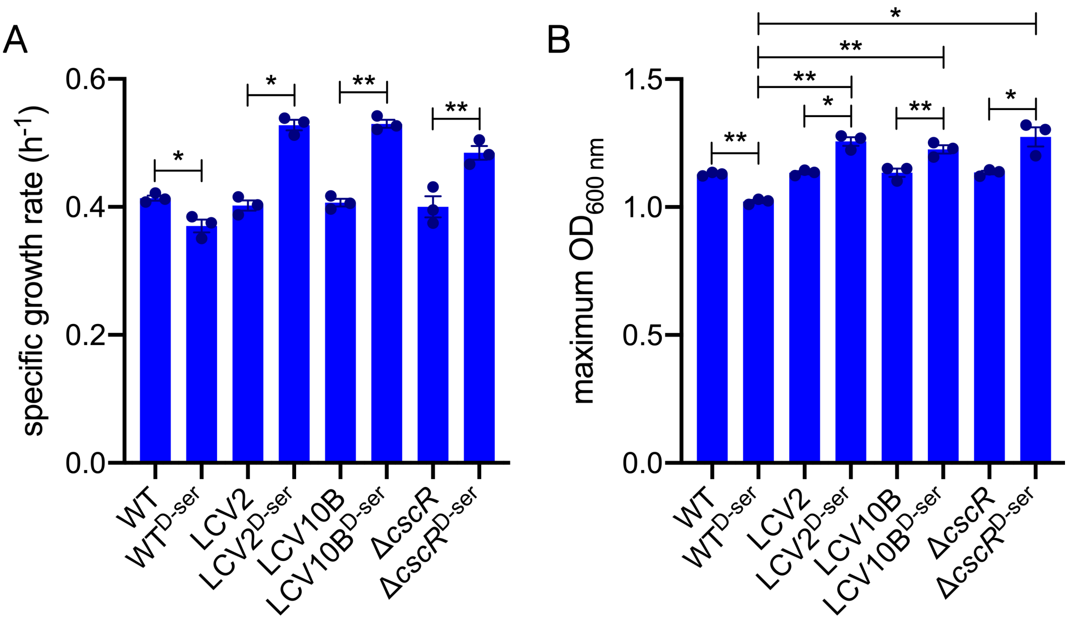

Fig. S6. Specific growth rates and maximum OD600 nm of LCV2, 10B and Δ*cscR* in MEM-HEPES with and without D-ser. (A) Specific growth rates were calculated as outlined in methods from three independent growth curves. (B) Mean maximal OD600 nm values from three independent experiments are presented. Error bars represent SEM and statistical significance was tested using paired two-tailed Student's *t*-test (A and B).

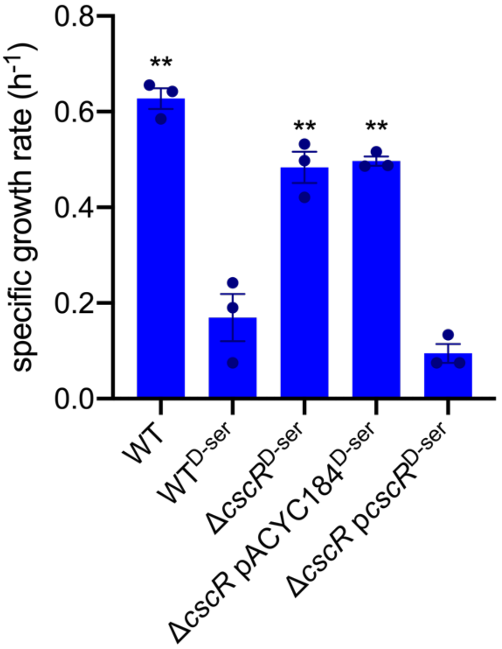

Fig. S7. Specific growth rates of Δ*cscR* and corresponding complementation strains in M9 with and without D-ser. Specific growth rates were calculated as outlined in methods from at least three independent growth curves. Bars represent SEM. Statistical significance was tested by comparing each sample to the WT$^{D\text{-}ser}$ using unpaired two-tailed Student's *t*-test.

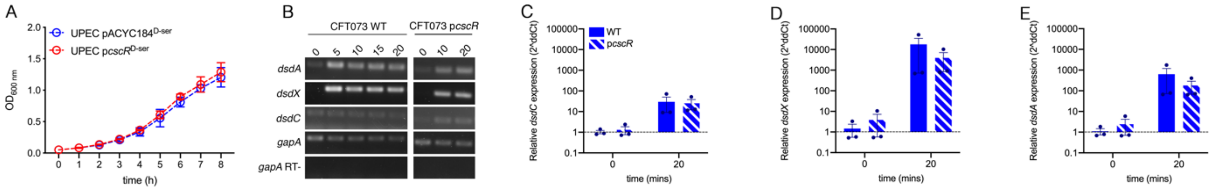

Fig. S8. CscR does not repress *dsd* in UPEC. (A) Growth curves of UPEC with empty pACYC184 compared with p*cscR* cultured in M9+Glc+D-ser. Error bars indicate SEM with three experiments being performed. (B) RT-PCR of *dsdCXA* and the housekeeping control *gapA* at the indicated timepoint (minutes) post addition of D-ser. Experiment was performed in M9+Glc with 3 h pre-growth prior to addition of D-ser. (C-E) qRT-PCR of *dsdC* (C), *dsdX* (D) and *dsdA* (E) transcription at steady state (0 mins) and 20 min after spiking with D-ser (i.e. 3 h 20 m total, as in panel B) in UPEC WT and UPEC p*cscR*. Transcription of each gene was expressed relative to WT at 0 min. Experiment was performed in triplicate with error bars indicating SEM. No significant differences were observed with trans expression of *cscR*.

Table S1. Genes differentially expressed comparing EHEC WT with EHEC WT D-ser by RNA-seq and comparison of directionality with LCV10 D-ser

Feature ID	Fold change WT vs WT D-ser	FDR corrected p-value	Fold Change WT D-ser vs 10 D-ser	Direction of expression in WT D-ser vs LCV10 D-ser
yhaF	183.86	1.53E-57	-92.93	Opposite
yhaE	121.04	1.57E-73	-125.07	Opposite
yhaU	75.48	5.53E-48	-302.08	Opposite
Z0310	61.39	2.72E-20	-35.4	Opposite
Z2977	43.64	2.47E-22	-35.78	Opposite
yhaD	41.23	1.70E-55	-29.65	Opposite
Z4105	35.2	1.07E-32	-74.86	Opposite
Z2979	21.36	5.62E-29	-20.14	Opposite
Z0312	20.34	1.06E-14	-24.45	Opposite
Z2978	17.36	5.32E-27	-29.08	Opposite
Z4104	17.02	2.72E-10	-7.32	Opposite
Z2974	16.85	1.54E-29	-31.98	Opposite
ygcX	15.2	3.59E-21	-11.49	Opposite
yhaC	14.42	2.17E-03	#N/A	No change
Z0311	14.4	2.84E-17	-14.68	Opposite
Z2975	14.04	3.73E-13	-20.16	Opposite
Z0313	12.96	3.14E-13	-14.82	Opposite
yhaG	12.27	8.39E-20	-13.43	Opposite
intH	12.17	1.19E-27	-20.32	Opposite
ygcY	10.5	8.48E-13	-6.53	Opposite
Z0314	10.06	9.75E-14	-9.74	Opposite
yaeG	9.1	8.73E-18	-7.53	Opposite
Z0317	8.62	4.45E-14	-20.13	Opposite
recN	8.5	1.65E-20	-14.08	Opposite
dinD	7.24	1.17E-12	-6.28	Opposite
Z0316	7.17	2.57E-13	-9.84	Opposite
p0044-p0045	6.96	0.02	#N/A	No change
umuD	6.64	2.63E-11	-11.49	Opposite
Z0315	6.49	1.06E-07	-7.77	Opposite
sulA	6.25	3.44E-16	-8.12	Opposite
Z2980	6.05	1.73E-04	-4.37	Opposite
tnaA	5.72	3.75E-08	-4	Opposite
afuA	5.66	1.12E-05	#N/A	No change
recA	5.46	1.45E-14	-7.46	Opposite
Z2976	5.3	5.47E-05	-9.2	Opposite
oraA	5.16	6.68E-06	-9.96	Opposite
umuC	4.92	1.60E-10	-7.43	Opposite
Z2973	4.89	3.11E-10	-12.8	Opposite

dinI	4.89	1.65E-09	-4.54	Opposite
Z2971	4.41	6.16E-10	-9.69	Opposite
yjcH	4.29	0.01	-4.91	Opposite
dinF	3.98	6.49E-08	-6.84	Opposite
dinP	3.91	1.66E-07	-3.83	Opposite
Z2972	3.88	8.03E-07	-7.98	Opposite
aldH	3.84	3.12E-03	-2.8	Opposite
lexA	3.8	7.36E-09	-4.03	Opposite
Z1450	3.75	0.05	#N/A	No change
Z0461	3.74	2.11E-04	#N/A	No change
ilvB	3.5	1.18E-06	-3.27	Opposite
Z1210	3.46	0.05	#N/A	No change
Z1641	3.43	0.01	#N/A	No change
cspA	3.18	1.24E-04	-2.75	Opposite
yebG	3.14	2.92E-06	-3.49	Opposite
goaG	3.03	8.74E-03	#N/A	No change
ordL	2.94	0.03	-3.84	Opposite
ycjL	2.92	6.63E-03	-3.61	Opposite
yigN	2.92	2.14E-04	-2.81	Opposite
Z3109	2.92	5.65E-03	-2.23	Opposite
uvrA	2.74	2.84E-05	-2.75	Opposite
acs	2.68	1.73E-04	-2.15	Opposite
yebF	2.66	1.96E-04	-3.19	Opposite
Z1651	2.66	2.69E-04	3.01	Further up
Z3271	2.65	8.72E-04	-2.98	Opposite
yjfR	2.62	0.05	-2.28	Opposite
ilvN	2.57	0.04	-3.3	Opposite
Z3970	2.57	0.03	-3.09	Opposite
Z2491	2.53	3.68E-03	-4.99	Opposite
mglA	2.53	8.23E-04	-1.8	Opposite
gltB	2.51	1.29E-04	-2	Opposite
soxS	2.5	3.04E-03	-2.56	Opposite
uvrD	2.5	2.92E-04	-2.36	Opposite
yjbO	2.5	4.57E-03	#N/A	No change
hisG	2.48	5.58E-04	-2.98	Opposite
sdhD	2.47	1.05E-03	-2.2	Opposite
p0045	2.46	8.26E-04	-2.63	Opposite
Z1200	2.44	0.03	#N/A	No change
fimB	2.43	3.02E-03	-1.83	Opposite
sbmC	2.41	0.02	-1.89	Opposite
pstB	2.4	4.23E-03	-2.47	Opposite
htrL	2.39	1.10E-03	-2.27	Opposite
mglB	2.39	8.97E-04	#N/A	No change

purH	2.38	4.20E-04	-3.54	Opposite
pckA	2.37	5.19E-04	-2.25	Opposite
coxT	2.36	5.36E-03	-5.14	Opposite
asnA	2.35	3.56E-04	-2.44	Opposite
uvrB	2.35	6.63E-04	-2.29	Opposite
Z2771	2.32	6.98E-03	-2.23	Opposite
uhpA	2.29	0.04	#N/A	No change
pstS	2.28	8.37E-03	#N/A	No change
glyA	2.26	1.08E-03	-2.9	Opposite
Z0321	2.25	7.01E-03	#N/A	No change
Z1193	2.25	9.50E-03	#N/A	No change
tatD	2.24	0.02	-2.15	Opposite
mglC	2.24	9.50E-03	-1.72	Opposite
kbl	2.23	1.79E-03	-1.85	Opposite
fadB	2.22	7.48E-03	-2.31	Opposite
deaD	2.22	1.75E-03	-2.26	Opposite
serA	2.22	1.61E-03	-1.62	Opposite
hisD	2.2	3.84E-03	-2.93	Opposite
p0046	2.2	1.83E-04	-2.57	Opposite
Z1057	2.2	0.02	-2.2	Opposite
purD	2.19	2.09E-03	-3.24	Opposite
Z0722	2.19	0.04	-1.87	Opposite
yafB	2.18	0.01	-1.71	Opposite
fadL	2.15	8.99E-03	-2.11	Opposite
Z1579	2.13	7.72E-03	-1.98	Opposite
tdh	2.11	4.77E-03	-1.85	Opposite
Z3973	2.11	0.04	#N/A	No change
Z2751	2.1	0.05	-2.21	Opposite
Z0879	2.1	0.03	-1.9	Opposite
Z1341	2.08	5.65E-03	-2.45	Opposite
gidA	2.08	6.46E-03	-1.94	Opposite
Z3272	2.06	0.03	-2.41	Opposite
gltD	2.05	6.84E-03	-1.89	Opposite
sucD	2.05	6.57E-03	-1.8	Opposite
ackA	2.03	7.13E-03	-2.68	Opposite
Z1020	2.03	0.02	-2.03	Opposite
Z0067	2.01	0.02	-2.34	Opposite
yjgD	2.01	0.01	-1.95	Opposite
gcvT	2	0.01	-4.52	Opposite
sdhB	2	0.01	-2.23	Opposite
sucB	2	9.30E-03	-2.2	Opposite
sucC	2	9.14E-03	-1.92	Opposite
sdhA	1.98	0.01	-2.6	Opposite

sucA	1.97	0.01	-2.31	Opposite
Z1211	1.97	0.03	3.25	Further up
gidB	1.95	0.05	-2.07	Opposite
pta	1.94	0.01	-2.25	Opposite
murI	1.91	0.04	-1.88	Opposite
Z1186	1.91	0.03	-1.57	Opposite
yojH	1.88	0.03	-5.85	Opposite
sdhC	1.86	0.05	-2.07	Opposite
ruvA	1.84	0.05	-1.71	Opposite
ssrS	1.84	0.03	#N/A	No change
sodA	1.83	0.04	-2.39	Opposite
Z4386	1.83	0.04	-1.59	Opposite
tir	-1.77	0.05	#N/A	No change
Z1617	-1.8	0.04	4.19	Opposite
Z1178	-1.8	0.04	4.21	Opposite
ompF	-1.8	0.04	#N/A	No change
hisJ	-1.8	0.05	#N/A	No change
nupC	-1.8	0.05	#N/A	No change
cydA	-1.83	0.04	3.81	Opposite
gadA	-1.89	0.03	#N/A	No change
bax	-1.9	0.02	2.43	Opposite
sepL	-1.9	0.02	#N/A	No change
hisM	-1.9	0.04	#N/A	No change
cls	-1.91	0.03	2.17	Opposite
Z0957	-1.92	0.03	#N/A	No change
Z6024	-1.95	0.03	-2.43	Further down
osmE	-1.95	0.04	2.1	Opposite
Z2054	-1.95	0.02	2.37	Opposite
tesB	-1.96	0.03	#N/A	No change
yeaD	-1.97	0.02	2.25	Opposite
Z2287	-1.97	0.05	2.65	Opposite
escN	-1.97	0.02	#N/A	No change
Z5111	-2.01	8.47E-03	#N/A	No change
gadB	-2.02	8.97E-03	1.96	Opposite
dadA	-2.02	9.57E-03	#N/A	No change
eae	-2.03	6.98E-03	-1.59	Further down
metK	-2.03	7.72E-03	1.59	Opposite
hisP	-2.03	0.01	#N/A	No change
rseA	-2.05	7.01E-03	1.53	Opposite
espB	-2.05	5.94E-03	#N/A	No change
espD	-2.05	5.94E-03	#N/A	No change
Z1054	-2.06	0.01	#N/A	No change
yebA	-2.07	0.01	2.93	Opposite

Z0955	-2.08	5.34E-03	-1.64	Further down
wrbA	-2.09	0.04	2.94	Opposite
yccJ	-2.1	0.03	2.79	Opposite
xasA	-2.12	4.37E-03	2.02	Opposite
espA	-2.12	3.27E-03	#N/A	No change
Z2185	-2.13	0.05	#N/A	No change
Z1931	-2.14	2.95E-03	3.19	Opposite
pspE	-2.14	0.02	6.01	Opposite
sepQ	-2.14	5.18E-03	#N/A	No change
Z5142	-2.15	4.04E-03	#N/A	No change
fsr	-2.15	0.03	#N/A	No change
rpoE	-2.17	3.29E-03	#N/A	No change
Z5104	-2.2	2.03E-03	#N/A	No change
hisQ	-2.22	2.52E-03	#N/A	No change
escD	-2.23	2.63E-03	#N/A	No change
escU	-2.23	0.01	#N/A	No change
yaiB	-2.25	8.37E-03	#N/A	No change
yieE	-2.26	0.01	#N/A	No change
yraQ	-2.28	0.02	#N/A	No change
xylE	-2.29	0.02	#N/A	No change
yggB	-2.3	9.76E-04	2.08	Opposite
fruK	-2.31	6.67E-03	3.29	Opposite
rpsV	-2.33	6.84E-03	2.81	Opposite
metA	-2.33	7.01E-03	#N/A	No change
fdhF	-2.34	2.74E-03	1.85	Opposite
Z1930	-2.34	6.66E-04	3.83	Opposite
ybaY	-2.35	1.71E-03	2.72	Opposite
ecnB	-2.36	0.01	2.46	Opposite
Z3260	-2.36	9.85E-04	2.74	Opposite
talA	-2.36	2.78E-03	3.72	Opposite
glnQ	-2.37	1.34E-03	2.33	Opposite
cycA	-2.38	4.09E-04	-2.9	Further down
Z1925	-2.38	3.09E-03	3.07	Opposite
pspA	-2.38	5.11E-04	11.2	Opposite
yddA	-2.39	1.41E-03	2.88	Opposite
Z6054	-2.4	0.03	2.13	Opposite
Z1921	-2.4	8.51E-03	2.41	Opposite
dadX	-2.4	7.15E-04	#N/A	No change
csgD	-2.41	6.33E-04	2.74	Opposite
Z1055	-2.41	6.98E-03	#N/A	No change
leuO	-2.41	0.02	#N/A	No change
yjjU	-2.43	3.67E-03	1.79	Opposite
rmf	-2.43	2.69E-04	3.65	Opposite

Z1051	-2.45	5.78E-03	#N/A	No change
csgG	-2.46	1.05E-03	2.6	Opposite
yedU	-2.48	2.88E-04	3.31	Opposite
espF	-2.49	2.13E-04	#N/A	No change
ydfG	-2.51	3.42E-04	#N/A	No change
Z5102	-2.52	2.70E-04	#N/A	No change
mtr	-2.55	4.47E-03	3.98	Opposite
Z5143	-2.55	4.06E-03	#N/A	No change
Z1972	-2.56	6.33E-04	3.14	Opposite
Z4214	-2.59	4.77E-03	#N/A	No change
Z1052	-2.61	6.66E-04	1.85	Opposite
Z3043	-2.61	0.03	5.29	Opposite
Z5117	-2.62	2.09E-03	#N/A	No change
glnH	-2.63	8.68E-05	2.53	Opposite
Z1056	-2.64	6.98E-03	2.54	Opposite
escF	-2.64	1.64E-04	#N/A	No change
fruA	-2.65	2.72E-04	3.34	Opposite
escT	-2.65	1.14E-03	#N/A	No change
p0024	-2.67	1.20E-06	#N/A	No change
Z2883	-2.69	0.02	3.48	Opposite
pspD	-2.7	7.67E-03	10.2	Opposite
Z1062	-2.73	7.73E-03	3.91	Opposite
p0025	-2.74	4.33E-07	#N/A	No change
pspB	-2.75	3.75E-03	10.38	Opposite
yohC	-2.81	0.04	2.54	Opposite
Z3023	-2.82	0.02	#N/A	No change
Z3045	-2.83	1.42E-03	3.44	Opposite
elaB	-2.84	9.63E-05	4.3	Opposite
p0023	-2.88	1.94E-07	#N/A	No change
potH	-2.89	8.63E-05	#N/A	No change
dps	-2.9	5.01E-06	4.65	Opposite
yjaI	-2.91	0.04	#N/A	No change
yddB	-2.95	7.82E-06	3.4	Opposite
yjbA	-2.97	1.19E-04	1.98	Opposite
osmY	-2.98	2.02E-05	2.84	Opposite
csgE	-2.99	5.47E-05	3.15	Opposite
glnP	-3	2.06E-05	2.71	Opposite
Z1823	-3	4.72E-04	#N/A	No change
fruB	-3.01	2.19E-04	4.55	Opposite
pqqL	-3.07	2.63E-06	2.77	Opposite
ycgB	-3.08	6.33E-04	3.2	Opposite
yjbH	-3.1	1.71E-03	#N/A	No change
otsA	-3.12	1.41E-05	3.45	Opposite

csgF	-3.13	1.06E-04	3.6	Opposite
chaC	-3.14	3.44E-06	2.78	Opposite
yieG	-3.14	7.40E-07	#N/A	No change
Z0923	-3.15	1.46E-03	4.46	Opposite
tktB	-3.17	2.83E-06	4.13	Opposite
Z3074	-3.19	7.38E-03	2.94	Opposite
potG	-3.2	2.90E-06	#N/A	No change
potI	-3.2	1.92E-05	#N/A	No change
potF	-3.22	6.17E-07	#N/A	No change
ugd	-3.26	3.84E-06	#N/A	No change
Z2379	-3.28	8.32E-03	3.35	Opposite
poxB	-3.31	2.81E-06	4.55	Opposite
ygeX	-3.38	0.04	#N/A	No change
yeaH	-3.4	2.83E-06	2.85	Opposite
wcaF	-3.4	0.03	#N/A	No change
ygaE	-3.41	4.09E-06	2.27	Opposite
Z1824	-3.41	2.37E-07	#N/A	No change
Z5978	-3.43	1.95E-04	2.98	Opposite
pspC	-3.44	6.81E-06	10.26	Opposite
yahO	-3.56	2.38E-03	4.88	Opposite
ynfM	-3.61	3.28E-08	#N/A	No change
wzxC	-3.73	7.79E-03	#N/A	No change
yeaQ	-3.75	1.18E-04	3.77	Opposite
otsB	-3.78	5.84E-06	4.84	Opposite
Z2239	-3.78	7.98E-03	#N/A	No change
Z2227	-3.89	3.06E-06	#N/A	No change
Z1678	-3.89	0.03	#N/A	No change
ygaM	-3.9	1.11E-04	6.08	Opposite
Z2277	-3.95	0.03	#N/A	No change
Z4486	-3.97	0.02	#N/A	No change
yciF	-4.01	1.42E-04	2.77	Opposite
Z4018	-4.08	2.91E-04	3.82	Opposite
Z2269	-4.12	7.59E-05	4.86	Opposite
Z2275	-4.16	1.20E-07	2.39	Opposite
Z4223	-4.2	1.56E-06	#N/A	No change
adhP	-4.3	4.72E-07	5.64	Opposite
metF	-4.31	4.96E-08	#N/A	No change
katE	-4.45	2.61E-09	4.6	Opposite
yebL	-4.55	6.37E-11	6.23	Opposite
ygfP	-4.58	1.88E-09	2	Opposite
wcaC	-4.66	3.76E-04	#N/A	No change
flgE	-4.72	2.92E-04	#N/A	No change
Z3224	-4.73	4.92E-06	#N/A	No change

wcaA	-4.76	4.08E-04	#N/A	No change
codA	-4.9	2.76E-12	1.64	Opposite
yeaG	-4.9	4.35E-12	3.46	Opposite
Z3249	-5.01	1.01E-07	7.73	Opposite
metE	-5.17	9.97E-14	1.59	Opposite
yjbF	-5.21	1.32E-03	#N/A	No change
wcaL	-5.3	9.41E-06	#N/A	No change
argT	-5.58	0	2.9	Opposite
ydaK	-5.58	1.02E-09	6.09	Opposite
Z2279	-5.63	4.39E-06	#N/A	No change
agaV	-5.63	0.02	#N/A	No change
wcaD	-5.67	1.25E-07	#N/A	No change
rcsA	-5.87	3.21E-13	1.98	Opposite
Z2351	-6.28	7.24E-03	5.58	Opposite
wcaE	-6.3	5.03E-08	#N/A	No change
Z2276	-6.42	1.71E-03	#N/A	No change
Z2777	-6.73	1.34E-16	4.45	Opposite
Z2226	-6.81	1.34E-09	#N/A	No change
Z2428	-6.83	2.37E-08	2.66	Opposite
csgC	-6.94	2.58E-03	#N/A	No change
cpsG	-7.09	1.96E-09	#N/A	No change
wza	-7.13	3.11E-07	#N/A	No change
Z1922	-7.39	1.25E-03	#N/A	No change
ydjS	-7.47	2.06E-17	4.19	Opposite
Z3065	-7.88	4.58E-15	10.3	Opposite
cstC	-7.93	1.19E-19	3.78	Opposite
wzb	-7.94	2.66E-03	#N/A	No change
wcaI	-7.95	2.35E-12	#N/A	No change
yhdZ	-7.99	9.60E-17	3.06	Opposite
wcaJ	-8.24	8.49E-08	#N/A	No change
Z2778	-8.25	4.80E-20	4.1	Opposite
codB	-8.45	4.46E-20	2	Opposite
wcaH	-8.56	9.30E-10	#N/A	No change
yjbE	-8.63	2.21E-14	#N/A	No change
flgD	-8.64	8.37E-03	#N/A	No change
yjbG	-8.86	3.95E-05	#N/A	No change
amtB	-9.31	0	5.68	Opposite
wcaK	-9.46	4.98E-08	#N/A	No change
glnK	-9.63	3.96E-23	5.81	Opposite
Z2779	-9.65	3.54E-22	4.76	Opposite
gabP	-9.67	1.72E-15	5.28	Opposite
yhdX	-9.9	1.45E-18	3.27	Opposite
Z2225	-10.23	6.60E-11	#N/A	No change

cbl	-10.44	9.19E-25	4.32	Opposite
ygjG	-10.79	5.23E-25	3.78	Opposite
gmd	-11.25	4.46E-20	#N/A	No change
gabD	-11.56	3.21E-22	6.75	Opposite
fliA	-11.82	0.01	#N/A	No change
cpsB	-12.18	4.42E-15	#N/A	No change
yedL	-12.28	4.90E-26	8.38	Opposite
yhdY	-12.48	3.60E-19	2.44	Opposite
ygjH	-12.48	0.01	#N/A	No change
nac	-12.62	1.04E-28	5.17	Opposite
wcaG	-12.64	1.45E-18	#N/A	No change
gabT	-12.83	4.65E-23	6.44	Opposite
yhdW	-15.59	8.91E-30	4.54	Opposite
Z1509	-17.77	6.93E-21	5.78	Opposite
Z2224	-18.24	5.54E-21	3.16	Opposite
Z1506	-18.48	1.51E-22	4.55	Opposite
csgA	-19.54	8.30E-27	6.57	Opposite
Z1508	-21.12	1.94E-28	6.11	Opposite
Z2223	-23.54	2.11E-37	5.42	Opposite
ycdG	-25.41	3.15E-35	6.97	Opposite
ydaJ	-25.75	1.04E-30	6.83	Opposite
csgB	-25.92	2.94E-28	9.09	Opposite
Z2222	-27.06	3.15E-35	5.22	Opposite
Z2427	-27.36	1.17E-29	8.29	Opposite
Z1924	-28.21	3.54E-13	24.73	Opposite
flgB	-31.29	0.03	#N/A	No change
Z1510	-33.6	5.04E-36	9.97	Opposite
Z1507	-34.17	3.87E-31	9.79	Opposite
Z1511	-34.52	1.18E-43	8.73	Opposite
Z1923	-39.84	1.87E-09	20.1	Opposite

Table S2. Genes differentially expressed comparing EHEC WT D-ser with LCV10 D-ser by RNA-seq and comparison of directionality with WT D-ser

Feature ID	Fold change WT D-ser vs 10 D-ser	FDR corrected p-value	Fold Change WT vs WT D-ser	Direction of expression in WT D-ser vs LCV10 D-ser
Z0952	25.28	0.04	#N/A	New DEG
Z1924	24.73	1.57E-13	-28.21	Opposite
Z1923	20.1	1.16E-05	-39.84	Opposite
pspA	11.2	2.40E-54	-2.38	Opposite
pspB	10.38	7.26E-28	-2.75	Opposite
Z3065	10.3	2.45E-19	-7.88	Opposite
pspC	10.26	2.10E-33	-3.44	Opposite

pspD	10.2	1.05E-24	-2.7	Opposite
Z1510	9.97	4.99E-19	-33.6	Opposite
Z1507	9.79	8.40E-15	-34.17	Opposite
csgB	9.09	1.38E-27	-25.92	Opposite
Z1511	8.73	7.70E-23	-34.52	Opposite
yedL	8.38	1.41E-32	-12.28	Opposite
Z2427	8.29	1.00E-14	-27.36	Opposite
Z3956	7.9	4.87E-19	#N/A	New DEG
Z3249	7.73	3.98E-18	-5.01	Opposite
Z5095	7.57	0.01	#N/A	New DEG
ycdG	6.97	2.49E-19	-25.41	Opposite
ydaJ	6.83	1.03E-13	-25.75	Opposite
gabD	6.75	6.54E-21	-11.56	Opposite
Z2594	6.62	9.48E-09	#N/A	New DEG
csgA	6.57	1.33E-26	-19.54	Opposite
gabT	6.44	2.18E-18	-12.83	Opposite
yebL	6.23	5.30E-17	-4.55	Opposite
Z1508	6.11	6.54E-13	-21.12	Opposite
ydaK	6.09	2.10E-15	-5.58	Opposite
ygaM	6.08	1.36E-11	-3.9	Opposite
pspE	6.01	6.89E-21	-2.14	Opposite
glnK	5.81	8.13E-26	-9.63	Opposite
Z1509	5.78	3.87E-08	-17.77	Opposite
amtB	5.68	7.26E-28	-9.31	Opposite
adhP	5.64	1.30E-14	-4.3	Opposite
Z2351	5.58	8.02E-03	-6.28	Opposite
ygaF	5.5	2.21E-12	#N/A	New DEG
uxuA	5.43	1.52E-18	#N/A	New DEG
Z2223	5.42	4.87E-19	-23.54	Opposite
terF	5.35	0.04	#N/A	New DEG
Z3043	5.29	1.51E-09	-2.61	Opposite
gabP	5.28	1.52E-10	-9.67	Opposite
Z2222	5.22	3.17E-11	-27.06	Opposite
nac	5.17	5.26E-24	-12.62	Opposite
uxaC	4.95	2.21E-15	#N/A	New DEG
yahO	4.88	8.24E-07	-3.56	Opposite
Z2269	4.86	4.21E-08	-4.12	Opposite
otsB	4.84	3.81E-12	-3.78	Opposite
Z2779	4.76	1.39E-19	-9.65	Opposite
Z2201	4.65	0.01	#N/A	New DEG
dps	4.65	3.15E-22	-2.9	Opposite
katE	4.6	1.90E-15	-4.45	Opposite
fruB	4.55	1.21E-12	-3.01	Opposite

poxB	4.55	2.40E-16	-3.31	Opposite
Z1506	4.55	3.81E-06	-18.48	Opposite
yhdW	4.54	1.32E-15	-15.59	Opposite
phnB	4.49	5.29E-04	#N/A	New DEG
Z0923	4.46	1.69E-08	-3.15	Opposite
Z2777	4.45	6.31E-18	-6.73	Opposite
Z2098	4.4	4.24E-04	#N/A	New DEG
cbl	4.32	1.81E-18	-10.44	Opposite
elaB	4.3	3.40E-15	-2.84	Opposite
Z1178	4.21	5.55E-20	-1.8	Opposite
Z1617	4.19	7.22E-20	-1.8	Opposite
ydjS	4.19	8.17E-15	-7.47	Opposite
tktB	4.13	4.01E-16	-3.17	Opposite
Z2778	4.1	4.91E-16	-8.25	Opposite
Z2396	4.05	0.01	#N/A	New DEG
Z2813	4.04	0.01	#N/A	New DEG
mtr	3.98	7.06E-10	-2.55	Opposite
uxaA	3.94	7.01E-11	#N/A	New DEG
Z1062	3.91	3.26E-07	-2.73	Opposite
Z1930	3.83	3.99E-17	-2.34	Opposite
Z2283	3.82	0.01	#N/A	New DEG
Z4018	3.82	1.32E-04	-4.08	Opposite
cydA	3.81	4.70E-17	-1.83	Opposite
cstC	3.78	7.79E-15	-7.93	Opposite
ygjG	3.78	8.00E-15	-10.79	Opposite
yeaQ	3.77	3.03E-06	-3.75	Opposite
yehY	3.72	3.36E-03	#N/A	New DEG
talA	3.72	3.87E-12	-2.36	Opposite
narY	3.68	0.04	#N/A	New DEG
ybaA	3.66	0.04	#N/A	New DEG
cydB	3.65	1.02E-15	#N/A	New DEG
yeiQ	3.65	1.15E-10	#N/A	New DEG
rmf	3.65	1.75E-16	-2.43	Opposite
Z2593	3.63	6.25E-03	#N/A	New DEG
csgF	3.6	9.46E-09	-3.13	Opposite
yciR	3.52	2.94E-07	#N/A	New DEG
Z2883	3.48	7.47E-05	-2.69	Opposite
uxuB	3.47	3.01E-09	#N/A	New DEG
yeaG	3.46	4.66E-13	-4.9	Opposite
otsA	3.45	1.40E-10	-3.12	Opposite
Z3045	3.44	2.25E-07	-2.83	Opposite
yddB	3.4	5.66E-13	-2.95	Opposite
Z2379	3.35	1.17E-03	-3.28	Opposite

fruA	3.34	1.30E-10	-2.65	Opposite
ybhP	3.32	0.03	#N/A	New DEG
yedU	3.31	5.34E-13	-2.48	Opposite
fruK	3.29	6.80E-09	-2.31	Opposite
yhdX	3.27	1.75E-06	-9.9	Opposite
Z2421	3.26	1.66E-05	#N/A	New DEG
Z1211	3.25	5.35E-12	1.97	Further up
ycgB	3.2	9.04E-06	-3.08	Opposite
Z1931	3.19	1.95E-13	-2.14	Opposite
Z2224	3.16	8.44E-03	-18.24	Opposite
csgE	3.15	1.61E-08	-2.99	Opposite
Z1972	3.14	3.33E-09	-2.56	Opposite
Z1925	3.07	1.59E-08	-2.38	Opposite
yhdZ	3.06	6.16E-07	-7.99	Opposite
ybaS	3.02	2.67E-05	#N/A	New DEG
Z1651	3.01	1.24E-10	2.66	Further up
nrdD	3	2.18E-08	#N/A	New DEG
Z5978	2.98	1.02E-04	-3.43	Opposite
Z2815	2.96	0.01	#N/A	New DEG
yhcJ	2.95	0.04	#N/A	New DEG
wrbA	2.94	1.54E-06	-2.09	Opposite
Z3074	2.94	4.57E-03	-3.19	Opposite
yebA	2.93	2.77E-09	-2.07	Opposite
ybjM	2.92	6.10E-03	#N/A	New DEG
yiaG	2.92	6.00E-04	#N/A	New DEG
argT	2.9	5.68E-11	-5.58	Opposite
yddA	2.88	1.73E-08	-2.39	Opposite
yeaH	2.85	3.94E-07	-3.4	Opposite
ydfJ	2.84	0.05	#N/A	New DEG
osmY	2.84	3.33E-08	-2.98	Opposite
yhjY	2.83	6.00E-04	#N/A	New DEG
uxaB	2.83	3.25E-04	#N/A	New DEG
rpsV	2.81	1.87E-06	-2.33	Opposite
Z2695	2.8	1.59E-03	#N/A	New DEG
Z2220	2.79	6.91E-03	#N/A	New DEG
yccJ	2.79	2.07E-06	-2.1	Opposite
adhE	2.78	1.52E-10	#N/A	New DEG
chaC	2.78	2.23E-08	-3.14	Opposite
pqqL	2.77	2.55E-09	-3.07	Opposite
yciF	2.77	5.80E-03	-4.01	Opposite
ybdK	2.75	7.93E-03	#N/A	New DEG
Z3260	2.74	1.08E-08	-2.36	Opposite
csgD	2.74	9.07E-09	-2.41	Opposite

Z1971	2.73	2.36E-03	#N/A	New DEG
ybaY	2.72	7.94E-08	-2.35	Opposite
glnP	2.71	3.57E-07	-3	Opposite
ybgE	2.69	2.26E-07	#N/A	New DEG
Z2066	2.68	0.04	#N/A	New DEG
Z2428	2.66	0.05	-6.83	Opposite
ygbD	2.65	3.14E-03	#N/A	New DEG
Z2287	2.65	6.97E-06	-1.97	Opposite
Z2301	2.64	3.85E-03	#N/A	New DEG
ydbL	2.63	0.03	#N/A	New DEG
Z2585	2.62	2.26E-04	#N/A	New DEG
nanA	2.62	1.43E-03	#N/A	New DEG
Z1653	2.6	2.68E-04	#N/A	New DEG
csgG	2.6	9.17E-07	-2.46	Opposite
yehV	2.59	0.03	#N/A	New DEG
Z4353	2.56	4.67E-03	#N/A	New DEG
Z2809	2.54	0.02	#N/A	New DEG
yhjG	2.54	1.20E-03	#N/A	New DEG
Z1056	2.54	1.55E-03	-2.64	Opposite
yohC	2.54	0.05	-2.81	Opposite
ycaC	2.53	4.07E-03	#N/A	New DEG
glnH	2.53	6.90E-08	-2.63	Opposite
himA	2.5	1.95E-08	#N/A	New DEG
ndh	2.49	1.46E-07	#N/A	New DEG
ybgA	2.49	0.02	#N/A	New DEG
ecnB	2.46	3.17E-04	-2.36	Opposite
yhdY	2.44	0.01	-12.48	Opposite
Z2290	2.43	0.04	#N/A	New DEG
bax	2.43	1.44E-07	-1.9	Opposite
Z1921	2.41	7.48E-04	-2.4	Opposite
Z2275	2.39	5.29E-04	-4.16	Opposite
yqjG	2.37	6.96E-03	#N/A	New DEG
amyA	2.37	1.95E-04	#N/A	New DEG
Z2054	2.37	2.81E-07	-1.95	Opposite
Z2251	2.33	0.03	#N/A	New DEG
glnQ	2.33	1.07E-05	-2.37	Opposite
Z1652	2.31	2.61E-05	#N/A	New DEG
yhjD	2.31	4.48E-04	#N/A	New DEG
proV	2.3	7.84E-06	#N/A	New DEG
Z2811	2.29	0.04	#N/A	New DEG
ypjA	2.28	9.61E-05	#N/A	New DEG
ygaE	2.27	3.45E-04	-3.41	Opposite
epd	2.26	2.66E-06	#N/A	New DEG

sodC	2.25	0.04	#N/A	New DEG
Z2063	2.25	4.60E-05	#N/A	New DEG
yeaD	2.25	4.72E-06	-1.97	Opposite
yrbL	2.23	6.34E-05	#N/A	New DEG
yceP	2.22	6.18E-03	#N/A	New DEG
Z2386	2.2	3.59E-06	#N/A	New DEG
cls	2.17	6.04E-05	-1.91	Opposite
chaA	2.16	3.82E-05	#N/A	New DEG
Z2387	2.14	2.45E-05	#N/A	New DEG
malG	2.13	0.03	#N/A	New DEG
proW	2.13	6.99E-04	#N/A	New DEG
Z6054	2.13	0.04	-2.4	Opposite
add	2.12	2.51E-04	#N/A	New DEG
trg	2.1	0.04	#N/A	New DEG
osmE	2.1	5.39E-04	-1.95	Opposite
yhgG	2.09	8.26E-03	#N/A	New DEG
Z1654	2.09	6.25E-03	#N/A	New DEG
ytfK	2.09	1.07E-03	#N/A	New DEG
ycjZ	2.09	0.03	#N/A	New DEG
proX	2.08	3.65E-04	#N/A	New DEG
yggB	2.08	2.76E-05	-2.3	Opposite
ydcH	2.06	1.58E-03	#N/A	New DEG
ygaU	2.05	8.23E-04	#N/A	New DEG
ompC	2.05	1.89E-05	#N/A	New DEG
yjjV	2.04	0.02	#N/A	New DEG
xasA	2.02	7.10E-05	-2.12	Opposite
Z2040	2.01	5.04E-03	#N/A	New DEG
ptsH	2	5.75E-05	#N/A	New DEG
ygfP	2	5.77E-03	-4.58	Opposite
codB	2	9.13E-04	-8.45	Opposite
Z1212	1.98	1.12E-03	#N/A	New DEG
yjbA	1.98	9.30E-03	-2.97	Opposite
rcsA	1.98	4.57E-03	-5.87	Opposite
yifA	1.97	2.31E-03	#N/A	New DEG
malF	1.96	5.29E-03	#N/A	New DEG
gadB	1.96	1.51E-04	-2.02	Opposite
yiiO	1.95	3.37E-04	#N/A	New DEG
yhjC	1.95	0.01	#N/A	New DEG
Z1957	1.95	0.02	#N/A	New DEG
ycfS	1.95	2.36E-03	#N/A	New DEG
ada	1.94	0.03	#N/A	New DEG
dacC	1.94	6.90E-04	#N/A	New DEG
ycjG	1.94	0.01	#N/A	New DEG

ydeK	1.94	4.08E-03	#N/A	New DEG
sdiA	1.94	0.01	#N/A	New DEG
Z2219	1.93	0.02	#N/A	New DEG
cspD	1.93	2.62E-04	#N/A	New DEG
Z2592	1.92	0.01	#N/A	New DEG
ybdG	1.92	2.74E-03	#N/A	New DEG
grxB	1.91	5.78E-04	#N/A	New DEG
pyrI	1.9	3.08E-03	#N/A	New DEG
Z1213	1.89	0.04	#N/A	New DEG
ycjF	1.89	0.02	#N/A	New DEG
ynfC	1.89	0.02	#N/A	New DEG
ydfH	1.87	9.22E-03	#N/A	New DEG
ymcD	1.87	3.45E-04	#N/A	New DEG
osmB	1.86	0.03	#N/A	New DEG
yebM	1.86	8.27E-03	#N/A	New DEG
rstB	1.85	0.03	#N/A	New DEG
yajF	1.85	0.05	#N/A	New DEG
fdhF	1.85	8.46E-03	-2.34	Opposite
Z1052	1.85	0.01	-2.61	Opposite
focA	1.83	2.78E-03	#N/A	New DEG
qseA	1.83	0.05	#N/A	New DEG
feoB	1.82	1.67E-03	#N/A	New DEG
ycbW	1.82	3.33E-03	#N/A	New DEG
Z3077	1.81	0.05	#N/A	New DEG
hdhA	1.81	1.87E-03	#N/A	New DEG
yhaH	1.81	0.02	#N/A	New DEG
ycgN	1.8	0.02	#N/A	New DEG
ydbD	1.8	0.04	#N/A	New DEG
cydC	1.8	3.44E-03	#N/A	New DEG
ydgA	1.8	2.12E-03	#N/A	New DEG
malE	1.79	4.11E-03	#N/A	New DEG
yjjU	1.79	0.04	-2.43	Opposite
ydeA	1.78	0.04	#N/A	New DEG
gapA	1.78	1.12E-03	#N/A	New DEG
htpX	1.78	2.23E-03	#N/A	New DEG
pykF	1.77	1.84E-03	#N/A	New DEG
dsbB	1.77	0.02	#N/A	New DEG
yciS	1.77	0.02	#N/A	New DEG
malK	1.77	0.05	#N/A	New DEG
aldA	1.76	3.05E-03	#N/A	New DEG
'yeaJ	1.75	0.03	#N/A	New DEG
ptsI	1.75	1.81E-03	#N/A	New DEG
yqjE	1.74	0.01	#N/A	New DEG

gapC	1.73	0.05	#N/A	New DEG
Z3768	1.72	0.03	#N/A	New DEG
yceF	1.72	0.02	#N/A	New DEG
pntA	1.72	3.02E-03	#N/A	New DEG
Z2024	1.71	0.04	#N/A	New DEG
yoaE	1.71	8.27E-03	#N/A	New DEG
pgpB	1.7	0.05	#N/A	New DEG
yadC	1.7	0.02	#N/A	New DEG
cysQ	1.69	0.02	#N/A	New DEG
Z2755	1.69	0.05	#N/A	New DEG
Z0609	1.69	0.03	#N/A	New DEG
potB	1.68	0.05	#N/A	New DEG
Z2099	1.67	6.36E-03	#N/A	New DEG
mlc	1.67	0.02	#N/A	New DEG
osmC	1.66	9.76E-03	#N/A	New DEG
ytfE	1.66	9.66E-03	#N/A	New DEG
aceA	1.65	0.04	#N/A	New DEG
Z0371	1.65	0.04	#N/A	New DEG
Z2090	1.64	0.05	#N/A	New DEG
Z1932	1.64	0.03	#N/A	New DEG
rpoS	1.64	8.38E-03	#N/A	New DEG
codA	1.64	0.02	-4.9	Opposite
yceI	1.63	0.02	#N/A	New DEG
manX	1.63	0.01	#N/A	New DEG
Z6021	1.62	0.03	#N/A	New DEG
ybaR	1.62	0.02	#N/A	New DEG
manZ	1.62	0.01	#N/A	New DEG
lamB	1.62	0.04	#N/A	New DEG
potA	1.61	0.04	#N/A	New DEG
ydcG	1.6	0.03	#N/A	New DEG
yccA	1.6	0.02	#N/A	New DEG
xseA	1.6	0.03	#N/A	New DEG
malQ	1.6	0.04	#N/A	New DEG
Z3095	1.6	0.05	#N/A	New DEG
pntB	1.59	0.02	#N/A	New DEG
speD	1.59	0.03	#N/A	New DEG
pepT	1.59	0.05	#N/A	New DEG
metK	1.59	0.02	-2.03	Opposite
metE	1.59	0.02	-5.17	Opposite
nadE	1.58	0.03	#N/A	New DEG
aroG	1.58	0.02	#N/A	New DEG
dcp	1.58	0.03	#N/A	New DEG
solA	1.57	0.04	#N/A	New DEG

Z4134	1.57	0.05	#N/A	New DEG
Z1600	1.56	0.04	#N/A	New DEG
Z3603	1.54	0.04	#N/A	New DEG
Z1942	1.54	0.05	#N/A	New DEG
ydiC	1.54	0.04	#N/A	New DEG
dcuA	1.53	0.04	#N/A	New DEG
p0061+p0062	1.53	0.03	#N/A	New DEG
rseA	1.53	0.04	-2.05	Opposite
Z1153	1.52	0.05	#N/A	New DEG
nlpD	1.51	0.04	#N/A	New DEG
Z3198	-1.5	0.05	#N/A	New DEG
atpB	-1.5	0.05	#N/A	New DEG
cspE	-1.51	0.05	#N/A	New DEG
icdA	-1.51	0.04	#N/A	New DEG
acnB	-1.52	0.04	#N/A	New DEG
Z5140	-1.53	0.05	#N/A	New DEG
secB	-1.53	0.05	#N/A	New DEG
wbdQ	-1.53	0.05	#N/A	New DEG
ycdB	-1.53	0.04	#N/A	New DEG
manC	-1.54	0.03	#N/A	New DEG
recR	-1.55	0.05	#N/A	New DEG
ruvB	-1.56	0.04	#N/A	New DEG
tatA	-1.56	0.04	#N/A	New DEG
yigR	-1.56	0.05	#N/A	New DEG
purN	-1.57	0.03	#N/A	New DEG
ompX	-1.57	0.02	#N/A	New DEG
atpF	-1.57	0.02	#N/A	New DEG
hscA	-1.57	0.03	#N/A	New DEG
Z1186	-1.57	0.05	1.91	Opposite
gmk	-1.58	0.04	#N/A	New DEG
fabF	-1.58	0.02	#N/A	New DEG
Z5347	-1.59	0.04	#N/A	New DEG
gor	-1.59	0.04	#N/A	New DEG
Z4386	-1.59	0.02	1.83	Opposite
eae	-1.59	0.02	-2.03	Further down
ycaJ	-1.6	0.04	#N/A	New DEG
nusA	-1.6	0.02	#N/A	New DEG
hemE	-1.6	0.03	#N/A	New DEG
Z4919	-1.6	0.03	#N/A	New DEG
dppD	-1.61	0.04	#N/A	New DEG
ubiE	-1.62	0.03	#N/A	New DEG
gcd	-1.62	0.03	#N/A	New DEG
serA	-1.62	0.01	2.22	Opposite

Z1026	-1.63	0.02	#N/A	New DEG
ilvE	-1.63	0.02	#N/A	New DEG
leuC	-1.63	0.03	#N/A	New DEG
parE	-1.64	0.02	#N/A	New DEG
rbfA	-1.64	0.04	#N/A	New DEG
yagU	-1.64	0.02	#N/A	New DEG
yggX	-1.64	0.05	#N/A	New DEG
Z0955	-1.64	0.01	-2.08	Further down
fis	-1.65	0.02	#N/A	New DEG
hemB	-1.65	0.02	#N/A	New DEG
yigZ	-1.65	0.05	#N/A	New DEG
yidA	-1.65	0.02	#N/A	New DEG
deoC	-1.65	0.05	#N/A	New DEG
yjeR	-1.65	0.05	#N/A	New DEG
Z1625	-1.65	0.02	#N/A	New DEG
infB	-1.66	6.53E-03	#N/A	New DEG
truB	-1.66	0.02	#N/A	New DEG
selB	-1.66	0.02	#N/A	New DEG
polB	-1.67	0.05	#N/A	New DEG
wecE	-1.68	0.03	#N/A	New DEG
waaY	-1.68	0.05	#N/A	New DEG
aroE	-1.68	0.03	#N/A	New DEG
pnuC	-1.68	0.01	#N/A	New DEG
nuoF	-1.69	0.01	#N/A	New DEG
yhbC	-1.69	0.02	#N/A	New DEG
hslU	-1.7	6.70E-03	#N/A	New DEG
dfp	-1.7	0.01	#N/A	New DEG
atpG	-1.71	3.08E-03	#N/A	New DEG
sun	-1.71	0.01	#N/A	New DEG
gsp	-1.71	6.65E-03	#N/A	New DEG
Z0516	-1.71	0.01	#N/A	New DEG
yafB	-1.71	0.04	2.18	Opposite
ruvA	-1.71	0.01	1.84	Opposite
Z5139	-1.72	0.01	#N/A	New DEG
gnd	-1.72	3.17E-03	#N/A	New DEG
betA	-1.72	7.92E-03	#N/A	New DEG
atpC	-1.72	3.14E-03	#N/A	New DEG
Z5899	-1.72	0.01	#N/A	New DEG
mglC	-1.72	0.05	2.24	Opposite
betI	-1.73	0.01	#N/A	New DEG
aroF	-1.73	0.05	#N/A	New DEG
atpH	-1.74	2.29E-03	#N/A	New DEG
selA	-1.74	0.01	#N/A	New DEG

nuoI	-1.74	0.04	#N/A	New DEG
lipA	-1.74	3.87E-03	#N/A	New DEG
hemG	-1.75	0.02	#N/A	New DEG
thrB	-1.75	3.51E-03	#N/A	New DEG
Z5113	-1.76	2.92E-03	#N/A	New DEG
purA	-1.76	1.90E-03	#N/A	New DEG
lysU	-1.76	0.01	#N/A	New DEG
thrA	-1.76	1.88E-03	#N/A	New DEG
atpA	-1.77	1.36E-03	#N/A	New DEG
atpD	-1.77	1.29E-03	#N/A	New DEG
yigM	-1.77	0.02	#N/A	New DEG
dnaT	-1.77	0.03	#N/A	New DEG
gltP	-1.78	6.55E-03	#N/A	New DEG
Z5115	-1.8	2.54E-03	#N/A	New DEG
tatC	-1.8	4.75E-03	#N/A	New DEG
thdF	-1.8	5.84E-03	#N/A	New DEG
rbsK	-1.8	0.02	#N/A	New DEG
nadB	-1.8	2.18E-03	#N/A	New DEG
mglA	-1.8	0.01	2.53	Opposite
sucD	-1.8	1.12E-03	2.05	Opposite
yihA	-1.81	8.24E-03	#N/A	New DEG
Z4000	-1.81	0.05	#N/A	New DEG
rph	-1.81	0.01	#N/A	New DEG
betB	-1.82	2.66E-03	#N/A	New DEG
ytfL	-1.82	8.27E-03	#N/A	New DEG
yieP	-1.82	0.03	#N/A	New DEG
nuoM	-1.83	3.93E-03	#N/A	New DEG
fimB	-1.83	0.01	2.43	Opposite
zntA	-1.84	0.05	#N/A	New DEG
Z3069	-1.84	0.05	#N/A	New DEG
dnaC	-1.85	0.01	#N/A	New DEG
thrC	-1.85	9.06E-04	#N/A	New DEG
kbl	-1.85	6.90E-04	2.23	Opposite
tdh	-1.85	7.74E-04	2.11	Opposite
Z5129	-1.86	8.23E-04	#N/A	New DEG
udhA	-1.86	6.43E-04	#N/A	New DEG
yigI	-1.87	0.02	#N/A	New DEG
Z0722	-1.87	0.04	2.19	Opposite
sepZ	-1.88	4.24E-04	#N/A	New DEG
mdaB	-1.88	6.25E-03	#N/A	New DEG
murI	-1.88	3.75E-03	1.91	Opposite
nrdB	-1.89	4.54E-04	#N/A	New DEG
btuB	-1.89	4.92E-04	#N/A	New DEG

yecO	-1.89	9.92E-03	#N/A	New DEG
sbmC	-1.89	0.04	2.41	Opposite
gltD	-1.89	3.85E-04	2.05	Opposite
pepQ	-1.9	4.22E-04	#N/A	New DEG
yhjE	-1.9	1.02E-03	#N/A	New DEG
nadA	-1.9	5.61E-04	#N/A	New DEG
Z0879	-1.9	0.02	2.1	Opposite
atpE	-1.91	1.90E-04	#N/A	New DEG
sucC	-1.92	1.96E-04	2	Opposite
Z4650	-1.93	2.36E-03	#N/A	New DEG
recF	-1.94	5.73E-03	#N/A	New DEG
gidA	-1.94	2.45E-04	2.08	Opposite
cyoE	-1.95	1.59E-04	#N/A	New DEG
yjgD	-1.95	2.75E-04	2.01	Opposite
nrdA	-1.96	1.18E-04	#N/A	New DEG
greA	-1.96	2.97E-03	#N/A	New DEG
galE	-1.98	3.98E-03	#N/A	New DEG
Z1579	-1.98	7.99E-04	2.13	Opposite
cvpA	-1.99	1.51E-04	#N/A	New DEG
cyoC	-1.99	1.40E-04	#N/A	New DEG
hisB	-2	1.77E-04	#N/A	New DEG
gltB	-2	4.78E-05	2.51	Opposite
Z5128	-2.01	3.66E-04	#N/A	New DEG
corA	-2.01	4.44E-04	#N/A	New DEG
evgA	-2.02	2.80E-03	#N/A	New DEG
ycdO	-2.02	4.46E-05	#N/A	New DEG
ycaD	-2.03	0.01	#N/A	New DEG
Z1020	-2.03	1.30E-03	2.03	Opposite
purF	-2.04	3.35E-05	#N/A	New DEG
Z4846	-2.05	3.17E-04	#N/A	New DEG
yfhJ	-2.05	2.42E-03	#N/A	New DEG
Z0347	-2.05	0.05	#N/A	New DEG
rnk	-2.06	0.01	#N/A	New DEG
gidB	-2.07	1.54E-03	1.95	Opposite
sdhC	-2.07	4.44E-04	1.86	Opposite
ycdQ	-2.08	0.05	#N/A	New DEG
fadA	-2.09	8.80E-03	#N/A	New DEG
hisF	-2.1	1.24E-04	#N/A	New DEG
fadL	-2.11	4.20E-04	2.15	Opposite
sseB	-2.13	7.82E-05	#N/A	New DEG
hisH	-2.13	2.25E-04	#N/A	New DEG
lysC	-2.15	3.10E-05	#N/A	New DEG
acs	-2.15	1.20E-04	2.68	Opposite

tatD	-2.15	6.48E-03	2.24	Opposite
cyoD	-2.16	2.93E-05	#N/A	New DEG
pepB	-2.16	1.29E-05	#N/A	New DEG
ppsA	-2.17	3.58E-06	#N/A	New DEG
hisI	-2.2	2.08E-05	#N/A	New DEG
sdhD	-2.2	1.35E-04	2.47	Opposite
Z1057	-2.2	3.72E-03	2.2	Opposite
sucB	-2.2	2.92E-06	2	Opposite
Z2751	-2.21	5.51E-03	2.1	Opposite
Z3109	-2.23	0.02	2.92	Opposite
Z2771	-2.23	7.20E-04	2.32	Opposite
sdhB	-2.23	1.13E-05	2	Opposite
yfaE	-2.24	1.58E-03	#N/A	New DEG
yhjOP	-2.24	0.04	#N/A	New DEG
cyoB	-2.25	9.72E-07	#N/A	New DEG
yeeF	-2.25	3.20E-05	#N/A	New DEG
Z3107	-2.25	0.03	#N/A	New DEG
pckA	-2.25	1.46E-06	2.37	Opposite
pta	-2.25	8.44E-07	1.94	Opposite
deaD	-2.26	1.38E-06	2.22	Opposite
ycdR	-2.27	0.01	#N/A	New DEG
htrL	-2.27	1.92E-05	2.39	Opposite
cyoA	-2.28	7.72E-07	#N/A	New DEG
emrA	-2.28	2.41E-03	#N/A	New DEG
yafN	-2.28	0.04	#N/A	New DEG
yjfR	-2.28	0.05	2.62	Opposite
uvrB	-2.29	1.47E-06	2.35	Opposite
exoU	-2.3	0.03	#N/A	New DEG
fadB	-2.31	1.40E-04	2.22	Opposite
sucA	-2.31	3.55E-07	1.97	Opposite
yceL	-2.33	0.03	#N/A	New DEG
Z0067	-2.34	1.68E-05	2.01	Opposite
uvrD	-2.36	1.41E-06	2.5	Opposite
sodA	-2.39	9.01E-08	1.83	Opposite
Z3272	-2.41	1.68E-04	2.06	Opposite
hisA	-2.42	4.04E-06	#N/A	New DEG
Z6024	-2.43	4.09E-05	-1.95	Further down
asnA	-2.44	3.87E-08	2.35	Opposite
Z1341	-2.45	6.63E-08	2.08	Opposite
fbp	-2.46	2.67E-07	#N/A	New DEG
pstB	-2.47	1.03E-04	2.4	Opposite
yafO	-2.52	0.02	#N/A	New DEG
ylaC	-2.52	1.61E-03	#N/A	New DEG

nikA	-2.53	0.01	#N/A	New DEG
yhcG	-2.53	0.01	#N/A	New DEG
soxS	-2.56	7.82E-05	2.5	Opposite
p0046	-2.57	3.53E-10	2.2	Opposite
rarD	-2.6	3.21E-03	#N/A	New DEG
sdhA	-2.6	1.35E-08	1.98	Opposite
thiC	-2.63	0.03	#N/A	New DEG
p0045	-2.63	2.93E-05	2.46	Opposite
ackA	-2.68	1.01E-09	2.03	Opposite
Z3140	-2.72	1.59E-08	#N/A	New DEG
cspA	-2.75	3.45E-05	3.18	Opposite
uvrA	-2.75	1.53E-09	2.74	Opposite
hisC	-2.76	8.66E-08	#N/A	New DEG
aldH	-2.8	0.01	3.84	Opposite
yigN	-2.81	6.13E-06	2.92	Opposite
Z2984	-2.82	3.29E-04	#N/A	New DEG
asnC	-2.82	0.05	#N/A	New DEG
glyA	-2.9	1.86E-11	2.26	Opposite
cycA	-2.9	1.71E-10	-2.38	Further down
Z1838	-2.91	0.02	#N/A	New DEG
Z4708	-2.91	5.50E-03	#N/A	New DEG
hisD	-2.93	4.05E-09	2.2	Opposite
Z3271	-2.98	9.72E-07	2.65	Opposite
hisG	-2.98	3.01E-09	2.48	Opposite
Z3970	-3.09	2.07E-03	2.57	Opposite
yebF	-3.19	1.07E-09	2.66	Opposite
purD	-3.24	2.80E-13	2.19	Opposite
ilvB	-3.27	2.09E-09	3.5	Opposite
ilvN	-3.3	2.73E-03	2.57	Opposite
yebG	-3.49	3.02E-12	3.14	Opposite
purH	-3.54	0	2.38	Opposite
ycjL	-3.61	9.76E-05	2.92	Opposite
dinP	-3.83	5.50E-11	3.91	Opposite
ordL	-3.84	1.95E-03	2.94	Opposite
yjgH	-3.99	0.03	#N/A	New DEG
tnaA	-4	1.31E-07	5.72	Opposite
lexA	-4.03	8.05E-17	3.8	Opposite
gcvP	-4.25	2.18E-18	#N/A	New DEG
Z5163	-4.29	0.04	#N/A	New DEG
gcvH	-4.3	6.86E-13	#N/A	New DEG
Z2980	-4.37	1.29E-03	6.05	Opposite
gcvT	-4.52	1.81E-18	2	Opposite
dinI	-4.54	6.45E-13	4.89	Opposite

yjcH	-4.91	5.43E-03	4.29	Opposite
Z2491	-4.99	4.24E-10	2.53	Opposite
coxT	-5.14	7.12E-12	2.36	Opposite
yfhL	-5.53	0.05	#N/A	New DEG
Z4171	-5.56	0.04	#N/A	New DEG
yojH	-5.85	5.40E-26	1.88	Opposite
Z5214	-6.02	9.38E-03	#N/A	New DEG
dinD	-6.28	1.05E-15	7.24	Opposite
ygcY	-6.53	2.23E-12	10.5	Opposite
dinF	-6.84	7.22E-20	3.98	Opposite
ydiF	-7.2	6.18E-03	#N/A	New DEG
Z4104	-7.32	7.56E-08	17.02	Opposite
umuC	-7.43	5.29E-23	4.92	Opposite
recA	-7.46	0	5.46	Opposite
yaeG	-7.53	3.78E-23	9.1	Opposite
Z0315	-7.77	2.85E-10	6.49	Opposite
ybdJ	-7.96	0.04	#N/A	New DEG
Z2972	-7.98	7.21E-18	3.88	Opposite
sulA	-8.12	6.79E-36	6.25	Opposite
Z2757	-8.53	5.24E-03	#N/A	New DEG
Z2976	-9.2	2.99E-07	5.3	Opposite
Z2971	-9.69	5.05E-31	4.41	Opposite
Z0314	-9.74	2.77E-17	10.06	Opposite
Z0316	-9.84	2.55E-22	7.17	Opposite
oraA	-9.96	1.71E-10	5.16	Opposite
ygcX	-11.49	1.94E-25	15.2	Opposite
umuD	-11.49	1.80E-20	6.64	Opposite
Z2973	-12.8	1.16E-30	4.89	Opposite
yhaG	-13.43	4.47E-29	12.27	Opposite
recN	-14.08	2.60E-57	8.5	Opposite
Z0311	-14.68	1.34E-23	14.4	Opposite
Z0313	-14.82	2.13E-16	12.96	Opposite
Z0317	-20.13	1.22E-26	8.62	Opposite
Z2979	-20.14	9.26E-50	21.36	Opposite
Z2975	-20.16	1.25E-17	14.04	Opposite
intH	-20.32	4.03E-86	12.17	Opposite
Z0312	-24.45	4.27E-18	20.34	Opposite
Z2978	-29.08	1.28E-50	17.36	Opposite
yhaD	-29.65	6.94E-83	41.23	Opposite
Z4888	-31.96	9.30E-03	#N/A	New DEG
Z2974	-31.98	5.89E-70	16.85	Opposite
Z0310	-35.4	7.65E-21	61.39	Opposite
Z2977	-35.78	2.60E-25	43.64	Opposite

Z4105	-74.86	7.33E-56	35.2	Opposite
ygjU	-87.68	1.86E-107	#N/A	New DEG
yhaF	-92.93	2.27E-78	183.86	Opposite
yhaE	-125.07	1.37E-117	121.04	Opposite
yhaU	-302.08	1.31E-71	75.48	Opposite

Table S3. Genes differentially expressed comparing EHEC WT D-ser with LCV2 D-ser by RNA-seq and comparison of directionality with WT D-ser

Feature ID	Fold change WT D-ser vs 2 D-ser	FDR corrected p-value	Fold Change WT vs WT D-ser	Direction of expression in WT D-ser vs LCV2 D-ser
Z3065	276.87	1.52E-179	-7.88	Opposite
Z3624	155.74	2.89E-89	#N/A	New DEG
Z3066	68.77	1.36E-05	#N/A	New DEG
Z3627	62.21	1.88E-39	#N/A	New DEG
Z3625	58.29	2.66E-122	#N/A	New DEG
yebL	36.73	2.84E-121	-4.55	Opposite
ykgM	36.08	3.06E-48	#N/A	New DEG
dsdA	33.05	1.34E-108	#N/A	New DEG
Z3623	28.24	2.58E-67	#N/A	New DEG
csgB	22.85	1.71E-59	-25.92	Opposite
csgA	18.26	2.73E-67	-19.54	Opposite
Z1924	14.28	0.000000268	-28.21	Opposite
Z1923	10.26	0.01	-39.84	Opposite
yebA	10.14	4.91E-49	-2.07	Opposite
uxaC	7.81	3.26E-27	#N/A	New DEG
dps	7.73	1.59E-49	-2.9	Opposite
Z3956	7.45	5.06E-18	#N/A	New DEG
Z3249	7.45	1.35E-17	-5.01	Opposite
Z6041	6.78	8.09E-03	#N/A	New DEG
ygaM	6.3	2.09E-12	-3.9	Opposite
terF	5.96	2.00E-02	#N/A	New DEG
gabD	5.69	8.64E-18	-11.56	Opposite
csgC	5.61	0.01	-6.94	Opposite
adhP	5.6	7.19E-15	-4.3	Opposite
uxaA	5.56	2.92E-18	#N/A	New DEG
Z1507	5.51	0.000000911	-34.17	Opposite
yebI	5.46	2.92E-18	#N/A	New DEG
gabT	5.38	5.02E-15	-12.83	Opposite
poxB	5.32	5.91E-21	-3.31	Opposite
otsB	5.31	8.03E-14	-3.78	Opposite
pyrL	5.14	5.00E-02	#N/A	New DEG
Z3043	5.07	3.36E-09	-2.61	Opposite

yedL	5.03	5.48E-20	-12.28	Opposite
uxuA	4.98	1.31E-17	#N/A	New DEG
ycdG	4.94	5.97E-13	-25.41	Opposite
fruB	4.93	1.36E-14	-3.01	Opposite
gabP	4.86	1.68E-09	-9.67	Opposite
ygaF	4.85	1.36E-10	#N/A	New DEG
Z2269	4.84	2.77E-08	-4.12	Opposite
yahO	4.8	5.25E-07	-3.56	Opposite
yebM	4.78	1.32E-18	#N/A	New DEG
elaB	4.78	2.74E-18	-2.84	Opposite
glnK	4.72	5.56E-22	-9.63	Opposite
Z1511	4.66	9.93E-11	-34.52	Opposite
Z2469	4.61	0.05	#N/A	New DEG
Z5978	4.59	3.90E-10	-3.43	Opposite
tktB	4.57	1.07E-19	-3.17	Opposite
Z0923	4.54	5.68E-09	-3.15	Opposite
Z3045	4.52	1.42E-11	-2.83	Opposite
yeaQ	4.51	1.63E-08	-3.75	Opposite
phnB	4.47	4.52E-04	#N/A	New DEG
amtB	4.39	1.24E-22	-9.31	Opposite
yeiQ	4.37	1.14E-14	#N/A	New DEG
Z2203	4.3	8.57E-03	#N/A	New DEG
katE	4.3	8.51E-15	-4.45	Opposite
csgF	4.22	9.48E-12	-3.13	Opposite
yehV	4.21	8.79E-06	#N/A	New DEG
Z2224	4.21	0.0000611	-18.24	Opposite
Z1510	4.2	0.00000334	-33.6	Opposite
ygjG	4.12	7.4E-18	-10.79	Opposite
rmf	4.11	4.50E-21	-2.43	Opposite
Z2223	4.11	8.48E-14	-23.54	Opposite
yeaG	4.07	9.56E-18	-4.9	Opposite
ycgB	4.04	5.69E-09	-3.08	Opposite
ybaY	3.95	9.38E-16	-2.35	Opposite
Z2779	3.94	4.59E-15	-9.65	Opposite
Z2222	3.93	0.000000161	-27.06	Opposite
otsA	3.88	1.93E-13	-3.12	Opposite
ydaK	3.88	2.59E-08	-5.58	Opposite
ydjS	3.84	8.83E-14	-7.47	Opposite
osmY	3.83	1.84E-14	-2.98	Opposite
yohC	3.8	2.61E-04	-2.81	Opposite
Z2427	3.73	0.0000831	-27.36	Opposite
Z1925	3.7	2.81E-12	-2.38	Opposite
talA	3.66	1.76E-12	-2.36	Opposite

ybaS	3.63	1.26E-07	#N/A	New DEG
Z2287	3.63	4.51E-11	-1.97	Opposite
csgE	3.58	3.71E-11	-2.99	Opposite
uxaB	3.54	0.000000838	#N/A	New DEG
Z2778	3.51	1.15E-13	-8.25	Opposite
nrdD	3.49	2.10E-11	#N/A	New DEG
ybgA	3.46	6.11E-05	#N/A	New DEG
wrbA	3.4	7.56E-09	-2.09	Opposite
Z2777	3.4	6.82E-13	-6.73	Opposite
uxuB	3.39	2.31E-09	#N/A	New DEG
Z2421	3.3	0.00000681	#N/A	New DEG
fruK	3.29	2.06E-09	-2.31	Opposite
fruA	3.29	5.65E-11	-2.65	Opposite
Z1930	3.28	1.26E-14	-2.34	Opposite
Z1678	3.27	5.00E-02	-3.89	Opposite
csgD	3.25	6.59E-13	-2.41	Opposite
yddB	3.25	6.59E-13	-2.95	Opposite
yedU	3.22	1.55E-13	-2.48	Opposite
cstC	3.22	2.39E-12	-7.93	Opposite
Z1178	3.21	7.31E-15	-1.8	Opposite
yciF	3.2	0.0006	-4.01	Opposite
Z1617	3.15	1.97E-14	-1.8	Opposite
ytfE	3.14	1.63E-13	#N/A	New DEG
cydA	3.1	1.46E-13	-1.83	Opposite
Z1509	3.08	0.00681	-17.77	Opposite
yciR	3.07	8.44E-06	#N/A	New DEG
Z3252	3.07	3.54E-03	#N/A	New DEG
nrdG	3.06	0.03	#N/A	New DEG
adhE	3.05	1.38E-13	#N/A	New DEG
xasA	3.05	8.12E-13	-2.12	Opposite
Z1062	3.04	1.76E-04	-2.73	Opposite
yehY	3.03	2.00E-02	#N/A	New DEG
ecnB	3.03	1.15E-06	-2.36	Opposite
ygfO	3.02	2.00E-02	#N/A	New DEG
rpsV	3.02	1.01E-07	-2.33	Opposite
ycaC	3.01	1.42E-04	#N/A	New DEG
ydgR	3	7.08E-10	#N/A	New DEG
Z3965	3	5.97E-05	#N/A	New DEG
yodB	2.99	3.60E-04	#N/A	New DEG
epd	2.98	5.33E-12	#N/A	New DEG
gadB	2.96	2.86E-12	-2.02	Opposite
glnH	2.95	1.48E-11	-2.63	Opposite
Z1508	2.95	0.000613	-21.12	Opposite

Z1972	2.93	1.10E-08	-2.56	Opposite
nac	2.93	9.99E-12	-12.62	Opposite
yeaH	2.92	7.17E-08	-3.4	Opposite
cydB	2.87	1.44E-11	#N/A	New DEG
ndh	2.87	6.95E-11	#N/A	New DEG
yhjG	2.85	6.26E-05	#N/A	New DEG
yhjY	2.84	0.000287	#N/A	New DEG
yiaG	2.84	1.32E-03	#N/A	New DEG
glnP	2.84	1.85E-08	-3	Opposite
Z2573	2.83	3.00E-02	#N/A	New DEG
yjbA	2.82	1.06E-06	-2.97	Opposite
Z3260	2.77	1.27E-09	-2.36	Opposite
csgG	2.77	3.83E-08	-2.46	Opposite
Z3595	2.75	2.00E-02	#N/A	New DEG
Z6037	2.73	0.04	#N/A	New DEG
Z2317	2.7	4.00E-02	#N/A	New DEG
ybhO	2.69	0.02	#N/A	New DEG
yeaD	2.67	1.78E-09	-1.97	Opposite
Z1931	2.67	7.79E-11	-2.14	Opposite
pyrI	2.63	9.68E-08	#N/A	New DEG
yagR	2.63	2.00E-02	#N/A	New DEG
Z3074	2.63	2.00E-02	-3.19	Opposite
Z2220	2.62	2.00E-02	#N/A	New DEG
pqqL	2.61	5.29E-09	-3.07	Opposite
yddA	2.6	2.30E-07	-2.39	Opposite
ygaU	2.57	6.71E-07	#N/A	New DEG
Z2186	2.57	0.00439	#N/A	New DEG
hdhA	2.55	6.92E-09	#N/A	New DEG
yjbJ	2.54	1.19E-05	#N/A	New DEG
sodC	2.54	8.15E-03	#N/A	New DEG
ycgK	2.52	0.00099	#N/A	New DEG
himA	2.51	2.29E-09	#N/A	New DEG
yccJ	2.5	2.20E-05	-2.1	Opposite
grxB	2.47	3.81E-08	#N/A	New DEG
add	2.47	1.47E-06	#N/A	New DEG
Z2695	2.44	0.02	#N/A	New DEG
yggB	2.44	1.72E-08	-2.3	Opposite
yhiP	2.43	1.00E-02	#N/A	New DEG
yehZ	2.42	3.00E-02	#N/A	New DEG
Z0967	2.42	0.04	#N/A	New DEG
Z2290	2.42	4.00E-02	#N/A	New DEG
yqjG	2.4	3.85E-03	#N/A	New DEG
bax	2.4	4.51E-08	-1.9	Opposite

phrB	2.38	8.26E-05	#N/A	New DEG
ompC	2.37	1.30E-08	#N/A	New DEG
pspC	2.36	2.21E-04	-3.44	Opposite
Z2883	2.34	3.00E-02	-2.69	Opposite
ybiO	2.32	1.00E-02	#N/A	New DEG
pspA	2.31	1.62E-07	-2.38	Opposite
pyrB	2.3	9.49E-07	#N/A	New DEG
yceK	2.3	0.03	#N/A	New DEG
chaC	2.3	5.26E-06	-3.14	Opposite
sdiA	2.29	2.82E-04	#N/A	New DEG
Z3064	2.29	5.00E-02	#N/A	New DEG
ygbA	2.28	0.00254	#N/A	New DEG
Z2054	2.28	2.77E-07	-1.95	Opposite
ygaE	2.28	2.05E-04	-3.41	Opposite
Z2040	2.27	2.70E-04	#N/A	New DEG
proX	2.26	1.80E-05	#N/A	New DEG
Z1971	2.26	3.00E-02	#N/A	New DEG
Z2301	2.26	0.03	#N/A	New DEG
ycjZ	2.23	1.00E-02	#N/A	New DEG
argT	2.22	0.000000628	-5.58	Opposite
amyA	2.21	5.85E-04	#N/A	New DEG
proV	2.2	1.15E-05	#N/A	New DEG
glnQ	2.19	2.64E-05	-2.37	Opposite
pykF	2.18	8.38E-07	#N/A	New DEG
yhiE	2.17	0.0000228	#N/A	New DEG
cbl	2.17	0.00000777	-10.44	Opposite
yhdW	2.17	0.000324	-15.59	Opposite
p0055	2.15	3.65E-03	#N/A	New DEG
osmC	2.14	2.87E-06	#N/A	New DEG
qseA	2.14	3.92E-03	#N/A	New DEG
proW	2.13	4.34E-04	#N/A	New DEG
osmB	2.13	2.48E-03	#N/A	New DEG
yncB	2.13	1.00E-02	#N/A	New DEG
ycbE	2.13	0.03	#N/A	New DEG
ybdG	2.12	0.000155	#N/A	New DEG
Z2272	2.12	1.00E-02	#N/A	New DEG
ydcE	2.12	0.02	#N/A	New DEG
Z1921	2.12	7.19E-03	-2.4	Opposite
yneJ	2.11	3.00E-02	#N/A	New DEG
yhiW	2.1	0.0000548	#N/A	New DEG
xylE	2.1	7.89E-03	-2.29	Opposite
hmpA	2.09	3.99E-06	#N/A	New DEG
yrbL	2.09	0.000199	#N/A	New DEG

pspD	2.09	5.00E-02	-2.7	Opposite
Z2275	2.08	0.0072	-4.16	Opposite
ybgE	2.07	0.000289	#N/A	New DEG
chaA	2.06	5.12E-05	#N/A	New DEG
pspF	2.06	0.01	#N/A	New DEG
gapA	2.05	4.72E-06	#N/A	New DEG
Z1957	2.05	7.20E-03	#N/A	New DEG
Z2533	2.04	1.63E-03	#N/A	New DEG
glpD	2.04	0.00595	#N/A	New DEG
Z1528	2.03	0.00785	#N/A	New DEG
nrdF	2.02	0.0000118	#N/A	New DEG
nrdH	2.01	3.49E-05	#N/A	New DEG
gapC	2.01	3.51E-03	#N/A	New DEG
Z2585	2.01	0.02	#N/A	New DEG
cls	2.01	2.21E-04	-1.91	Opposite
osmE	2.01	0.000758	-1.95	Opposite
ymcD	2	1.23E-05	#N/A	New DEG
rstB	1.97	0.00782	#N/A	New DEG
xseA	1.95	0.000236	#N/A	New DEG
yjiY	1.95	3.65E-04	#N/A	New DEG
yhdZ	1.95	0.01	-7.99	Opposite
ptsH	1.94	0.0000427	#N/A	New DEG
yeaR	1.94	5.00E-02	#N/A	New DEG
ydeA	1.93	9.88E-03	#N/A	New DEG
ybeJ	1.91	0.000062	#N/A	New DEG
ydiC	1.91	1.68E-04	#N/A	New DEG
yceP	1.91	0.05	#N/A	New DEG
Z2185	1.91	3.00E-02	-2.13	Opposite
metF	1.91	0.03	-4.31	Opposite
ynhE	1.9	0.0000743	#N/A	New DEG
msyB	1.9	0.01	#N/A	New DEG
ybaT	1.89	5.00E-02	#N/A	New DEG
yciE	1.87	4.00E-02	#N/A	New DEG
metK	1.87	1.64E-04	-2.03	Opposite
ptsI	1.86	1.08E-04	#N/A	New DEG
nrdI	1.85	0.000213	#N/A	New DEG
yhbH	1.85	2.30E-04	#N/A	New DEG
yqjE	1.85	2.56E-03	#N/A	New DEG
rstA	1.85	3.00E-02	#N/A	New DEG
cfa	1.84	0.000289	#N/A	New DEG
ynhA	1.84	0.000529	#N/A	New DEG
Z2090	1.84	0.00578	#N/A	New DEG
Z2592	1.83	2.00E-02	#N/A	New DEG

Z2219	1.83	0.03	#N/A	New DEG
metA	1.82	3.00E-02	-2.33	Opposite
proP	1.81	4.94E-04	#N/A	New DEG
ypjA	1.81	0.01	#N/A	New DEG
Z3312	1.81	0.01	#N/A	New DEG
Z1052	1.81	2.00E-02	-2.61	Opposite
nrdE	1.8	0.000319	#N/A	New DEG
Z2386	1.8	0.000544	#N/A	New DEG
rpoS	1.79	3.97E-04	#N/A	New DEG
bolA	1.79	0.00312	#N/A	New DEG
ydaL	1.79	2.00E-02	#N/A	New DEG
Z2755	1.79	0.02	#N/A	New DEG
yjbQ	1.78	0.00317	#N/A	New DEG
uidR	1.78	0.03	#N/A	New DEG
hdeA	1.77	0.00073	#N/A	New DEG
ynhG	1.77	0.00307	#N/A	New DEG
exuR	1.77	0.00405	#N/A	New DEG
ybeL	1.77	1.00E-02	#N/A	New DEG
yifA	1.77	0.01	#N/A	New DEG
yajO	1.77	3.00E-02	#N/A	New DEG
ycfQ	1.77	4.00E-02	#N/A	New DEG
tehB	1.76	0.00523	#N/A	New DEG
pgpB	1.76	0.03	#N/A	New DEG
Z3310	1.76	0.05	#N/A	New DEG
Z2387	1.75	0.00302	#N/A	New DEG
cydC	1.75	3.55E-03	#N/A	New DEG
Z0377	1.75	0.00675	#N/A	New DEG
ydeH	1.75	0.00896	#N/A	New DEG
yciS	1.75	0.01	#N/A	New DEG
dbpA	1.74	0.03	#N/A	New DEG
Z2708	1.73	0.00125	#N/A	New DEG
tehA	1.73	0.02	#N/A	New DEG
Z2099	1.72	0.00128	#N/A	New DEG
Z0609	1.72	0.02	#N/A	New DEG
Z1690	1.72	0.03	#N/A	New DEG
Z2217	1.72	0.05	#N/A	New DEG
ydgA	1.71	0.00384	#N/A	New DEG
yoaE	1.7	0.00565	#N/A	New DEG
Z4882	1.7	0.02	#N/A	New DEG
galR	1.7	0.03	#N/A	New DEG
yfiA	1.69	0.00284	#N/A	New DEG
yciK	1.69	0.02	#N/A	New DEG
ykgA	1.69	0.05	#N/A	New DEG

pyrF	1.68	0.02	#N/A	New DEG
Z0371	1.68	0.02	#N/A	New DEG
Z2419	1.68	0.05	#N/A	New DEG
Z1161	1.67	0.00652	#N/A	New DEG
ytfK	1.67	0.04	#N/A	New DEG
ydfG	1.67	1.00E-02	-2.51	Opposite
Z2267	1.66	0.00296	#N/A	New DEG
ydaA	1.66	0.00749	#N/A	New DEG
Z1600	1.66	0.00749	#N/A	New DEG
potC	1.66	0.03	#N/A	New DEG
Z6075	1.66	0.03	#N/A	New DEG
ydcH	1.66	0.05	#N/A	New DEG
yhiU	1.66	0.05	#N/A	New DEG
metE	1.65	0.00488	-5.17	Opposite
glnA	1.64	0.00429	#N/A	New DEG
ynhD	1.64	0.00597	#N/A	New DEG
cysQ	1.64	0.02	#N/A	New DEG
Z4134	1.64	0.02	#N/A	New DEG
yjcB	1.64	0.04	#N/A	New DEG
yqjD	1.63	0.03	#N/A	New DEG
ycfS	1.63	0.04	#N/A	New DEG
p0061+p0062	1.63	0.000599	#N/A	New DEG
glnG	1.62	0.00952	#N/A	New DEG
Z2691	1.62	0.02	#N/A	New DEG
ygjF	1.62	0.03	#N/A	New DEG
yqjH	1.62	0.03	#N/A	New DEG
Z2735	1.61	0.01	#N/A	New DEG
yiiO	1.61	0.02	#N/A	New DEG
ybaR	1.6	0.02	#N/A	New DEG
rrsA	1.59	0.00896	#N/A	New DEG
yadC	1.58	0.04	#N/A	New DEG
Z1568	1.58	0.04	#N/A	New DEG
gadA	1.58	2.00E-02	-1.89	Opposite
crr	1.57	0.01	#N/A	New DEG
rrsH	1.57	0.01	#N/A	New DEG
Z3658	1.57	0.01	#N/A	New DEG
phoH	1.57	0.02	#N/A	New DEG
pepT	1.57	0.04	#N/A	New DEG
Z1932	1.57	0.05	#N/A	New DEG
cspD	1.56	0.02	#N/A	New DEG
slp	1.56	0.02	#N/A	New DEG
ynhC	1.56	0.02	#N/A	New DEG
ybeC	1.56	0.03	#N/A	New DEG

focA	1.56	0.04	#N/A	New DEG
ygiE	1.56	0.05	#N/A	New DEG
slyB	1.55	0.02	#N/A	New DEG
bglJ	1.55	0.05	#N/A	New DEG
yeeX	1.54	0.02	#N/A	New DEG
dcp	1.54	0.03	#N/A	New DEG
htpX	1.54	0.03	#N/A	New DEG
nlpD	1.53	0.02	#N/A	New DEG
gltJ	1.53	0.04	#N/A	New DEG
minC	1.53	0.04	#N/A	New DEG
cydD	1.53	0.05	#N/A	New DEG
pfkB	1.53	0.05	#N/A	New DEG
aroG	1.51	0.04	#N/A	New DEG
yccA	1.51	0.04	#N/A	New DEG
nadE	1.5	5.00E-02	#N/A	New DEG
acnB	-1.5	0.03	#N/A	New DEG
dnaG	-1.5	0.05	#N/A	New DEG
atpI	-1.52	0.03	#N/A	New DEG
ycdO	-1.53	0.02	#N/A	New DEG
betB	-1.53	0.04	#N/A	New DEG
nusB	-1.53	0.05	#N/A	New DEG
Z0516	-1.53	0.05	#N/A	New DEG
nrdB	-1.54	0.03	#N/A	New DEG
nuoL	-1.54	0.05	#N/A	New DEG
gnd	-1.55	2.00E-02	#N/A	New DEG
mdh	-1.55	0.02	#N/A	New DEG
dapF	-1.55	4.00E-02	#N/A	New DEG
dam	-1.55	0.05	#N/A	New DEG
nuoB	-1.56	4.00E-02	#N/A	New DEG
tagA	-1.56	0.00256	#N/A	New DEG
pepQ	-1.57	0.02	#N/A	New DEG
Z2565	-1.57	2.00E-02	#N/A	New DEG
murI	-1.57	5.00E-02	1.91	Opposite
Z1140	-1.58	5.00E-02	#N/A	New DEG
wecC	-1.59	4.00E-02	#N/A	New DEG
lspA	-1.6	0.05	#N/A	New DEG
ilvD	-1.62	0.01	#N/A	New DEG
prfC	-1.62	0.02	#N/A	New DEG
yigM	-1.62	5.00E-02	#N/A	New DEG
Z1824	-1.62	4.00E-02	-3.41	Further down
Z4846	-1.63	0.02	#N/A	New DEG
nuoC	-1.64	0.01	#N/A	New DEG
ubiE	-1.64	2.00E-02	#N/A	New DEG

yrdC	-1.64	0.04	#N/A	New DEG
purA	-1.66	3.46E-03	#N/A	New DEG
selA	-1.66	2.00E-02	#N/A	New DEG
ycaJ	-1.66	2.00E-02	#N/A	New DEG
yqeF	-1.66	0.02	#N/A	New DEG
Z4849	-1.66	3.00E-02	#N/A	New DEG
yfhJ	-1.66	5.00E-02	#N/A	New DEG
yjgD	-1.66	6.68E-03	2.01	Opposite
Z1211	-1.66	2.00E-02	1.97	Opposite
nrdA	-1.67	3.55E-03	#N/A	New DEG
Z5900	-1.67	0.00738	#N/A	New DEG
ilvE	-1.67	0.00896	#N/A	New DEG
gltA	-1.68	2.59E-03	#N/A	New DEG
Z2869	-1.68	1.00E-02	#N/A	New DEG
livK	-1.68	3.00E-02	#N/A	New DEG
nuoH	-1.68	3.00E-02	#N/A	New DEG
Z1193	-1.68	5.00E-02	2.25	Opposite
Z5899	-1.69	0.00926	#N/A	New DEG
ycdS	-1.69	4.00E-02	#N/A	New DEG
yafB	-1.69	4.00E-02	2.18	Opposite
cycA	-1.69	3.51E-03	-2.38	Further down
nuoF	-1.7	7.17E-03	#N/A	New DEG
Z4650	-1.7	0.02	#N/A	New DEG
wzy	-1.71	0.0055	#N/A	New DEG
sun	-1.71	9.52E-03	#N/A	New DEG
htrL	-1.71	7.69E-03	2.39	Opposite
fis	-1.72	5.80E-03	#N/A	New DEG
atpB	-1.73	0.00125	#N/A	New DEG
btuB	-1.74	1.60E-03	#N/A	New DEG
parE	-1.74	0.00312	#N/A	New DEG
yjeR	-1.74	0.02	#N/A	New DEG
polB	-1.75	0.03	#N/A	New DEG
ruvB	-1.76	2.49E-03	#N/A	New DEG
Z1579	-1.76	5.36E-03	2.13	Opposite
udhA	-1.77	1.12E-03	#N/A	New DEG
nusA	-1.78	7.90E-04	#N/A	New DEG
gidB	-1.78	1.00E-02	1.95	Opposite
evgS	-1.79	0.00932	#N/A	New DEG
ytfL	-1.8	7.14E-03	#N/A	New DEG
fimB	-1.8	1.00E-02	2.43	Opposite
yigI	-1.81	3.00E-02	#N/A	New DEG
yhjE	-1.83	1.12E-03	#N/A	New DEG
exoP	-1.83	3.12E-03	#N/A	New DEG

dnaC	-1.84	1.00E-02	#N/A	New DEG
infB	-1.85	1.63E-04	#N/A	New DEG
gltP	-1.85	1.82E-03	#N/A	New DEG
yaaF	-1.87	3.00E-02	#N/A	New DEG
atpF	-1.89	8.28E-05	#N/A	New DEG
ilvG	-1.89	2.35E-03	#N/A	New DEG
greA	-1.89	4.43E-03	#N/A	New DEG
truB	-1.9	4.94E-04	#N/A	New DEG
sseB	-1.9	6.79E-04	#N/A	New DEG
pta	-1.9	6.70E-05	1.94	Opposite
yhbC	-1.91	1.05E-03	#N/A	New DEG
aroE	-1.91	3.02E-03	#N/A	New DEG
ruvA	-1.91	7.18E-04	1.84	Opposite
livH	-1.92	1.00E-02	#N/A	New DEG
evgA	-1.94	3.74E-03	#N/A	New DEG
recF	-1.95	3.84E-03	#N/A	New DEG
atpH	-1.97	2.20E-05	#N/A	New DEG
pepB	-1.97	7.15E-05	#N/A	New DEG
yjcG	-1.97	1.00E-02	#N/A	New DEG
ackA	-1.97	2.32E-05	2.03	Opposite
atpC	-1.98	1.96E-05	#N/A	New DEG
rbfA	-1.98	0.00114	#N/A	New DEG
putA	-1.98	2.99E-03	#N/A	New DEG
sodA	-1.98	1.84E-05	1.83	Opposite
nuoI	-1.99	4.52E-03	#N/A	New DEG
Z4462	-2	1.41E-04	#N/A	New DEG
mdlB	-2	0.00433	#N/A	New DEG
atpG	-2.01	0.000011	#N/A	New DEG
lysC	-2.01	0.0000824	#N/A	New DEG
yhjW	-2.01	3.00E-02	#N/A	New DEG
glyA	-2.01	9.80E-06	2.26	Opposite
gidA	-2.01	0.0000334	2.08	Opposite
atpE	-2.02	1.03E-05	#N/A	New DEG
leuA	-2.02	2.13E-04	#N/A	New DEG
leuB	-2.03	3.28E-04	#N/A	New DEG
atpA	-2.04	4.96E-06	#N/A	New DEG
phoU	-2.04	1.75E-03	#N/A	New DEG
Z1341	-2.04	1.17E-05	2.08	Opposite
atpD	-2.05	5.11E-06	#N/A	New DEG
rnk	-2.05	1.00E-02	#N/A	New DEG
gspC	-2.05	1.75E-04	#N/A	New DEG
galT	-2.07	4.23E-03	#N/A	New DEG
uvrD	-2.07	2.66E-05	2.5	Opposite

galE	-2.08	0.00112	#N/A	New DEG
ampC	-2.08	3.00E-02	#N/A	New DEG
thrB	-2.1	9.69E-06	#N/A	New DEG
leuC	-2.1	0.0000557	#N/A	New DEG
yfaE	-2.1	0.00353	#N/A	New DEG
cyoE	-2.11	4.43E-06	#N/A	New DEG
Z3071	-2.11	1.81E-04	#N/A	New DEG
Z4855	-2.11	0.05	#N/A	New DEG
thrA	-2.12	0.00000241	#N/A	New DEG
cyoC	-2.12	0.00000765	#N/A	New DEG
uvrB	-2.12	5.56E-06	2.35	Opposite
sucD	-2.12	2.78E-06	2.05	Opposite
thrC	-2.14	4.96E-06	#N/A	New DEG
soxS	-2.15	1.12E-03	2.5	Opposite
Z1020	-2.15	2.87E-04	2.03	Opposite
pnuC	-2.16	1.32E-05	#N/A	New DEG
sbmC	-2.18	1.00E-02	2.41	Opposite
sucC	-2.18	7.62E-07	2	Opposite
dppF	-2.19	0.0000677	#N/A	New DEG
wecF	-2.19	8.73E-03	#N/A	New DEG
fadL	-2.19	8.80E-05	2.15	Opposite
mdaB	-2.2	0.000193	#N/A	New DEG
Z4858	-2.2	0.05	#N/A	New DEG
nuoM	-2.22	1.98E-05	#N/A	New DEG
Z3140	-2.24	0.00000271	#N/A	New DEG
Z2491	-2.24	1.41E-03	2.53	Opposite
Z5117	-2.24	3.00E-02	-2.62	Further down
malT	-2.27	4.68E-07	#N/A	New DEG
deaD	-2.27	2.48E-07	2.22	Opposite
Z0067	-2.28	1.39E-05	2.01	Opposite
hisA	-2.29	7.52E-06	#N/A	New DEG
escT	-2.29	7.78E-03	-2.65	Further down
yeeF	-2.31	6.39E-06	#N/A	New DEG
Z0461	-2.31	7.94E-03	3.74	Opposite
fbp	-2.32	0.000000522	#N/A	New DEG
pstB	-2.32	2.52E-04	2.4	Opposite
dppB	-2.34	0.0000061	#N/A	New DEG
leuD	-2.35	5.96E-05	#N/A	New DEG
gltD	-2.35	8.86E-08	2.05	Opposite
tatD	-2.4	1.17E-03	2.24	Opposite
espB	-2.4	9.65E-09	-2.05	Further down
escU	-2.41	4.99E-03	-2.23	Further down
hisI	-2.42	0.00000041	#N/A	New DEG

yjbO	-2.42	4.10E-04	2.5	Opposite
hisH	-2.43	4.43E-06	#N/A	New DEG
purD	-2.43	1.19E-08	2.19	Opposite
Z4854	-2.44	0.02	#N/A	New DEG
fadB	-2.44	2.03E-05	2.22	Opposite
cyoB	-2.45	6.33E-09	#N/A	New DEG
espA	-2.47	3.36E-09	-2.12	Further down
leuO	-2.47	5.00E-02	-2.41	Further down
cyoA	-2.49	3.86E-09	#N/A	New DEG
dppD	-2.49	0.00000135	#N/A	New DEG
p0045	-2.49	1.16E-04	2.46	Opposite
espD	-2.49	1.87E-09	-2.05	Further down
nadA	-2.51	2.10E-08	#N/A	New DEG
hisB	-2.51	3.72E-08	#N/A	New DEG
cyoD	-2.52	6.95E-08	#N/A	New DEG
yebG	-2.52	0.000000103	3.14	Opposite
purH	-2.52	2.44E-09	2.38	Opposite
Z2751	-2.52	0.000633	2.1	Opposite
yedF	-2.53	2.00E-02	#N/A	New DEG
sucB	-2.53	2.45E-09	2	Opposite
nadB	-2.54	1.37E-08	#N/A	New DEG
hisF	-2.54	1.56E-07	#N/A	New DEG
Z0879	-2.54	0.000203	2.1	Opposite
sucA	-2.56	7.70E-10	1.97	Opposite
sdhC	-2.58	1.11E-06	1.86	Opposite
gltB	-2.6	3.84E-10	2.51	Opposite
dppC	-2.61	6.51E-06	#N/A	New DEG
yjfR	-2.64	2.00E-02	2.62	Opposite
escF	-2.69	1.89E-06	-2.64	Further down
Z2771	-2.7	0.0000139	2.32	Opposite
Z5118	-2.71	2.14E-04	#N/A	New DEG
p0046	-2.72	7.47E-14	2.2	Opposite
Z3109	-2.73	2.02E-03	2.92	Opposite
escN	-2.73	8.79E-09	-1.97	Further down
Z1190	-2.74	6.85E-04	#N/A	New DEG
pckA	-2.77	8.66E-11	2.37	Opposite
yigN	-2.79	6.63E-06	2.92	Opposite
Z5104	-2.81	8.04E-11	-2.2	Further down
acs	-2.82	2.71E-08	2.68	Opposite
uvrA	-2.83	7.46E-11	2.74	Opposite
yafO	-2.84	9.00E-03	#N/A	New DEG
sdhB	-2.91	3.90E-10	2	Opposite
sepL	-2.95	1.41E-11	-1.9	Further down

dctA	-2.96	1.18E-06	#N/A	New DEG
Z5102	-2.96	1.04E-08	-2.52	Further down
mglB	-2.99	3.86E-10	2.39	Opposite
zntA	-3	1.17E-04	#N/A	New DEG
sdhD	-3	2.03E-08	2.47	Opposite
espF	-3.03	5.75E-11	-2.49	Further down
Z3272	-3.04	0.000001	2.06	Opposite
sdhA	-3.07	2.39E-12	1.98	Opposite
escD	-3.1	3.17E-09	-2.23	Further down
gcvH	-3.11	4.66E-09	#N/A	New DEG
Z6011	-3.13	5.00E-02	#N/A	New DEG
Z5137	-3.14	2.00E-10	#N/A	New DEG
Z0721	-3.14	1.37E-03	#N/A	New DEG
afuA	-3.15	1.08E-03	5.66	Opposite
exoU	-3.17	1.80E-03	#N/A	New DEG
Z5123	-3.18	4.93E-11	#N/A	New DEG
yojH	-3.18	2.27E-13	1.88	Opposite
Z5142	-3.19	1.12E-10	-2.15	Further down
Z3933	-3.2	2.00E-02	#N/A	New DEG
ppsA	-3.21	1.34E-14	#N/A	New DEG
Z3916	-3.24	4.56E-03	#N/A	New DEG
hisC	-3.28	7.46E-11	#N/A	New DEG
tir	-3.3	0	-1.77	Further down
Z5138	-3.31	3.41E-10	#N/A	New DEG
gcvP	-3.38	1.23E-14	#N/A	New DEG
yebF	-3.42	3.49E-11	2.66	Opposite
cesD	-3.43	1.79E-11	#N/A	New DEG
Z0955	-3.45	6.72E-15	-2.08	Further down
Z5139	-3.46	1.03E-10	#N/A	New DEG
sepQ	-3.51	1.46E-11	-2.14	Further down
yihN	-3.56	2.00E-02	#N/A	New DEG
escJ	-3.6	5.22E-13	#N/A	New DEG
gcvT	-3.63	5.35E-15	2	Opposite
escC	-3.65	1.73E-15	#N/A	New DEG
dinP	-3.66	9.53E-11	3.91	Opposite
hisD	-3.71	1.90E-13	2.2	Opposite
Z5125	-3.72	2.39E-12	#N/A	New DEG
Z5140	-3.74	2.64E-15	#N/A	New DEG
mglA	-3.81	4.38E-11	2.53	Opposite
Z5143	-3.81	1.70E-04	-2.55	Further down
ordL	-3.82	1.32E-03	2.94	Opposite
lexA	-3.88	5.00E-17	3.8	Opposite
Z5115	-3.89	6.44E-15	#N/A	New DEG

escV	-3.9	2.35E-14	#N/A	New DEG
ilvB	-3.91	2.03E-12	3.5	Opposite
Z5114	-3.94	1.18E-10	#N/A	New DEG
Z2984	-4.04	2.26E-06	#N/A	New DEG
Z5129	-4.07	2.80E-17	#N/A	New DEG
hisG	-4.11	4.18E-15	2.48	Opposite
Z5113	-4.15	7.4E-18	#N/A	New DEG
cspA	-4.36	2.98E-09	3.18	Opposite
Z5136	-4.43	1.18E-15	#N/A	New DEG
Z5111	-4.5	4.49E-21	-2.01	Further down
Z5121	-4.56	5.77E-04	#N/A	New DEG
Z3271	-4.57	3.43E-11	2.65	Opposite
mglC	-4.65	9.59E-11	2.24	Opposite
Z5128	-4.79	2.56E-16	#N/A	New DEG
escR	-4.81	2.32E-10	#N/A	New DEG
ynfM	-4.84	7.55E-16	-3.61	Further down
dinF	-4.9	2.04E-15	3.98	Opposite
sepZ	-4.94	4.38E-23	#N/A	New DEG
Z6024	-5	5.73E-13	-1.95	Further down
eae	-5.07	0	-2.03	Further down
ilvN	-5.08	8.52E-05	2.57	Opposite
Z0315	-5.35	7.03E-08	6.49	Opposite
dinI	-5.65	9.45E-16	4.89	Opposite
coxT	-5.76	5.22E-13	2.36	Opposite
Z2976	-5.96	1.24E-05	5.3	Opposite
umuC	-6.15	2.86E-20	4.92	Opposite
yhaC	-6.25	2.00E-02	14.42	Opposite
yaeG	-6.51	2.18E-21	9.1	Opposite
tnaA	-6.67	9.48E-12	5.72	Opposite
Z5131	-6.78	6.99E-07	#N/A	New DEG
asnB	-6.81	0.00E+00	#N/A	New DEG
recA	-7.01	0.00E+00	5.46	Opposite
Z1450	-7.5	4.20E-03	3.75	Opposite
oraA	-7.8	3.25E-09	5.16	Opposite
escS	-7.87	1.21E-06	#N/A	New DEG
dinD	-7.89	5.51E-18	7.24	Opposite
sulA	-8.31	1.32E-43	6.25	Opposite
umuD	-8.62	3.66E-17	6.64	Opposite
Z0314	-8.68	4.60E-16	10.06	Opposite
Z0316	-9.12	1.65E-22	7.17	Opposite
Z2972	-9.77	1.58E-19	3.88	Opposite
Z2980	-9.8	1.55E-05	6.05	Opposite
yjcH	-10.09	2.58E-04	4.29	Opposite

ygcX	-10.71	6.29E-25	15.2	Opposite
ygcY	-11.49	6.15E-16	10.5	Opposite
recN	-12.32	1.64E-56	8.5	Opposite
asnA	-13.05	1.14E-69	2.35	Opposite
Z0317	-13.91	1.55E-23	8.62	Opposite
Z0312	-14.96	1.35E-15	20.34	Opposite
Z2973	-15.15	6.74E-34	4.89	Opposite
yhaG	-15.99	2.63E-32	12.27	Opposite
Z2971	-18.96	1.06E-53	4.41	Opposite
Z0311	-19.38	5.91E-24	14.4	Opposite
intH	-19.68	3.45E-91	12.17	Opposite
Z2979	-22.43	1.79E-51	21.36	Opposite
Z0313	-22.43	2.11E-17	12.96	Opposite
Z4104	-23.54	5.23E-11	17.02	Opposite
Z0310	-24.37	8.25E-19	61.39	Opposite
Z2975	-28.91	8.85E-19	14.04	Opposite
yhaD	-31.13	8.33E-88	41.23	Opposite
Z2978	-32.53	3.05E-56	17.36	Opposite
Z2974	-48.31	1.27E-63	16.85	Opposite
Z4105	-50.99	5.72E-55	35.2	Opposite
Z2977	-59.71	5.85E-26	43.64	Opposite
yhaE	-91.08	2.49E-119	121.04	Opposite
yhaF	-135.92	5.24E-81	183.86	Opposite
yhaU	-478.93	2.67E-72	75.48	Opposite
Z3626	-685.62	6.50E-39	#N/A	New DEG

Table S4. Bacterial strains used in this study

Strain	Description	Reference
EHEC/WT	*E. coli* TUV93-0	(13)
UPEC	*E. coli* CFT073	(14)
NMEC	*E. coli* CE10	(15)
LCV1	TUV93-0 large colony variant, flase positive for D-ser tolerance	This study
LCV2	TUV93-0 large colony variant, D-ser tolerant	This study
LCV3	TUV93-0 large colony variant, flase positive for D-ser tolerance	This study
LCV4	TUV93-0 large colony variant, D-ser tolerant	This study
LCV5	TUV93-0 large colony variant, D-ser tolerant	This study
LCV6	TUV93-0 large colony variant, D-ser tolerant	This study
LCV7	TUV93-0 large colony variant, D-ser tolerant	This study
LCV8	TUV93-0 large colony variant, D-ser tolerant	This study
LCV9	TUV93-0 large colony variant, D-ser tolerant	This study
LCV10	TUV93-0 large colony variant, D-ser tolerant	This study
LCV10B	TUV93-0 large colony variant, D-ser tolerant	This study
LCV1A	TUV93-0 large colony variant, D-ser tolerant	This study
LCV2A	TUV93-0 large colony variant, D-ser tolerant	This study
Δ*cycA*	TUV93-0 *cycA* deletion mutant	This study
Δ*sstT*	TUV93-0 *sstT* deletion mutant	This study

Δ*cycA*/*sstT*	TUV93-0 mutant lacking both *cycA* and *sstT*	This study
Δ*cscR*	TUV93-0 *cscR* deletion mutant	This study

Table S5. Plasmids used in this study

Plasmid	Description	Reference
pKD46	Lambda red helper plasmid, 30°C temperature sensitive origin, arabinose inducible, ampR	(10)
pKD3	Lambda red PCR template, R6Kγ origin, chlR flanked by FRT sites for removal	(10)
pCP20	FRT recombinase plasmid for excision of chlR from mutants, ampR	(10)
p*rpsM-gfp*	Constitutive *gfp* expression plasmid, chlR	Roe Lab Inventory
pAJR70	Promoterless *gfp* reporter plasmid, chlR	(16)
p*LEE1-gfp*	TUV93-0 *LEE1p* cloned in-frame with *gfp* in pAJR70, chlR	(16)
p*recA-gfp*	K-12 *recAp* cloned in-frame with *gfp* in pUA66, kanR	(2)
pJ241-*rfp*	Shuttle vector with *rfp* under control of *tacp*, kanR	Roe Lab Inventory
pDUAL	Promoterless *gfp* and *rfp* reporter plasmid constructed as outlined in Methods, chlR	This study
p*LEE1-gfp+recA-rfp*	Dual TUV93-0 *LEE1p* and *recAp* reporter plasmid constructed as outlined in Methods, chlR	This study
pACYC184	Shuttle vector with p15A origin, chlR, tetR	(17)
p*cycA*	pACYC184 with *cycA* and native promoter Gibson cloned in place of *tetR*, chlR	This study
p*sstT*	pACYC184 with *sstT* and native promoter Gibson cloned in place of *tetR*, chlR	This study
p*cscR*	pACYC184 with *cscR* and native promoter Gibson cloned in place of *tetR*, chlR	This study
(i)	*cscAp-dsdA* from WT cloned in-frame with *gfp* on pAJR70	This study
(ii)	*cscAp-dsdA* from LCV2 cloned in-frame with *gfp* on pAJR70	This study
(iii)	*z3622p-dsdA* from WT cloned in-frame with *gfp* on pAJR70	This study
(iv)	*z3622p-dsdA* from LCV10B cloned in-frame with *gfp* on pAJR70	This study

ampR: ampicillin resistance; chlR: chloaramphenicol resistance; kanR: kanamycin resistance; tetR: tetracycline resistance

Table S6. Oligonucleotides used in this study

Oligo	Sequence	Use
RFP.pAJR70.Fw	cacacgcatgcATGGTTAGCAAAGGTGAAGAGC	Amplification of promoterless *rfp* from pJ241 for pDUAL (SphI/PaeI)
RFP.pAJR70.Rev	aacgtcgacCGCCTGTCACTTTGCTTGAT	Amplification of promoterless *rfp* from pJ241 for pDUAL (SalI)
PrecA.Fw	aaatcatgaCTCGTGCTGATTATGCCGTG	Amplification of EDL933 *recA* promoter for pDUAL (PagI/BspHI)

PrecA.Rev	cacacgcatgcCTGTTTGTTTTCGTCGATAGCCA	Amplification of EDL933 *recA* promoter for pDUAL (SphI/PaeI)
pDUAL_RFP_Check_For	TATATCGCCGACATCACCGA	Check primers for promoter upstream of *rfp*
pDUAL_RFP_Check_Rev	GTGGTTGTTAACGGTGCCTT	Check primers for promoter upstream of *rfp*
cycA.red.Fw	TAAAGGCCGTAGAGCCTGAACAACACAGACAGGTACAGGAAGAAAAAAACGTGTAGGCTGGAGCTGCTTC	Lamda red EHEC *cycA* deletion cassette amplification
cycA.red.Rev	GAGTGATCTAAAAGCTGGATGGCATTGCGCCATCCAGCATGATAATGCGGCATATGAATATCCTCCTTAG	Lamda red EHEC *cycA* deletion cassette amplification
cycA.check.Fw	CTGTTTCGCGTTATCACCGT	*cycA* deletion check
cycA.check.Rev	CCCGATGATCAAACAAGGCA	*cycA* deletion check
gapA.Q.Fw	TTTCCGTGCTGCTCAGAAAC	*gapA* (control) QPCR primer
gapA.Q.Rev	GGCCGTGAGTGGAGTCATAT	*gapA* (control) QPCR primer
dsdX.rearr.Rev	CGCCTGAACCGATAGCAATC	Check LCV2 and LCV10B rearrangement
Z3622.rearr.Fw	CACGGCAGGATTACGAAACA	Check LCV2 and LCV10B rearrangement
pACYC.lin.Fw	GAACGGGTTGGCATGGATTG	Linearisation of pACYC184, excision of *tetR*
pACYC.lin.Rev	ATCGTATGGGGCTGACTTCA	Linearisation of pACYC184, excision of *tetR*
cycA.184.F	tgaagtcagccccatacgatCTGTTTCGCGTTATCACC	Amplification of cycA with *cycAp* for assembly into linearized pACYC184
cycA.184.R	caatccatgccaacccgttcCCCGATGATCAAACAAGG	Amplification of cycA with *cycAp* for assembly into linearized pACYC184
pACYC.check.F	GACGCTCAAATCAGTGGTGG	Check inserts in pACYC184 complementation assemblies
pACYC.check.R	GCATTCACAGTTCTCCGCAA	Check inserts in pACYC184 complementation assemblies
sstT.red.F	tcgacagaacgcaccagggatgtgcgacaacacaatgaaaggatcgaaaaGTGTAGGCTGGAGCTGCTTC	Lamda red EHEC *sstT* deletion cassette amplification
sstT.red.R	gtttaaagtagagaaaacccctcccgccatagacgaaaggggttaaacaaCATATGAATATCCTCCTTAG	Lamda red EHEC *sstT* deletion cassette amplification
sstT.check.F	ctaagccgtgtaccctgtca	*sstT* deletion check
sstT.check.R	cgcgaagttccatcatcctg	*sstT* deletion check
sstT.184.F	tgaagtcagccccatacgatctaagccgtgtaccctgtca	Amplification of sstT with PsstT for assembly into linearized pACYC184
sstT.184.R	caatccatgccaacccgttccgcgaagttccatcatcctg	Amplification of sstT with PsstT for assembly into linearized pACYC184
sstT.Q.F	GGCATGGATCTCAAAACCCG	*sstT* QPCR primer
sstT.Q.R	CCATCACCAGCATCAACACC	*sstT* QPCR primer
cscR-184-F	tgaagtcagccccatacgatCGCAATAATTTCCGGGCTGA	Amplification of *cscR* for cloning into linearized pACYC184 and deletion check

cscR-184-R	caatccatgccaacccgttcCCCCGCACAATCAGCAATAA	Amplification of *cscR* for cloning into linearized pACYC184 and cdeletion check
cscR-red-F	GTGACGTCTGTTTCTGCTACAGTGCCCGTTTTACGGCAAA CGGCTTGGGTGTGTAGGCTGGAGCTGCTTC	Lamda red EHEC *cscR* deletion cassette amplification
cscR-red-R	AGTGGCTGTGGTGCAACATGGAGCACTCTGGCAACTGG GTTAACATAATACATATGAATATCCTCCTTAG	Lamda red EHEC *cscR* deletion cassette amplification
pAJR70.lin.Fw	GGATCCGGTACCATGGTGAG	Linearisation of pAJR70, adds GSGT to eGFP N-term
pAJR70.lin.Rev	TATATCGCCGACATCACCGA	Linearisation of pAJR70, adds GSGT to eGFP N-term
Pz3622.p70.F	tcggtgatgtcggcgatataGGTGTCGGGCTGTTTCTTTC	Amplification of *z3622p-dsdA* from WT and LCV10B for assembly into pAJR70
PcscA.p70.F	tcggtgatgtcggcgatataATCTACGACCGCATCCCC	Amplification of *cscAp-dsdA* from WT and LCV2 for assembly into pAJR70
dsdA.p70.R	ctcaccatggtaccggatccCTGGGCGATGAGCGAGTTC	Amplification of *Pz3622-dsdA* and *PcscA-dsdA* from WT, LCV2 and LCV10B for assembly into pAJR70
dsdA.Q.F	TGGTTTAATCCTGGCACGAC	UPEC *dsdA* QPCR primer
dsdA.Q.R	CATGGGCGTCCTGAACATC	UPEC *dsdA* QPCR primer
dsdX.Q.F	AAATTCCACCCGTTTCTGGC	UPEC *dsdX* QPCR primer
dsdX.Q.R	CGTTCCGCCAATTCCACTTT	UPEC *dsdX* QPCR primer
dsdC.Q.F	TGTTCGTTCCCATCGCAAAG	UPEC *dsdC* QPCR primer
dsdC.Q.R	GGGTATCCAGCGACGATTTT	UPEC *dsdC* QPCR primer

Supplementary References

1. A. J. Roe, *et al.*, Heterogeneous surface expression of EspA translocon filaments by *Escherichia coli* O157:H7 is controlled at the posttranscriptional Level. *Infect. Immun.* **71**, 5900 LP – 5909 (2003).
2. A. Zaslaver, *et al.*, A comprehensive library of fluorescent transcriptional reporters for Escherichia coli. *Nat. Methods* **3**, 623–628 (2006).
3. A. Platenkamp, J. L. Mellies, Environment controls LEE regulation in enteropathogenic *Escherichia coli*. *Front. Microbiol.* **9**, 1694 (2018).
4. E. M. Phizicky, J. W. Roberts, Induction of SOS functions: Regulation of proteolytic activity of *E. coli* RecA protein by interaction with DNA and

nucleoside triphosphate. *Cell* **25**, 259–267 (1981).

5. A. M. Bolger, M. Lohse, B. Usadel, Trimmomatic: a flexible trimmer for Illumina sequence data. *Bioinformatics* **30**, 2114–2120 (2014).
6. A. Bankevich, *et al.*, SPAdes: A new genome assembly algorithm and its applications to single-cell sequencing. *J. Comput. Biol.* **19**, 455–477 (2012).
7. T. Seemann, Prokka: rapid prokaryotic genome annotation. *Bioinformatics* **30**, 2068–2069 (2014).
8. T. Seemann, Snippy: fast bacterial variant calling from NGS reads (2015).
9. T. Carver, *et al.*, Artemis and ACT: viewing, annotating and comparing sequences stored in a relational database. *Bioinformatics* **24**, 2672–2676 (2008).
10. K. A. Datsenko, B. L. Wanner, One-step inactivation of chromosomal genes in *Escherichia coli* K-12 using PCR products. *Proc. Natl. Acad. Sci.* **97**, 6640 LP – 6645 (2000).
11. A. Santos-Zavaleta, *et al.*, RegulonDB v 10.5: tackling challenges to unify classic and high throughput knowledge of gene regulation in *E. coli* K-12. *Nucleic Acids Res.* **47**, D212–D220 (2018).
12. V. Solovyev, A. Salamov, “Automatic annotation of microbial genomes and metagenomic sequences” in *Metagenomics and Its Applications in Agriculture, Biomedicine and Environmental Studies*, L. R.W, Ed. (Nova Science Publishers, 2011), pp. 61–78.
13. K. G. Campellone, N. Giese, O. J. Tipper, J. M. Leong, A tyrosine-phosphorylated 12-amino-acid sequence of enteropathogenic *Escherichia coli* Tir binds the host adaptor protein Nck and is required for Nck localization to

actin pedestals. *Mol. Microbiol.* **43**, 1227–1241 (2002).

14. H. L. Mobley, *et al.*, Pyelonephritogenic *Escherichia coli* and killing of cultured human renal proximal tubular epithelial cells: role of hemolysin in some strains. *Infect. Immun.* **58**, 1281 LP – 1289 (1990).

15. Y. Yao, Y. Xie, K. S. Kim, Genomic comparison of Escherichia coli K1 strains isolated from the cerebrospinal fluid of patients with meningitis. *Infect Immun* **74** (2006).

16. A. J. Roe, D. E. E. Hoey, D. L. Gally, Regulation, secretion and activity of type III-secreted proteins of enterohaemorrhagic Escherichia coli 0157 in *Biochemical Society Transactions*, (Portland Press Ltd, 2003), pp. 98–103.

17. A. C. Chang, S. N. Cohen, Construction and characterization of amplifiable multicopy DNA cloning vehicles derived from the P15A cryptic miniplasmid. *J. Bacteriol.* **134**, 1141 LP – 1156 (1978).
